# Horizontal gene transfer drives adaptive colonization of apple trees by the fungal pathogen *Valsa mali*

**DOI:** 10.1038/srep33129

**Published:** 2016-09-16

**Authors:** Zhiyuan Yin, Baitao Zhu, Hao Feng, Lili Huang

**Affiliations:** 1 State Key Laboratory of Crop Stress Biology for Arid Areas, College of Plant Protection, Northwest A&F University, Yangling 712100, Shaanxi, China

## Abstract

Horizontal gene transfer (HGT) often has strong benefits for fungi. In a study of samples from apple canker in Shaanxi Province, China, diverse microbes, along with the necrotrophic pathogen *Valsa mali*, were found to colonize the apple bark, thus providing ample opportunity for HGT to occur. In the present study, we identified 32 HGT events in *V. mali* by combining phyletic distribution-based methods with phylogenetic analyses. Most of these HGTs were from bacteria, whereas several others were from eukaryotes. Three HGTs putatively functioned in competition with actinomycetes, some of which showed a significant inhibitory effect on *V. mali*. Three HGTs that were probably involved in nitrogen uptake were also identified. Ten HGTs were thought to be involved in pathogenicity because they were related to known virulence factors, including cell wall-degrading enzymes and candidate effector proteins. HGT14, together with HGT32, was shown to contribute to bleomycin resistance of *V. mali*.These results suggest that HGT drives the adaptive evolution of *V. mali*. The HGTs identified here provide new clues for unveiling the adaptation mechanisms and virulence determinants of *V. mali*.

Horizontal gene transfer (HGT) is the stable transmission of genetic materials between species through any mechanism other than vertical inheritance^1^. HGT plays a significant role in the evolution of prokaryotic lineages, such as by providing novel genes involved in pathogenicity and contributing to adaptive traits^2^,^3^. In eukaryotes, compared with prokaryotes, there is less evidence for functional HGT, but the phenotypic consequences can also be significant in the adaptive evolution of eukaryotes^4^. Hundreds of fungal genomes are now available, and a growing body of data suggest that HGT has had a profound impact on the evolution of pathogenic traits in fungal pathogens^5^,^6^. When considering their functions in infection processes and ecological niches, HGTs in fungi can be divided into the following three categories: those competing with other microbes (antimicrobial genes^7^ and secondary metabolite toxins^1^), those utilizing nutrients (membrane transporter) and those interacting with hosts (reviewed in Soanes and Richards, 2014^6^). Therefore, understanding of functional HGT in fungal pathogens should facilitate the discovery of novel genes involved in niche adaptation, particularly in pathogenicity.

Valsa canker caused by the ascomycetous *Valsa mali*, is one of the most destructive diseases affecting apple trees, causing significant yield losses in eastern Asia^8^,^9^. This necrotrophic pathogen infects trees mainly through wounds and results in severe maceration and necrosis of bark tissues^10^. The dead and dying tissues can be subsequently colonized by diverse saprophytes, thus implying that these microbes occupy the same environment as *V. mali* and therefore may contribute genes to *V. mali* via HGT, because shared habitat is a major factor driving transfers^1^,^5^,^11^. Thus, *V. mali* may have ample opportunity for HGT to occur. We have previously reported the *V. mali* genome sequence, which suggests a potential adaptation to colonize woody bark^12^. In the present study, phyletic distribution-based methods^13^,^14^ and phylogenetic analyses were used to identify potential HGTs in *V. mali*. The functions of two HGTs of interest were verified experimentally. The results will provide new clues for unveiling adaptation mechanisms and virulence determinants in *V. mali*.

## Results and Discussion

### Identification of HGT genes

Identifying HGT in eukaryotes is difficult because of their highly complex genome content. The gold standard for identifying HGT with confidence is phylogenetic incongruence^15^, although its throughput is much lower than that of surrogate methods especially when hundreds of genes or even a genome are being analysed. Thus, we used the effective surrogate tool HGT-Finer^13^ to identify HGT candidates and then verified the results gene-by-gene with phylogenetic analysis. In total, 345 candidates were identified by HGT-Finer, and 32 HGT genes were verified by phylogenetic analyses, manual checking and another phyletic distribution-based method, DarkHorse^14^ (Table 1, Supplementary Files).

Most of these HGTs were derived from bacterial sources (Table 1)^16^, probably because prokaryote-to-fungal HGT is more likely than eukaryote-to-fungal HGT. Consistently with their prokaryotic origin, 18 of the 25 bacterial HGTs had no introns. We also identified five HGTs that were transferred from ascomycetes into bacteria (HGT15, HGT21), basidiomycetes (HGT12, HGT15) and oomycetes (HGT18, HGT20). Compared with prokaryote-fungi transfers, fungi-fungi transfers, especially among closely related species, are difficult to identify because of independent gene loss^17^. Thus, most of the fungi-fungi HGT candidates identified by HGT-Finder were not well supported by phylogenetic analysis. Methods for specifically identifying HGTs among closely related eukaryotes are currently not available.

Functional annotations showed that the HGTs identified mainly affected enzymes (69%) from diverse metabolic pathways and genes (19%) with unknown functions (Table 2). The roles of HGTs in their recipient organisms are often unknown without further experiments^18^. Whether an HGT is expressed under specific conditions might provide clues regarding its adaptive value. Therefore, transcriptomes of *V. mali* during infection of apple bark, reported by us previously^19^, were used to identify HGTs potentially involved in pathogenicity. Six HGTs were strongly upregulated during infection (Table 2), thus suggesting a potential role in *V. mali*-apple interaction. Their specific adaptive values will be discussed in the following section.

### **Putative adaptive value of HGTs in** ***V. mali***

#### Competing with other microbes

**Figure 1:**
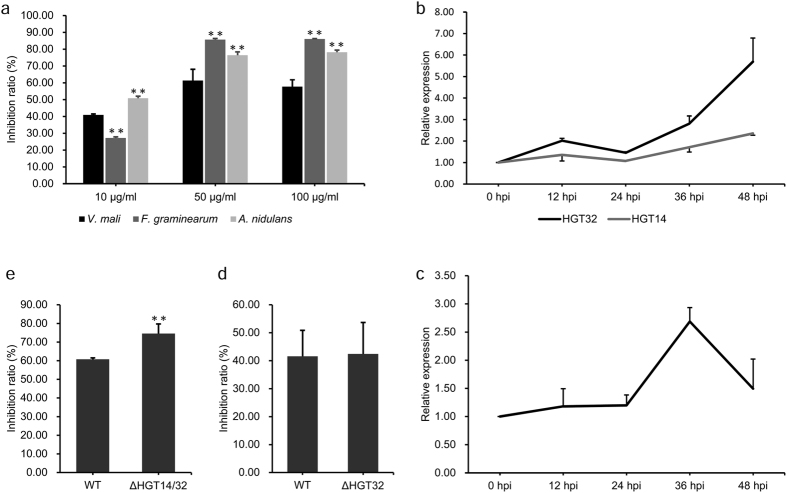
HGT14, together with HGT32, contributes to bleomycin resistance. (**a**) Inhibition ratios of radial growth of three fungi by bleomycin. **(b)** Relative expression of HGT14 and HGT32 genes under bleomycin stress (50 μg/ml). **(c)** Relative expression of HGT14 gene of the HGT32 null mutant under bleomycin stress (50 μg/ml). **(d)** Inhibition ratios of radial growth of the HGT32 null mutant by bleomycin (20 μg/ml). **(e)** Inhibition ratios of radial growth of the HGT14 HGT32 double deletion mutant by bleomycin (50 μg/ml). Bleomycin resistance assays and qRT-PCR analyses were repeated three and three times, respectively. Error bars represent the mean S.D. and asterisks (**) indicate significant differences (*P* < 0.01).

To protect themselves against competitors, microbes often produce secondary metabolite toxins that kill other microbes. The severe maceration and necrosis of apple bark caused by *V. mali* provide ample opportunity for other microbes to colonize. HGT14 and HGT32, bleomycin (Bm) resistance proteins, might enable *V. mali* to be resistant to bleomycin which is a family of antibiotics produced by actinomycetes that cause cell death in eukaryotes and prokaryotes^20^. To test this hypothesis, the capacity of Bm resistance of *V. mali*, *Fusarium graminearum* and *Aspergillus nidulans* was examined under three levels of Bm stress (10, 50 and 100 μg/ml). Compared wiht *F. graminearum* and *A. nidulans*, *V. mali* was significantly more resistant (Figs 1a and S2). RT-qPCR analysis showed that HGT32 but not HGT14 was induced at a high level (50 μg/ml) of Bm stress (Fig. 1b). However, a null mutant of HGT32 did not show reduced resistance (Fig. 1d), and HGT14 of the HGT32 null mutant was induced under Bm stress (50 μg/ml) at 36 hpi (Fig. 1c). Thus, we generated the double deletion mutant of HGT14 and HGT32, which showed a significantly reduced resistance compared with the resistance of the wild type and HGT32 null mutant (Fig. 1e). These results suggest that HGT14, together with HGT32, contributes to Bm-resistance.

Another gene predicted to be associated with competition was HGT5, an *O*-methyltransferase involved in polyketide biosynthesis (Table 2). We reasoned that HGT5 probably functions in competition rather than in synthesizing toxic polyketides, because the genes involved in polyketide biosynthesis are often clustered in fungi and *V. mali* acquired HGT5 as a single gene from actinomycetes. HGT5 in *V. mali* is probably used as a protective device to modify or detoxify specific toxic polyketides produced by actinomycetes, as is the case for *blmB* in the bleomycin biosynthesis gene cluster. The *blmB* gene encodes a *N*-acetyltransferase, which modifies and inactivates bleomycin, conferring self-protection; moreover, other bacteria transformants carrying *blmB* may also gain bleomycin resistance^21^. In addition, three of the five fungi that acquired HGT5 were non-pathogens, including an endophytic *Pestalotiopsis fici*, an endomycorrhizal fungus *Oidiodendron maius* and the saprotrophic *Penicillium roqueforti* (Supplementary Files).

Intriguingly, these three HGTs are all involved in competition with actinomycetes. Indeed, an endophytic actinomycete strain Hhs.015 isolated from cucumber root^22^ significantly inhibits the conidial germination and mycelial growth of *V. mali*^23^. These findings suggest that HGT has driven the adaptive evolution of *V. mali* for competing with other microbes.

#### Nutrient uptake

The types of nutrients that can be utilized by fungi are determined largely by their membrane transporters, because these microbes feed exclusively by osmotrophy. Thus, acquiring a novel transporter gene might enable fungi to utilize a new source of nutrition or gain a competitive advantage against other microbes in their ecological niches. HGT11, a major facilitator superfamily (MFS) transporter of a basidiomycetous source, was found only in the genus *Valsa* (including the pear canker pathogen *V. pyri*) (Tables 1 and 2 and Supplementary Files). HGT11 has significant similarity to the *Saccharomyces cerevisiae* protein DAL5, an allantoate/dipeptide permease (family 2.A.1.14.4) in the Transporter Classification Database. DAL5 transports several substrates and is sensitive to nitrogen catabolite repression^24^,^25^,^26^,^27^,^28^. Thus, by gaining HGT11, *V. mali* might possibly utilize new sources of nitrogen.

Lysine is synthesized *de novo* by the α-aminoadipate (AAA) pathway in higher fungi, and by the diaminopimelate (DAP) pathway in bacteria and plants^29^. The two pathways evolved separately in organisms, and no organism is known to possess both pathways^30^. However, we found that some higher fungi acquired the *DapE* gene (HGT1) in the DAP pathway by horizontal gene transfer (Supplementary Files). HGT1 was transferred from Proteobacteria, which use the succinylase pathway (a variant of the DAP pathway) for lysine biosynthesis^31^. HGT1 was also found in many other fungi including *Colletotrichum graminicola*. The orthologous gene GLRG_10812 was horizontally transferred from bacteria^32^. Further analysis showed that the *V. mali* genome contained all of the essential genes of the AAA pathway and six of the nine genes in the succinylase pathway (Supplementary Table S1). However, in the case of selfish genetic elements^33^, the incomplete DAP pathway might still work through utilizing intermediates synthesized by symbiotic bacteria in the environment, thus providing a strong benefit to acquiring nitrogen from the nitrogen-limited apple bark.

**Figure 2:**
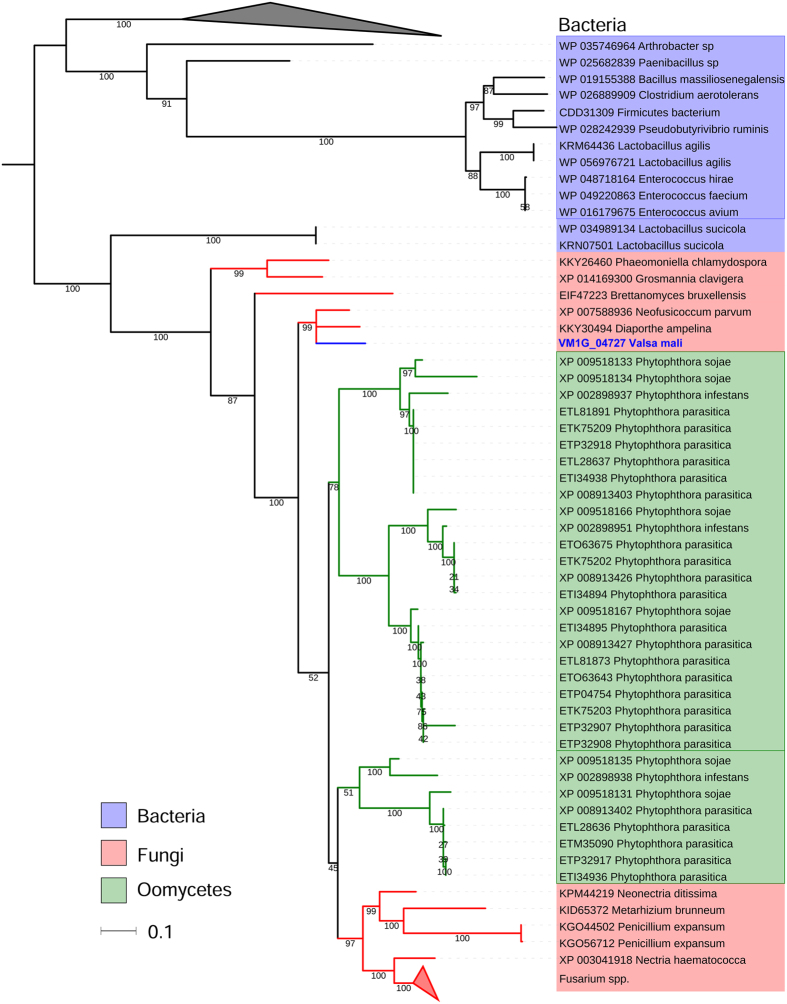
Maximum likelihood phylogenetic tree of HGT20 as an example of fungi-oomycetes HGT. HGT20 was transferred from fungal pathogens into *Phytophthora spp.*

Nitrogen is essential for growth, and disruption of nitrogen nutrition is often associated with the virulence of phytopathogens^34^,^35^. Nitrogen regulation is relatively well studied in yeast and filamentous fungi^35^,^36^. Glutamine and ammonium are preferentially used as nitrogen sources, and the NmrA protein can be activated to repress nitrogen catabolic genes when these sources are sufficient^36^. NmrA is highly conserved in filamentous ascomycetes and is also found in oomycetes which gain NmrA by HGT^37^. In this study, we found an HGT event of fungus-oomycete NmrA (HGT20), which appears to be a potential virulence factor, because both the donor and recipient groups are phytopathogens (Fig. 2). Indeed, the gene that encodes HGT20 was markedly upregulated (log_2_-fold change > 6) during *V. mali* infection (Table 2), thus suggesting that readily assimilated nitrogen sources were sufficient in the infected tissues and that NmrA might be important for regulating selective nitrogen utilization during infection. Consistently with this hypothesis, three of the five ammonia transporter genes have also been found to be significantly upregulated *in planta*^12^. Functions of HGT20 in nitrogen regulation and virulence in *V. mali*, and especially in oomycetes whose nitrogen regulation is poorly understood, are worth investigating.

Additional HGTs predicted to be associated with nutrient uptake include a phosphomannomutase (HGT6) and a uridine nucleosidase (HGT17), which putatively function in the mannose biosynthetic process and nucleotide metabolism, respectively. A putative fructosyl amino acid oxidase (HGT12), which putatively functions in catabolizing naturally occurring fructosyl amino acids^38^, was also identified. Intriguingly, most of these nutrient-related HGTs were predicted to be involved in obtaining nitrogen, whose content in apple bark is relatively low^39^. Therefore, these nitrogen-related HGTs of *V. mali* are possible drivers of adaptive evolution in nitrogen uptake.

#### Interacting with the host

After pathogen attack, many plants produce low molecular weight antimicrobial compounds. Likewise, several cytochrome P450 genes potentially involved in secondary metabolite biosynthesis in apples are upregulated after *V. mali* infection^40^. To counteract these toxic compounds, fungi possess membrane transporters that pump the toxins out of the cell or possess enzymes to detoxify them. Two enzymes with roles in detoxifying phytoalexins are HGT10 (dioxygenase) and HGT28 (thiosulphate sulphurtransferase). HGT10 putatively functions in the degradation of aromatic compounds via its LigB domain. HGT28 is found only in the genus *Valsa* and is a mitochondrial enzyme that detoxifies cyanide^41^. The production of poisonous hydrogen cyanide is known to be used by plants to protect against insects and other herbivorous animals^42^ and has been recently reported to be required for inducible pathogen defence in *Arabidopsis*^43^. To overcome plant cyanogenesis, arthropods have been found to detoxify plant-produced cyanide by acquiring a horizontally transferred gene involved in sulphur amino acid biosynthesis in bacteria^44^. In the current study, we found another HGT event of bacterial origin, which might enable fungi to detoxify cyanide through a different mechanism (HGT28). *V. mali* infects apple trees mainly through wounds^10^, where cyanide production may be induced. Thus, we speculate that the poisonous cyanide itself and its induced defence might possibly be inhibited or overcome by HGT28 if this protein is functional during the *V. mali*-apple interaction.

**Figure 3:**
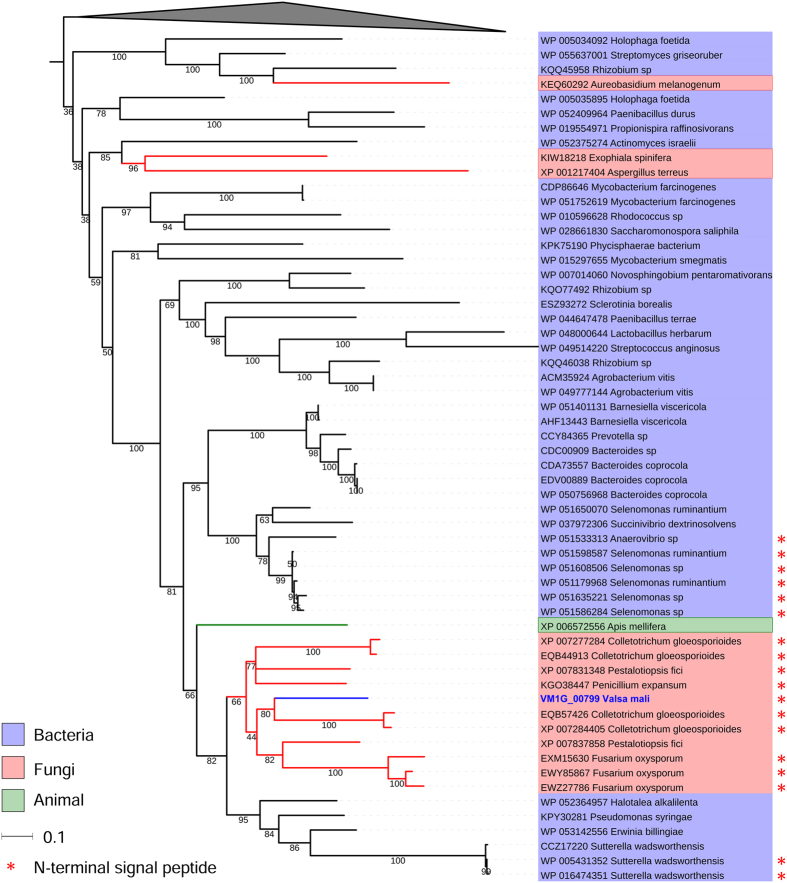
Maximum likelihood phylogenetic tree of HGT4 as an example of bacteria-fungi HGT. Only several proteins from two clades contain the N-terminal signal peptide.

As a typical necrotrophic pathogen on apples, *V. mali* can degrade woody tissues through secreted cell wall-degrading enzymes (CWDEs)^10^,^12^,^19^. Diverse HGTs that putatively function in degrading plant cell wall have been found in eukaryotic plant pathogenic microbes^6^. In the genome of *V. mali*, at least six CWDEs involved in HGT events were identified in the current study (Table 2). Among these, only HGT2 and HGT4 contained the N-terminal signal peptide, thus suggesting that they are probably secreted proteins. However, most of the HGT2 proteins of the donor bacteria and the recipient fungi had no N-terminal signal peptide, except several proteins from two clades, including *V. mali* (Fig. 3). HGT2, a putative hydrolase and peptidase, was strongly activated during infection (Table 2), and null mutants of the HGT2 gene in *V. mali* show a significantly reduced virulence on apple trees (Feng *et al.*, unpublished). These results suggest that HGT2 is a potentially important virulence factor in *V. mali*. Beyond their enzymatic functions, CWDEs can also act as a microbe-associated molecular pattern (MAMP) that activates plant defence responses, as reported for endopolygalacturonases (pectinase) of *Botrytis cinerea*^45^ and a glycoside hydrolase family 12 (GH12) protein of *Phytophthora sojae*^46^. Exploration of the specific functions of HGT2 during *V. mali*-apple interactions is in progress.

Salicylic acid (SA) is an important plant hormone involved in defence responses against biotrophic pathogens^47^. To counteract SA-induced defence responses, pathogens have several weapons that target and disturb SA biosynthesis and signalling, such as salicylate hydroxylase, which degrades SA^48^. Furthermore, genes involved in apple SA signalling are significantly upregulated and enriched after *V. mali* infection^40^. In addition, a putative salicylate hydroxylase (HGT9) transferred from bacteria was identified in *V. mali* (Supplementary Files). Nevertheless, only one of the three salicylate hydroxylases is active in the smut fungus *Ustilago maydis*^49^, and there are also three salicylate hydroxylase homologues in *V. mali* (data not shown). Another protein that probably functions in regulating plant immunity is HGT18, a small secreted protein with unknown function that was strongly activated during infection (Table 2). HGT18 seems to be a candidate effector protein with these characteristics, and it was transferred from fungi to phytopathogenic oomycetes (*Phytophthora* spp.) (Supplementary Files).

## Conclusion

By combing phyletic distribution-based methods and phylogenetic analyses, we identified 32 HGT events in the apple canker pathogen *V. mali*. Most of these HGTs were of bacterial origin, and several were of eukaryotic origin. Three HGTs putatively functioned in competition with actinomycetes some of which showed a significant inhibitory effect on *V. mali*. Three HGTs that are likely to be involved in nitrogen uptake were identified in *V. mali*, which can effectively utilize nitrogen in apple bark. Ten HGTs were thought to be involved in pathogenicity, as they were related to known virulence factors. HGT14, together with HGT32, was shown to contribute to bleomycin resistance of *V. mali*. These results suggest that HGT drives the adaptive evolution of *V. mali*.

## Methods

### Identification of HGT candidates

The proteome of *V. mali*^12^ was searched using blastp (v2.2.30+) against GenBank NR database (data-version 20150706). The blastp output was then subjected to HGT-Finer (*R* threshold ranging from 0.2 to 0.9, *Q* value < 0.01)^13^ to identify HGT candidates. To confirm the results of HGT-Finder, another phyletic distribution-based method, DarkHorse^14^ was used to calculate LPI (lineage probability index) scores of HGTs.

### Phylogenetic analysis

For phylogenetic analysis, the protein sequences of the top 100 blastp hits for each HGT candidate were retrieved from GenBank. Multiple sequence alignments were performed using MAFFT (v7.245)^50^, and poorly aligned regions were removed by trimAl (v1.4)^51^. Maximum likelihood phylogenetic trees were constructed using IQ-TREE (v 1.3.11)^52^ with the best-fit substitution model automatically selected, and branch supports were assessed with ultrafast bootstrap^53^ and SH-aLRT test (1000 replicates). Phylogenetic trees were viewed and produced by iTOL (v2, http://itol.embl.de/)^54^ and FigTree (v1.4.2, http://tree.bio.ed.ac.uk/software/figtree/).

### Functional annotation of HGTs

The putative functions of HGTs were predicted with the Pfam^55^, NCBI CDD^56^ and KEGG^57^ databases. The N-terminal signal peptide was predicted with the SignalP 4.1 server^58^. The expression data were extracted from Yin *et al.*^12^.

### Bleomycin resistance assays

The bleomycin resistance of three fungi (*Valsa mali*, *Aspergillus nidulans* and *Fusarium graminearum*) was evaluated under three levels of bleomycin stress (10, 50 and 100 μg/ml). Fungal isolates were grown on potato dextrose agar (PDA) media at 25 °C in the dark. Diameters of colonies were measured two days post inoculation. Each assay was repeated ten times. The data were analysed with ANOVA or *t*-test by using the online tool VassarStats (http://www.vassarstats.net/).

### Functional studies on bleomycin resistance genes

For RT-qPCR analysis, fungi were grown in potato dextrose broth at 25 °C in the dark for two days. Total RNA was extracted using a Quick RNA isolation Kit (Huayueyang Biotechnology, Beijing, China) according to the manufacturer’s instructions. First strand cDNA synthesis and qPCR were performed using a RevertAid First Strand cDNA Synthesis Kit (Thermo Scientific, Hudson, NH, USA) and RealStar Green Mixture (GenStar, Beijing, China), respectively. The qPCR assays were performed with a CFX96 Connect^TM^ Real-Time System (Bio-Rad, Hercules, CA, USA), and *G6PDH* gene was used as an internal reference^59^. The genes were knocked out according to a previously described method (Figure S3)^60^. Primers used for RT-qPCR and gene deletion are listed in Table S2.

## Additional Information

**How to cite this article**: Yin, Z. *et al.* Horizontal gene transfer drives adaptive colonization of apple trees by the fungal pathogen *Valsa mali. Sci. Rep.* **6**, 33129; doi: 10.1038/srep33129 (2016).

## Supplementary Material

Supplementary Information

## Figures and Tables

**Table 1:** Summary of HGTs identified in *V. mali*.

HGT ID^a^	Seq ID	Length/aa	Intron	SP^b^	Donor group	Recipient taxa	LPI^c^
HGT1	VM1G_00056	456	0	No	Bacteria	Fungi	0.642
HGT2	VM1G_00616	708	0	Yes	Bacteria	Fungi	0.658
HGT3	VM1G_00776	551	0	No	Bacteria	Fungi	0.658
HGT4	VM1G_00799	646	0	Yes	Bacteria	Fungi	0.622
HGT5	VM1G_01172	285	1	No	Bacteria	Fungi	0.628
HGT6	VM1G_01377	249	1	No	Bacteria	Fungi	0.658
HGT7	VM1G_01892	359	0	No	Bacteria	Fungi	0.658
HGT8	VM1G_02284	637	0	No	Basidiomycota	Ascomycota	0.658
HGT9	VM1G_02549	440	1	No	Bacteria	Fungi	0.632
HGT10	VM1G_02555	349	1	No	Bacteria	Fungi	0.623
HGT11	VM1G_02910	476	8	No	Basidiomycota	Ascomycota	0.462
HGT12	VM1G_02970	438	5	No	Ascomycota	Basidiomycota	0.658
HGT13	VM1G_03090	548	0	No	Bacteria	Fungi	0.640
HGT14	VM1G_03108	146	2	No	Bacteria	Fungi	0.594
HGT15	VM1G_03556	300	2	No	Ascomycota	Basidiomycota/Bacteria	0.587
HGT16	VM1G_04133	390	0	No	Bacteria	Fungi	0.594
HGT17	VM1G_04190	285	0	No	Bacteria	Fungi	0.632
HGT18	VM1G_04692	146	2	Yes	Fungi	Oomycetes	0.622
HGT19	VM1G_04714	767	0	No	Bacteria	Fungi	0.658
HGT20	VM1G_04727	317	0	No	Fungi	Oomycetes	0.658
HGT21	VM1G_05155	544	3	No	Fungi	Bacteria	0.640
HGT22	VM1G_05405	840	0	No	Bacteria	Fungi	0.615
HGT23	VM1G_05720	201	0	No	Bacteria	Fungi	0.593
HGT24	VM1G_05918	452	1	No	Bacteria	Fungi	0.628
HGT25	VM1G_07081	319	0	No	Bacteria	Fungi	0.618
HGT26	VM1G_08958	189	0	No	Bacteria	Fungi	0.128
HGT27	VM1G_09114	301	1	No	Bacteria	Fungi	0.658
HGT28	VM1G_10126	333	0	No	Bacteria	Fungi	0.103
HGT29	VM1G_10361	258	0	No	Bacteria	Fungi	0.628
HGT30	VM1G_10685	486	0	No	Bacteria	Fungi	0.128
HGT31	VM1G_10997	252	0	No	Bacteria/Archaea	Fungi	0.108
HGT32	VM1G_05783	420	0	No	Bacteria	Fungi	0.108

^a^HGTs in *V. mali* were identified by the phyletic distribution-based software HGT-Finer^13^ and phylogenetic analyses;

^b^N-terminal signal peptide predicted by SignalP v4.1;

^c^LPI scores (<0.75) calculated by another phyletic distribution method DarkHorse^14^.

**Table 2:** Putative function and transcription profile of HGTs in *V. mali*.

HGT ID	Description	Pfam annotation	Putative function	GFOLD(0.01)^a^	1stRPKM^b^	2ndRPKM^c^
HGT1	Succinyl-diaminopimelate desuccinylase	PF01546: Peptidase_M20	Lysine biosynthesis	0.00	3.88	2.86
HGT2	Six-hairpin glycosidase	PF06202: GDE_C	Cell wall degradation	0.00	0.96	0.27
HGT3	Hypothetical protein	NA	NA	−3.67	10.87	0.00
**HGT4**	**Hypothetical protein**	**PF02129: Peptidase_S15; PF08530: PepX_C**	**Cell wall degradation**	**5.79**	**64.34**	**2527.67**
**HGT5**	**Polyketide synthesis *O*-methyltransferase**	**PF04072: LCM**	**Secondary metabolites biosynthesis**	**1.73**	**34.00**	**87.16**
HGT6	Phosphomannomutase	PF03332: PPM	Mannose biosynthetic process	0.66	189.17	221.25
HGT7	Adenosine deaminase	PF00962: A_deaminase	Purine metabolism	−0.38	24.05	7.10
HGT8	Glucose oxidase	PF00732: GMC_oxred_N; PF05199: GMC_oxred_C	Cell wall degradation	0.37	13.60	16.63
HGT9	Salicylate hydroxylase	PF01494: FAD_binding_3	SA degradation	1.21	0.55	0.88
HGT10	Dioxygenase	PF02900: LigB	Degradation of plant toxin	−1.08	18.57	3.88
HGT11	MFS transporter	PF07690: MFS_1	dipeptide transporter	0.00	0.06	0.00
HGT12	Fructosyl amino acid oxidase	PF01266: DAO	Amino acid metabolism	−0.33	5.78	1.77
HGT13	2-polyprenyl-6-methoxyphenol hydroxylase	PF01494: FAD_binding_3	Ubiquinone biosynthetic pathway	−6.44	398.83	1.94
HGT14	Bleomycin resistance protein	PF12681: Glyoxalase_2	Antibiotics resistance	−0.06	222.73	136.57
HGT15	Short chain dehydrogenase	PF00106: adh_short	NA	0.00	0.09	0.00
HGT16	Pentachlorophenol 4-monooxygenase	PF01494: FAD_binding_3	Ubiquinone biosynthetic pathway	0.20	9.56	11.25
HGT17	Uridine nucleosidase	PF01156: IU_nuc_hydro	Nucleotide metabolism	0.00	0.43	0.00
**HGT18**	**Hypothetical protein**	**NA**	**Candidate effector protein**	**3.37**	**23.89**	**197.11**
**HGT19**	**Beta-L-arabinofuranosidase**	**PF07944: Glyco_hydro_127**	**Cell wall degradation**	**1.80**	**3.27**	**12.24**
**HGT20**	**Quinone oxidoreductase**	**PF05368: NmrA**	**Nitrogen metabolite repression**	**6.57**	**17.02**	**1226.57**
HGT21	Carotenoid oxygenase	PF03055: RPE65	NA	−0.32	38.18	17.69
HGT22	Hypothetical protein	NA	NA	−1.07	2.83	0.32
HGT23	Hypothetical protein	NA	NA	0.00	0.00	0.24
HGT24	N-ethylammeline chlorohydrolase	PF01979: Amidohydro_1	Drug degradation	0.63	3.70	5.67
HGT25	Dioxygenase	PF12697: Abhydrolase_6	NA	−4.93	108.18	0.00
HGT26	Calpastatin	PF08837: DUF1810	NA	0.00	0.05	0.00
HGT27	Esterase/lipase	PF07859: Abhydrolase_3	Cell wall degradation	0.98	14.11	23.78
HGT28	Thiosulfate sulfurtransferase	PF00581: Rhodanese	Cyanide detoxification	−0.31	141.97	70.59
**HGT29**	**5-formyltetrahydrofolate cyclo-ligase**	**PF01812: 5-FTHF_cyc-lig**	**NA**	**2.21**	**1.93**	**9.56**
HGT30	Hydrolase	PF12697: Abhydrolase_6	NA	0.69	1.58	2.09
HGT31	Hypothetical protein	PF07302: AroM	NA	−0.11	59.28	31.09
HGT32	Bleomycin resistance protein	PF12681: Glyoxalase_2	Antibiotics resistance	−0.59	0.72	0.22

^a^Log_2_ (fold-change) calculated by GFOLD with a significant cutoff of 0.01; The transcriptomes of *V. mali* during infection^19^ were re-analysed according to Yin *et al.*^12^;

^b^RPKM of pure mycelium;

^c^RPKM of infected apple bark.
