# Supplementary material for: Horizontal gene transfer drives adaptive colonization of apple trees by the fungal pathogen Valsa mali
Source: Sci Rep. 2016 Sep 16;6:33129. doi: 10.1038/srep33129 (PMC5025739; doi:10.1038/srep33129)
Supplement: Supplementary Information [file srep33129-s1.pdf]

**Supplementary Information**

# Horizontal gene transfer drives adaptive colonization of apple trees by the fungal pathogen *Valsa mali*

Zhiyuan Yin, Baitao Zhu, Hao Feng, Lili Huang*

State Key Laboratory of Crop Stress Biology for Arid Areas and College of Plant Protection, Northwest A&F University, Yangling 712100, Shaanxi, China

*Corresponding author
Lili Huang, e-mail: huanglili@nwsuaf.edu.cn

**Supplementary Figures**

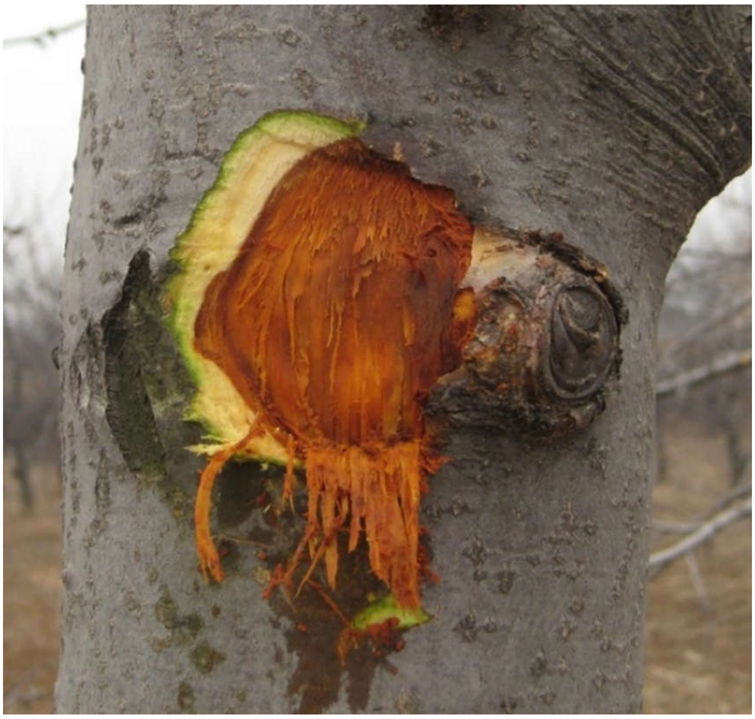

**Supplementary Figure S1. Dead tissues of apple bark caused by *Valsa mali* in the fields.**

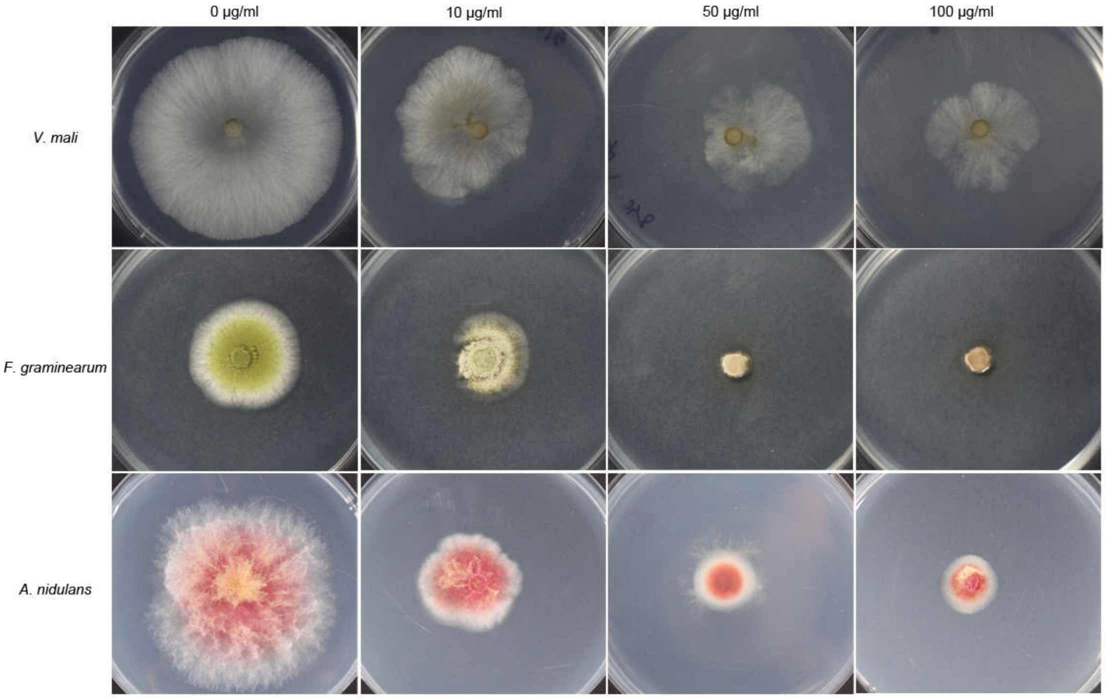


**Supplementary Figure S2. Bleomycin resistance assays of three fungi under different levels of bleomycin stress.** Fungi were grown on the potato dextrose agar (PDA) media at 25 °C in the dark for two days.

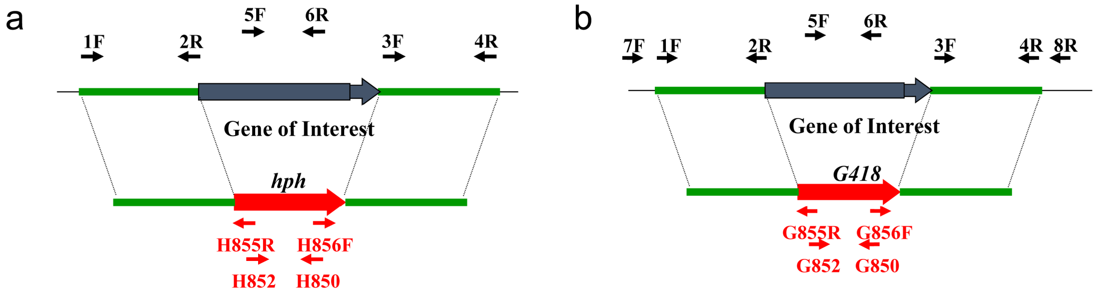


**Supplementary Figure S3. Generation of gene deletion mutants. (a)** Gene replacement strategy of hygromycin-resistant cassette. **(b)** Gene replacement strategy of G418-resistant cassette.

## Supplementary Tables

**Supplementary Table S1. The genes involved in lysine biosynthesis in *V. mali***

| Gene | Description | Ortholog/homolog in *V.mali*[a] |
|---|---|---|
| **Genes involved in α-aminoadipate (AAA) pathway[b]** | | |
| *LYS20*/*LYS21* | Homocitrate synthase | VM1G_07016 |
| *LYS4* | Homoaconitase | VM1G_10252 |
| *LYS12* | Homoisocitrate dehydrogenase | VM1G_06170 |
| *LYS2* | α-Aminoadipate reductase | VM1G_02319, VM1G_10594, VM1G_06405, VM1G_03154, VM1G_05645 |
| *LYS9* | Saccharopine reductase | VM1G_06828 |
| *LYS1* | Saccahropine dehydrogenase | VM1G_01424 |
| **Genes involved in succinylase (DAP) pathway[c]** | | |
| *Ask* | Aspartate kinase | VM1G_06620 |
| *Asd* | Aspartate semialdehyde dehydrogenase | VM1G_08962 |
| *DapA* | Dihydrodipicolinate synthase | VM1G_02804 |
| *DapB* | Dihydrodipicolinate reductase | NA |
| *DapD* | THDPA succinylase | NA |
| *DapC* | succinyl-DAP aminotransferase | VM1G_09511 |
| *DapE* | **succinyl-DAP desuccinylase** | **VM1G_00056** |
| *DapF* | DAP epimerase | NA |
| *LysA* | DAP decarboxylase | VM1G_06998 |

[a]Orthologs to genes involved in AAA pathway of Saccharomyces cerevisiae were identified by orthAgogue (Ekseth O K, Kuiper M, Mironov V. orthAgogue: an agile tool for the rapid prediction of orthology relations. Bioinformatics, 2013: btt582.); homologs to genes involved in DAP pathway were identified by blastp searches using known bacterial genes.

[b]Refer to "Xu H, Andi B, Qian J, et al. The α-aminoadipate pathway for lysine biosynthesis in fungi. Cell biochemistry and biophysics, 2006, 46(1): 43-64."

[c]Refer to "Liu Y, White R H, Whitman W B. Methanococci use the diaminopimelate aminotransferase (DapL) pathway for lysine biosynthesis. Journal of bacteriology, 2010, 192(13): 3304-3310."

**Supplementary Table S2. Primers used for gene deletion and qRT-PCR.**

| Names | Sequences (5'-3') |
|---|---|
| HGT32-1F | CCATTGCGGAGAAGGTTAG |
| HGT32-2R | CAGATACGGCAGAGAAATCGCAACCTCCAAATGCTGCGTGGGTAA |
| HGT32-3F | GTTTAGATTCCAAGTGTCTACTGCTGGCTGGGTAGCGTTGTAGTTGTATG |
| HGT32-4R | TGAGAAGGGAGCCAGCAT |
| HGT32-5F | ACGAATAACTCGCCTGTGG |
| HGT32-6R | AGCCGGGTCCTGAAATG |
| HGT32-7F | GCCAGAGCCAAGTCAACC |
| HGT32-8R | GCCCGTCCAGTATGTAATCA |
| HGT32-CF | GCAACAACCGAAGACGAAA |
| HGT32-CR | CTGACAAGGCAGGAGGAGA |
| G852-F | TCGGCTATGACTGGGCACAACA |
| G850-R | GAGCGGCGATACCGTAAAGCAC |
| G856-F | GAATGGTCAAATCAAACTGCTAGATAT |
| G855-F | TGTTGGGTTTGAGCTAGGTGGG |
| HGT32-qPCR-F | TGGACGATGTAGACGAGGTAT |
| HGT32-qPCR-R | TCCTGAAATGTCACAGTCCTAAC |
| HGT14-1F | GGCTCCAAATCCCCTCG |
| HGT14-2R | TTGACCTCCACTAGCTCCAGCCAAGCCAATACCACTGCTAATACAATGCTG |
| HGT14-3F | GAATAGAGTAGATGCCGACCGCGGGTTGGGAAAGACTACGCCGAATG |
| HGT14-4R | GCGTCCATAGAGCGGAAGAT |
| HGT14-5F | CGTCACTGCCCGTCTCA |
| HGT14-6R | TCCTCAACAGTCAAGTCCCTC |
| HGT14-CF | TCTAGCGAGCCGATTACAGT |
| HGT14-CR | CCCACATTGACCACCCAC |
| H852 | ATGTTGGCGACCTCGTATTGG |
| H850 | TTCCTCCCTTTATTTCAGATTCAA |
| H855-R | GCTGATCTGACCAGTTGC |
| H856-F | GTCGATGCGACGCAATCGT |
| HGT14-qPCR-F | GTGGGAGATATCGAGACGAAAC |
| HGT14-qPCR-R | ATCTGCCAGCCCTTGATTAC |
| G6PDH-F | TCAGAACAAGTTCGAGGGCGACAA |
| G6PDH-R | TGAGGGCAATAGAGGGCTTGTTCA |

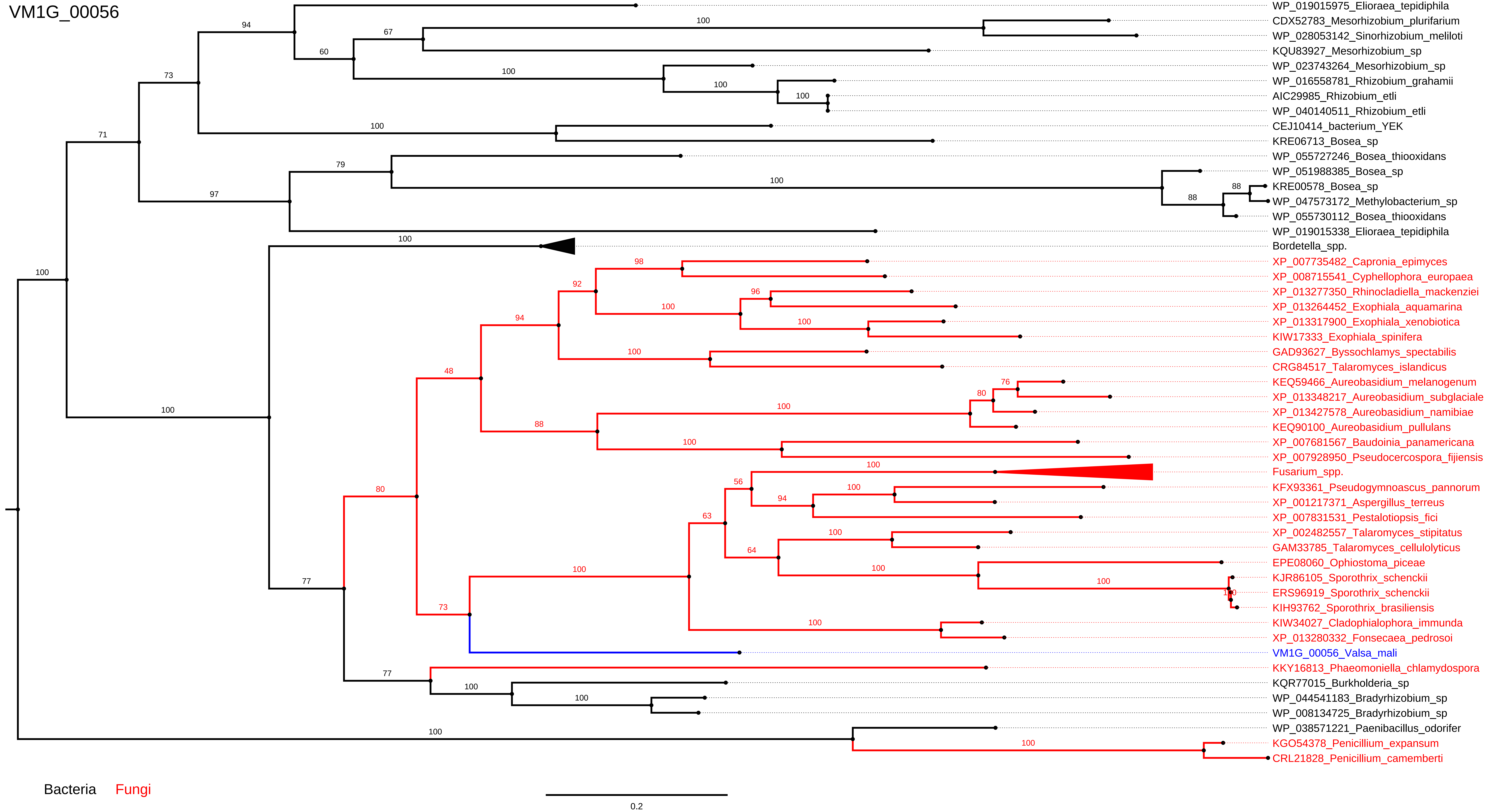
VM1G_00056
WP_019015975_Elioraea_tepidiphila
CDX52783_Mesorhizobium_plurifarium
WP_028053142_Sinorhizobium_meliloti
KQU83927_Mesorhizobium_sp
WP_023743264_Mesorhizobium_sp
WP_016558781_Rhizobium_grahamii
AIC29985_Rhizobium_etli
WP_040140511_Rhizobium_etli
CEJ10414_bacterium_YEK
KRE06713_Bosea_sp
WP_055727246_Bosea_thiooxidans
WP_051988385_Bosea_sp
KRE00578_Bosea_sp
WP_047573172_Methylobacterium_sp
WP_055730112_Bosea_thiooxidans
WP_019015338_Elioraea_tepidiphila
Bordetella_spp.
XP_007735482_Capronia_epimyces
XP_008715541_Cyphellophora_europaea
XP_013277350_Rhinocladiella_mackenziei
XP_013264452_Exophiala_aquamarina
XP_013317900_Exophiala_xenobiotica
KIW17333_Exophiala_spinifera
GAD93627_Byssochlamys_spectabilis
CRG84517_Talaromyces_islandicus
KEQ59466_Aureobasidium_melanogenum
XP_013348217_Aureobasidium_subglaciale
XP_013427578_Aureobasidium_namibiae
KEQ90100_Aureobasidium_pullulans
XP_007681567_Baudoinia_panamericana
XP_007928950_Pseudocercospora_fijiensis
Fusarium_spp.
KFX93361_Pseudogymnoascus_pannorum
XP_001217371_Aspergillus_terreus
XP_007831531_Pestalotiopsis_fici
XP_002482557_Talaromyces_stipitatus
GAM33785_Talaromyces_cellulolyticus
EPE08060_Ophiostoma_piceae
KJR86105_Sporothrix_schenckii
ERS96919_Sporothrix_schenckii
KIH93762_Sporothrix_brasiliensis
KIW34027_Cladophialophora_immunda
XP_013280332_Fonsecaea_pedrosoi
VM1G_00056_Valsa_mali
KKY16813_Phaeomoniella_chlamydospora
KQR77015_Burkholderia_sp
WP_044541183_Bradyrhizobium_sp
WP_008134725_Bradyrhizobium_sp
WP_038571221_Paenibacillus_odorifer
KGO54378_Penicillium_expansum
CRL21828_Penicillium_camemberti
Bacteria
Fungi
0.2

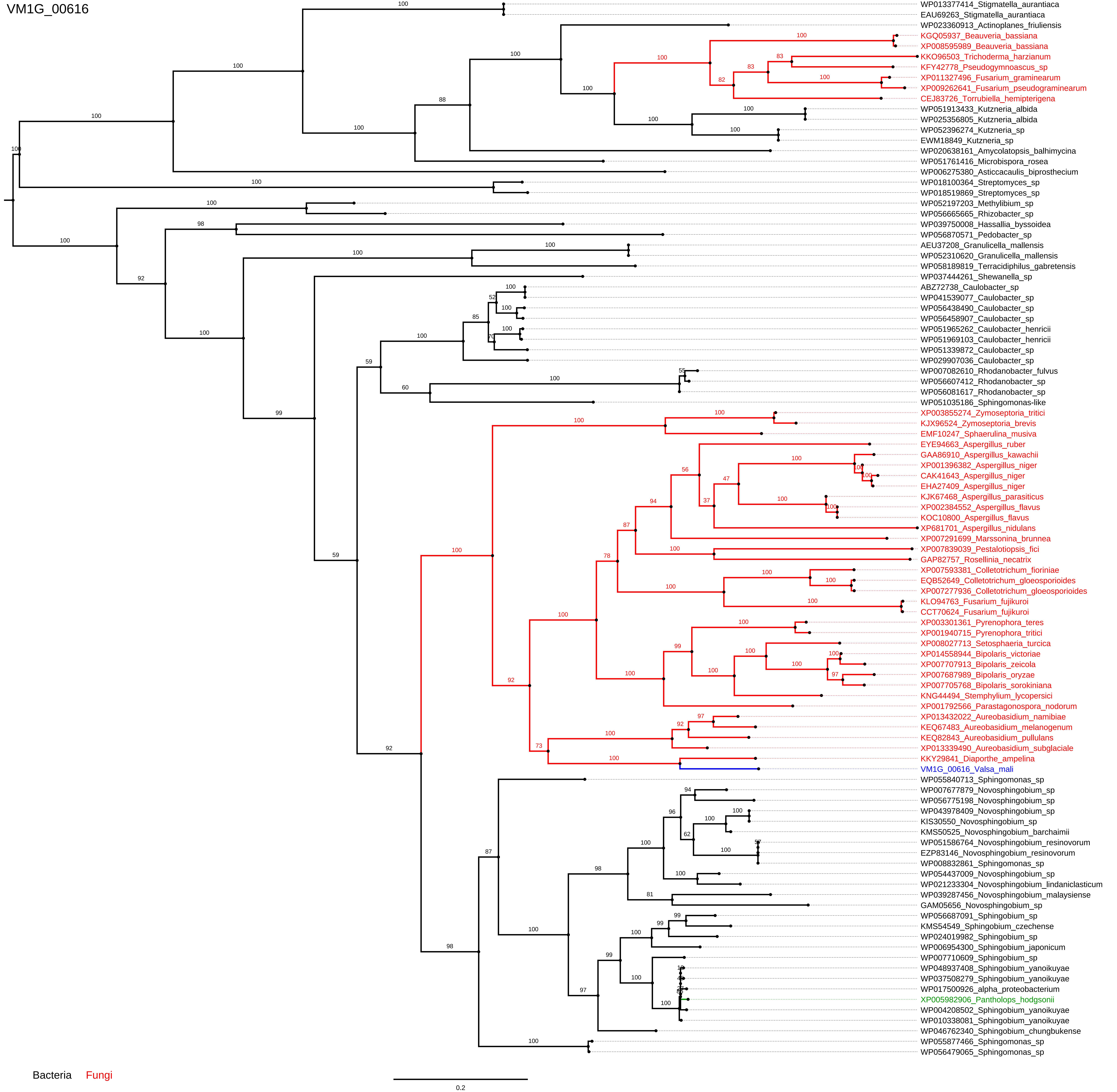

VM1G_00616
WP013377414_Stigmatella_aurantiaca
EAU69263_Stigmatella_aurantiaca
WP023360913_Actinoplanes_friuliensis
KGQ05937_Beauveria_bassiana
XP008595989_Beauveria_bassiana
KKO96503_Trichoderma_harzianum
KFY42778_Pseudogymnoascus_sp
XP011327496_Fusarium_graminearum
XP009262641_Fusarium_pseudograminearum
CEJ83726_Torrubiella_hemipterigena
WP051913433_Kutzneria_albida
WP025356805_Kutzneria_albida
WP052396274_Kutzneria_sp
EWM18849_Kutzneria_sp
WP020638161_Amycolatopsis_balhimycina
WP051761416_Microbispora_rosea
WP006275380_Asticcacaulis_biprosthecium
WP018100364_Streptomyces_sp
WP018519869_Streptomyces_sp
WP052197203_Methylibium_sp
WP056665665_Rhizobacter_sp
WP039750008_Hassallia_byssoidea
WP056870571_Pedobacter_sp
AEU37208_Granulicella_mallensis
WP052310620_Granulicella_mallensis
WP058189819_Terracidiphilus_gabretensis
WP037444261_Shewanella_sp
ABZ72738_Caulobacter_sp
WP041539077_Caulobacter_sp
WP056438490_Caulobacter_sp
WP056458907_Caulobacter_sp
WP051965262_Caulobacter_henricii
WP051969103_Caulobacter_henricii
WP051339872_Caulobacter_sp
WP029907036_Caulobacter_sp
WP007082610_Rhodanobacter_fulvus
WP056607412_Rhodanobacter_sp
WP056081617_Rhodanobacter_sp
WP051035186_Sphingomonas-like
XP003855274_Zymoseptoria_tritici
KJX96524_Zymoseptoria_brevis
EMF10247_Sphaerulina_musiva
EYE94663_Aspergillus_ruber
GAA86910_Aspergillus_kawachii
XP001396382_Aspergillus_niger
CAK41643_Aspergillus_niger
EHA27409_Aspergillus_niger
KJK67468_Aspergillus_parasiticus
XP002384552_Aspergillus_flavus
KOC10800_Aspergillus_flavus
XP681701_Aspergillus_nidulans
XP007291699_Marssonina_brunnea
XP007839039_Pestalotiopsis_fici
GAP82757_Rosellinia_necatrix
XP007593381_Colletotrichum_fioriniae
EQB52649_Colletotrichum_gloeosporioides
XP007277936_Colletotrichum_gloeosporioides
KLO94763_Fusarium_fujikuroi
CCT70624_Fusarium_fujikuroi
XP003301361_Pyrenophora_teres
XP001940715_Pyrenophora_tritici
XP008027713_Setosphaeria_turcica
XP014558944_Bipolaris_victoriae
XP007707913_Bipolaris_zeicola
XP007687989_Bipolaris_oryzae
XP007705768_Bipolaris_sorokiniana
KNG44494_Stemphylium_lycopersici
XP001792566_Parastagonospora_nodorum
XP013432022_Aureobasidium_namibiae
KEQ67483_Aureobasidium_melanogenum
KEQ82843_Aureobasidium_pullulans
XP013339490_Aureobasidium_subglaciale
KKY29841_Diaporthe_ampelina
VM1G_00616_Valsa_mali
WP055840713_Sphingomonas_sp
WP007677879_Novosphingobium_sp
WP056775198_Novosphingobium_sp
WP043978409_Novosphingobium_sp
KIS30550_Novosphingobium_sp
KMS50525_Novosphingobium_barchaimii
WP051586764_Novosphingobium_resinovorum
EZP83146_Novosphingobium_resinovorum
WP008832861_Sphingomonas_sp
WP054437009_Novosphingobium_sp
WP021233304_Novosphingobium_lindaniclasticum
WP039287456_Novosphingobium_malaysiense
GAM05656_Novosphingobium_sp
WP056687091_Sphingobium_sp
KMS54549_Sphingobium_czechense
WP024019982_Sphingobium_sp
WP006954300_Sphingobium_japonicum
WP007710609_Sphingobium_sp
WP048937408_Sphingobium_yanoikuyae
WP037508279_Sphingobium_yanoikuyae
WP017500926_alpha_proteobacterium
XP005982906_Pantholops_hodgsonii
WP004208502_Sphingobium_yanoikuyae
WP010338081_Sphingobium_yanoikuyae
WP046762340_Sphingobium_chungbukense
WP055877466_Sphingomonas_sp
WP056479065_Sphingomonas_sp
Bacteria
Fungi
0.2

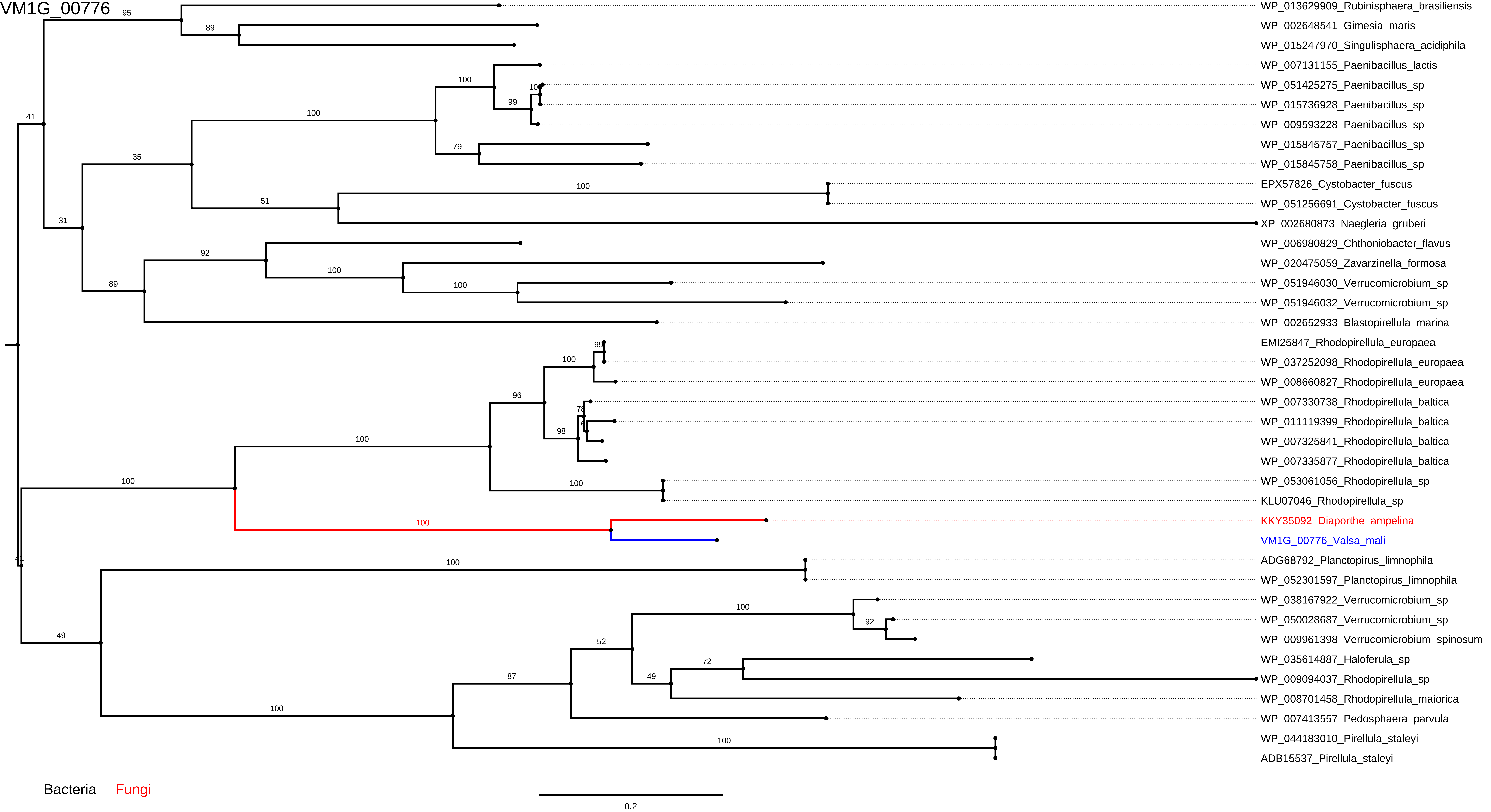
VM1G_00776
WP_013629909_Rubinisphaera_brasiliensis
WP_002648541_Gimesia_maris
WP_015247970_Singulisphaera_acidiphila
WP_007131155_Paenibacillus_lactis
WP_051425275_Paenibacillus_sp
WP_015736928_Paenibacillus_sp
WP_009593228_Paenibacillus_sp
WP_015845757_Paenibacillus_sp
WP_015845758_Paenibacillus_sp
EPX57826_Cystobacter_fuscus
WP_051256691_Cystobacter_fuscus
XP_002680873_Naegleria_gruberi
WP_006980829_Chthoniobacter_flavus
WP_020475059_Zavarzinella_formosa
WP_051946030_Verrucomicrobium_sp
WP_051946032_Verrucomicrobium_sp
WP_002652933_Blastopirellula_marina
EMI25847_Rhodopirellula_europaea
WP_037252098_Rhodopirellula_europaea
WP_008660827_Rhodopirellula_europaea
WP_007330738_Rhodopirellula_baltica
WP_011119399_Rhodopirellula_baltica
WP_007325841_Rhodopirellula_baltica
WP_007335877_Rhodopirellula_baltica
WP_053061056_Rhodopirellula_sp
KLU07046_Rhodopirellula_sp
KKY35092_Diaporthe_ampelina
VM1G_00776_Valsa_mali
ADG68792_Planctopirus_limnophila
WP_052301597_Planctopirus_limnophila
WP_038167922_Verrucomicrobium_sp
WP_050028687_Verrucomicrobium_sp
WP_009961398_Verrucomicrobium_spinosum
WP_035614887_Haloferula_sp
WP_009094037_Rhodopirellula_sp
WP_008701458_Rhodopirellula_maiorica
WP_007413557_Pedosphaera_parvula
WP_044183010_Pirellula_staleyi
ADB15537_Pirellula_staleyi
Bacteria
Fungi
0.2

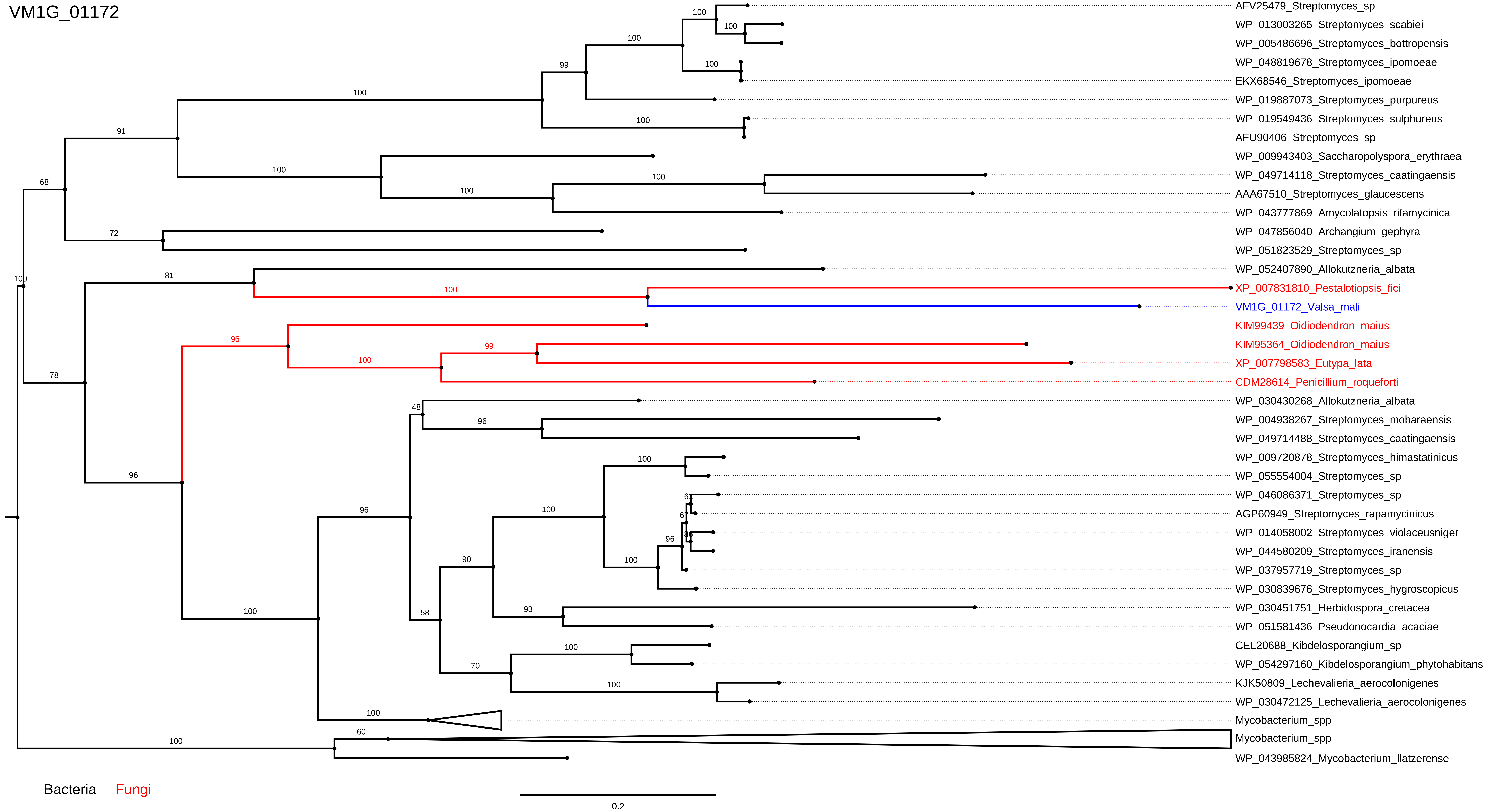
VM1G_01172
AFV25479_Streptomyces_sp
WP_013003265_Streptomyces_scabiei
WP_005486696_Streptomyces_bottropensis
WP_048819678_Streptomyces_ipomoeae
EKX68546_Streptomyces_ipomoeae
WP_019887073_Streptomyces_purpureus
WP_019549436_Streptomyces_sulphureus
AFU90406_Streptomyces_sp
WP_009943403_Saccharopolyspora_erythraea
WP_049714118_Streptomyces_caatingaensis
AAA67510_Streptomyces_glaucescens
WP_043777869_Amycolatopsis_rifamycinica
WP_047856040_Archangium_gephyra
WP_051823529_Streptomyces_sp
WP_052407890_Allokutzneria_albata
XP_007831810_Pestalotiopsis_fici
VM1G_01172_Valsa_mali
KIM99439_Oidiodendron_maius
KIM95364_Oidiodendron_maius
XP_007798583_Eutypa_lata
CDM28614_Penicillium_roqueforti
WP_030430268_Allokutzneria_albata
WP_004938267_Streptomyces_mobaraensis
WP_049714488_Streptomyces_caatingaensis
WP_009720878_Streptomyces_himastatinicus
WP_055554004_Streptomyces_sp
WP_046086371_Streptomyces_sp
AGP60949_Streptomyces_rapamycinicus
WP_014058002_Streptomyces_violaceusniger
WP_044580209_Streptomyces_iranensis
WP_037957719_Streptomyces_sp
WP_030839676_Streptomyces_hygroscopicus
WP_030451751_Herbidospora_cretacea
WP_051581436_Pseudonocardia_acaciae
CEL20688_Kibdelosporangium_sp
WP_054297160_Kibdelosporangium_phytohabitans
KJK50809_Lechevalieria_aerocolonigenes
WP_030472125_Lechevalieria_aerocolonigenes
Mycobacterium_spp
Mycobacterium_spp
WP_043985824_Mycobacterium_llatzerense
Bacteria
Fungi
0.2

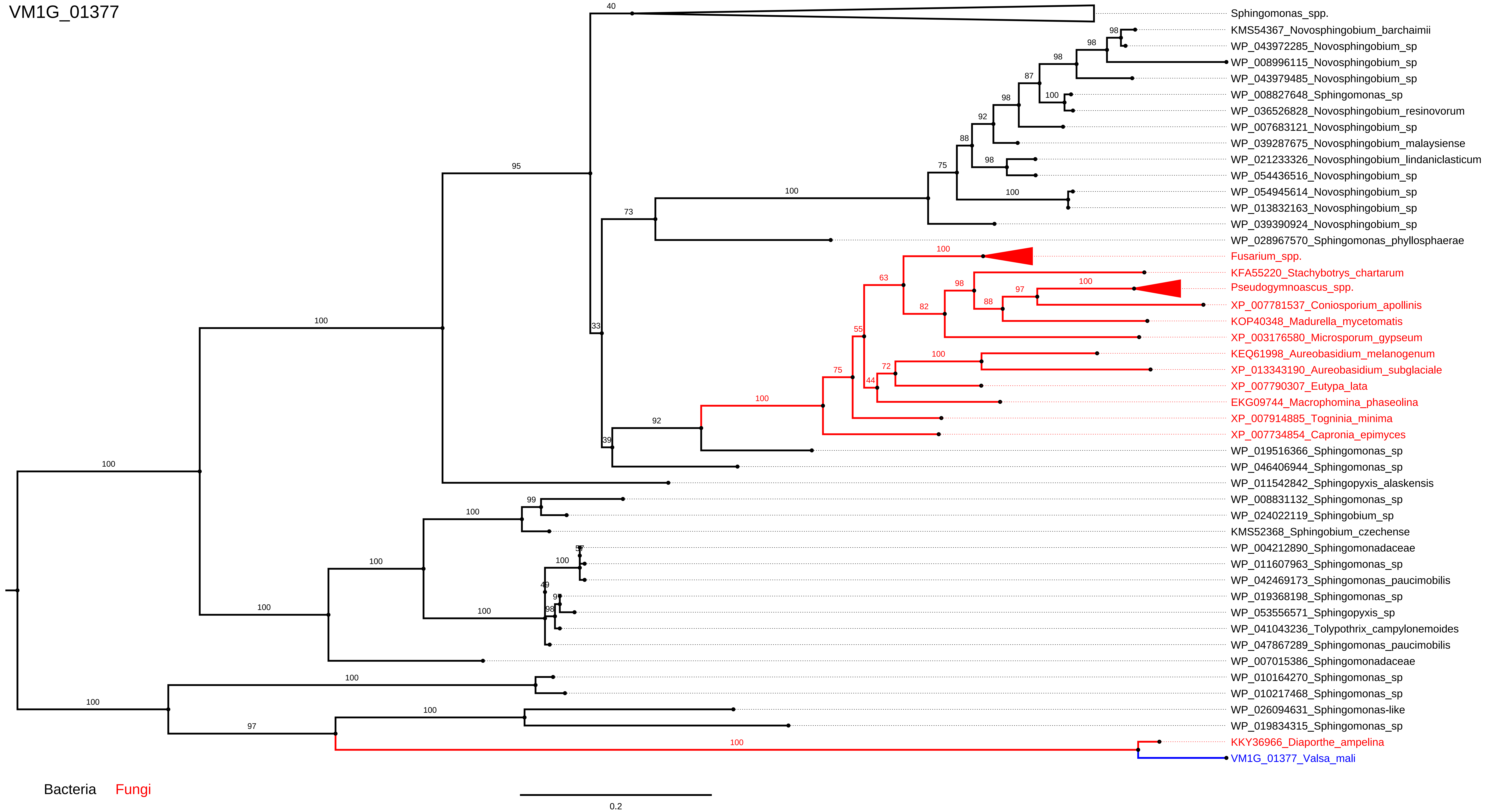
VM1G_01377
Sphingomonas_spp.
KMS54367_Novosphingobium_barchaimii
WP_043972285_Novosphingobium_sp
WP_008996115_Novosphingobium_sp
WP_043979485_Novosphingobium_sp
WP_008827648_Sphingomonas_sp
WP_036526828_Novosphingobium_resinovorum
WP_007683121_Novosphingobium_sp
WP_039287675_Novosphingobium_malaysiense
WP_021233326_Novosphingobium_lindaniclasticum
WP_054436516_Novosphingobium_sp
WP_054945614_Novosphingobium_sp
WP_013832163_Novosphingobium_sp
WP_039390924_Novosphingobium_sp
WP_028967570_Sphingomonas_phyllosphaerae
Fusarium_spp.
KFA55220_Stachybotrys_chartarum
Pseudogymnoascus_spp.
XP_007781537_Coniosporium_apollinis
KOP40348_Madurella_mycetomatis
XP_003176580_Microsporum_gypseum
KEQ61998_Aureobasidium_melanogenum
XP_013343190_Aureobasidium_subglaciale
XP_007790307_Eutypa_lata
EKG09744_Macrophomina_phaseolina
XP_007914885_Togninia_minima
XP_007734854_Capronia_epimyces
WP_019516366_Sphingomonas_sp
WP_046406944_Sphingomonas_sp
WP_011542842_Sphingopyxis_alaskensis
WP_008831132_Sphingomonas_sp
WP_024022119_Sphingobium_sp
KMS52368_Sphingobium_czechense
WP_004212890_Sphingomonadaceae
WP_011607963_Sphingomonas_sp
WP_042469173_Sphingomonas_paucimobilis
WP_019368198_Sphingomonas_sp
WP_053556571_Sphingopyxis_sp
WP_041043236_Tolypothrix_campylonemoides
WP_047867289_Sphingomonas_paucimobilis
WP_007015386_Sphingomonadaceae
WP_010164270_Sphingomonas_sp
WP_010217468_Sphingomonas_sp
WP_026094631_Sphingomonas-like
WP_019834315_Sphingomonas_sp
KKY36966_Diaporthe_ampelina
VM1G_01377_Valsa_mali
Bacteria
Fungi
0.2

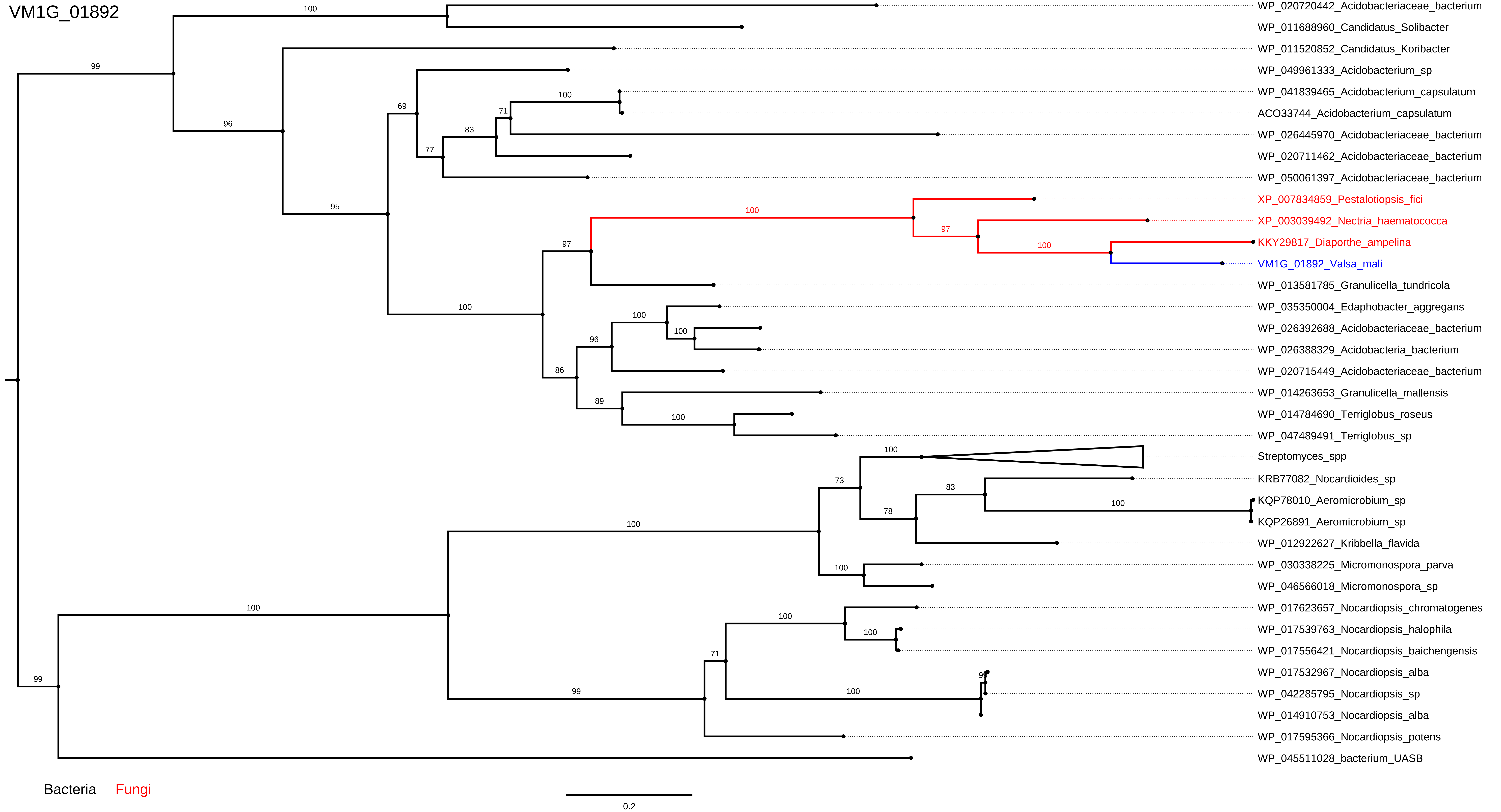
VM1G_01892
WP_020720442_Acidobacteriaceae_bacterium
WP_011688960_Candidatus_Solibacter
WP_011520852_Candidatus_Koribacter
WP_049961333_Acidobacterium_sp
WP_041839465_Acidobacterium_capsulatum
ACO33744_Acidobacterium_capsulatum
WP_026445970_Acidobacteriaceae_bacterium
WP_020711462_Acidobacteriaceae_bacterium
WP_050061397_Acidobacteriaceae_bacterium
XP_007834859_Pestalotiopsis_fici
XP_003039492_Nectria_haematococca
KKY29817_Diaporthe_ampelina
VM1G_01892_Valsa_mali
WP_013581785_Granulicella_tundricola
WP_035350004_Edaphobacter_aggregans
WP_026392688_Acidobacteriaceae_bacterium
WP_026388329_Acidobacteria_bacterium
WP_020715449_Acidobacteriaceae_bacterium
WP_014263653_Granulicella_mallensis
WP_014784690_Terriglobus_roseus
WP_047489491_Terriglobus_sp
Streptomyces_spp
KRB77082_Nocardioides_sp
KQP78010_Aeromicrobium_sp
KQP26891_Aeromicrobium_sp
WP_012922627_Kribbella_flavida
WP_030338225_Micromonospora_parva
WP_046566018_Micromonospora_sp
WP_017623657_Nocardiopsis_chromatogenes
WP_017539763_Nocardiopsis_halophila
WP_017556421_Nocardiopsis_baichengensis
WP_017532967_Nocardiopsis_alba
WP_042285795_Nocardiopsis_sp
WP_014910753_Nocardiopsis_alba
WP_017595366_Nocardiopsis_potens
WP_045511028_bacterium_UASB
Bacteria
Fungi
0.2

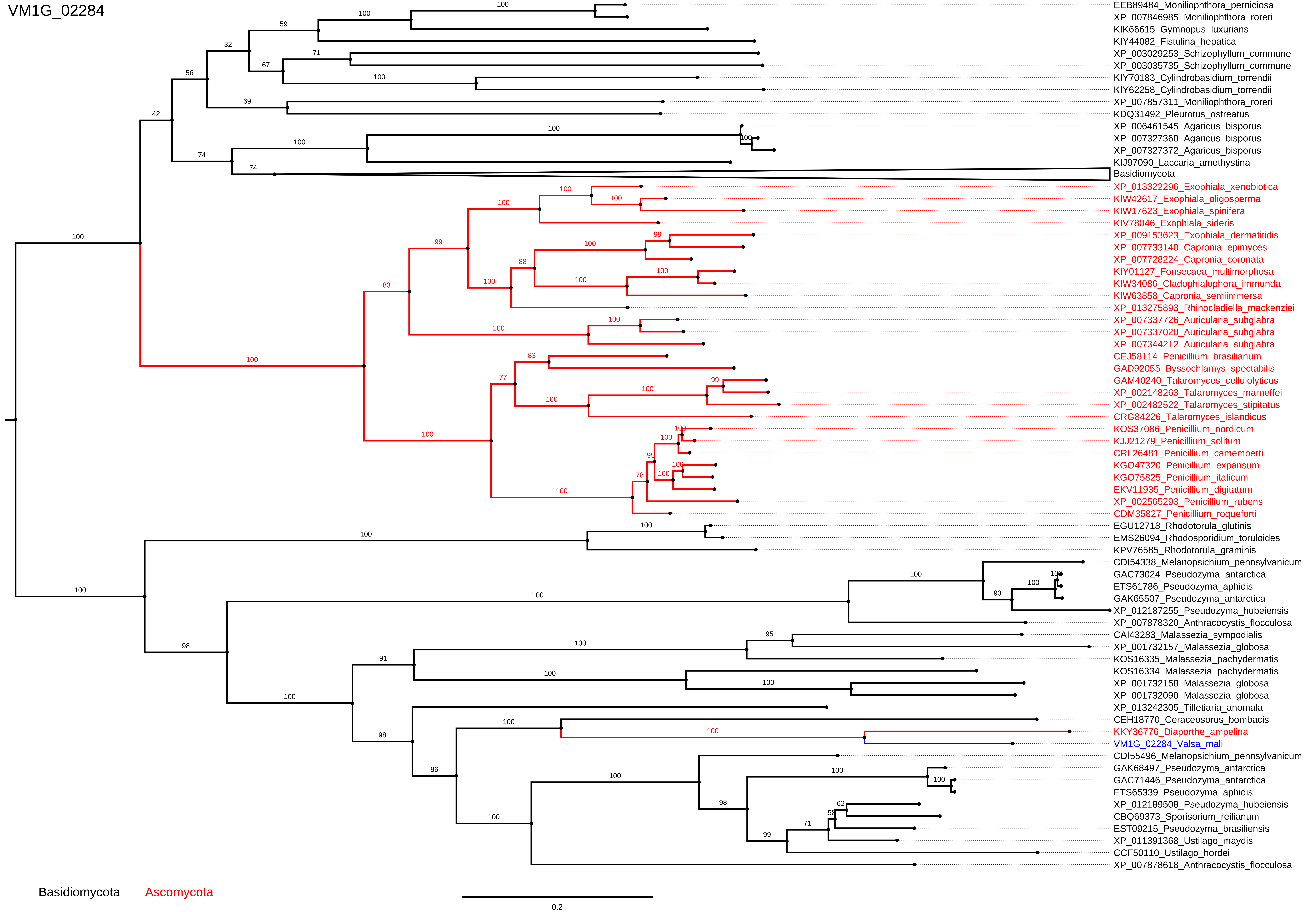

VM1G_02284
EEB89484_Moniliophthora_perniciosa
XP_007846985_Moniliophthora_roreri
KIK66615_Gymnopus_luxurians
KIY44082_Fistulina_hepatica
XP_003029253_Schizophyllum_commune
XP_003035735_Schizophyllum_commune
KIY70183_Cylindrobasidium_torrendii
KIY62258_Cylindrobasidium_torrendii
XP_007857311_Moniliophthora_roreri
KDQ31492_Pleurotus_ostreatus
XP_006461545_Agaricus_bisporus
XP_007327360_Agaricus_bisporus
XP_007327372_Agaricus_bisporus
KIJ97090_Laccaria_amethystina
Basidiomycota
XP_013322296_Exophiala_xenobiotica
KIW42617_Exophiala_oligosperma
KIW17623_Exophiala_spinifera
KIV78046_Exophiala_sideris
XP_009153623_Exophiala_dermatitidis
XP_007733140_Capronia_epimyces
XP_007728224_Capronia_coronata
KIY01127_Fonsecaea_multimorphosa
KIW34086_Cladophialophora_immunda
KIW63858_Capronia_semiimmersa
XP_013275893_Rhinocladiella_mackenziei
XP_007337726_Auricularia_subglabra
XP_007337020_Auricularia_subglabra
XP_007344212_Auricularia_subglabra
CEJ58114_Penicillium_brasilianum
GAD92055_Byssochlamys_spectabilis
GAM40240_Talaromyces_cellulolyticus
XP_002148263_Talaromyces_marneffei
XP_002482522_Talaromyces_stipitatus
CRG84226_Talaromyces_islandicus
KOS37086_Penicillium_nordicum
KJJ21279_Penicillium_solitum
CRL26481_Penicillium_camemberti
KGO47320_Penicillium_expansum
KGO75825_Penicillium_italicum
EKV11935_Penicillium_digitatum
XP_002565293_Penicillium_rubens
CDM35827_Penicillium_roqueforti
EGU12718_Rhodotorula_glutinis
EMS26094_Rhodosporidium_toruloides
KPV76585_Rhodotorula_graminis
CDI54338_Melanopsichium_pennsylvanicum
GAC73024_Pseudozyma_antarctica
ETS61786_Pseudozyma_aphidis
GAK65507_Pseudozyma_antarctica
XP_012187255_Pseudozyma_hubeiensis
XP_007878320_Anthracocystis_flocculosa
CAI43283_Malassezia_sympodialis
XP_001732157_Malassezia_globosa
KOS16335_Malassezia_pachydermatis
KOS16334_Malassezia_pachydermatis
XP_001732158_Malassezia_globosa
XP_001732090_Malassezia_globosa
XP_013242305_Tilletiaria_anomala
CEH18770_Ceraceosorus_bombacis
KKY36776_Diaporthe_ampelina
VM1G_02284_Valsa_mali
CDI55496_Melanopsichium_pennsylvanicum
GAK68497_Pseudozyma_antarctica
GAC71446_Pseudozyma_antarctica
ETS65339_Pseudozyma_aphidis
XP_012189508_Pseudozyma_hubeiensis
CBQ69373_Sporisorium_reilianum
EST09215_Pseudozyma_brasiliensis
XP_011391368_Ustilago_maydis
CCF50110_Ustilago_hordei
XP_007878618_Anthracocystis_flocculosa
Basidiomycota
Ascomycota
0.2

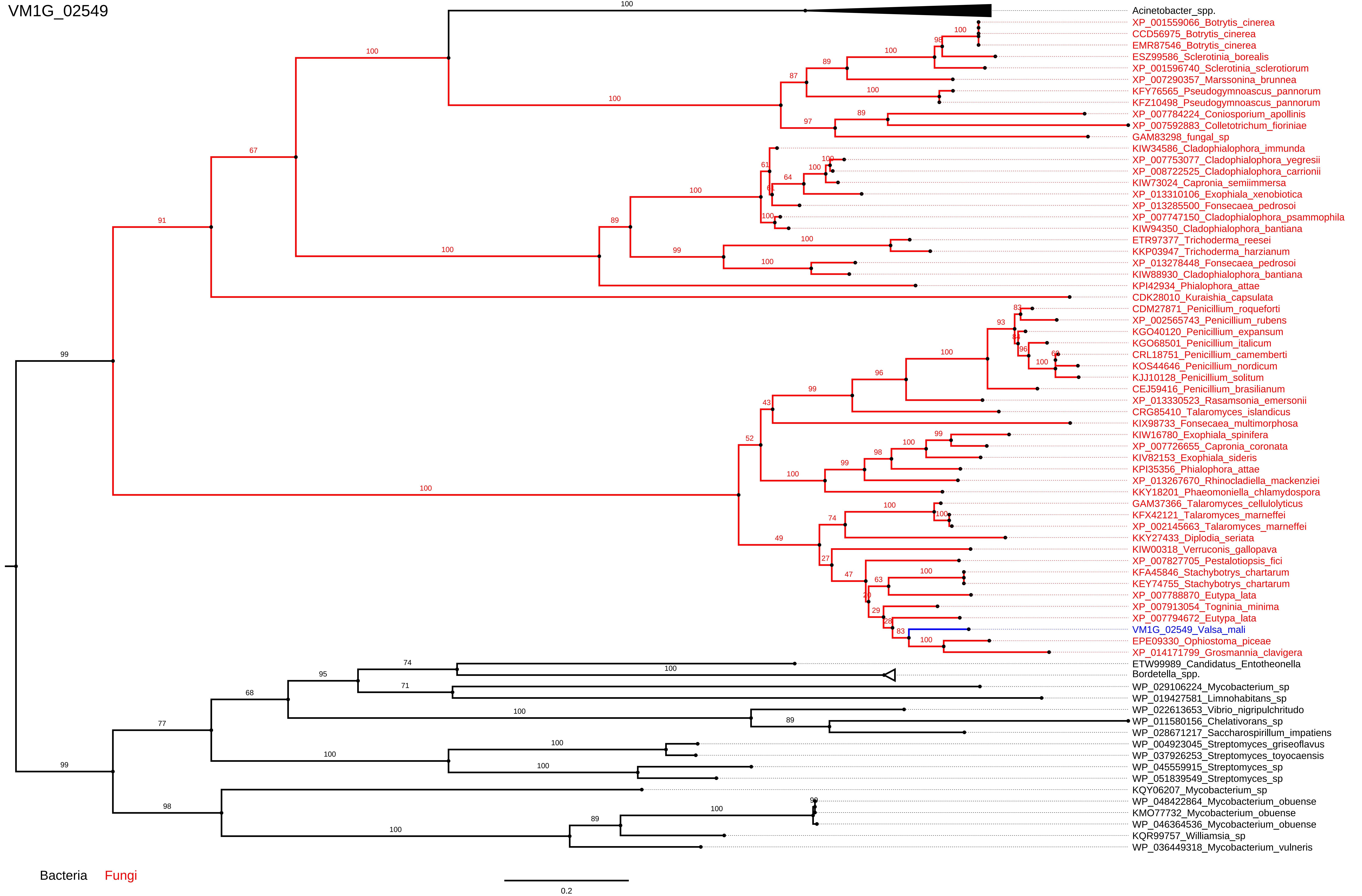

VM1G_02549
Acinetobacter_spp.
XP_001559066_Botrytis_cinerea
CCD56975_Botrytis_cinerea
EMR87546_Botrytis_cinerea
ESZ99586_Sclerotinia_borealis
XP_001596740_Sclerotinia_sclerotiorum
XP_007290357_Marssonina_brunnea
KFY76565_Pseudogymnoascus_pannorum
KFZ10498_Pseudogymnoascus_pannorum
XP_007784224_Coniosporium_apollinis
XP_007592883_Colletotrichum_fioriniae
GAM83298_fungal_sp
KIW34586_Cladophialophora_immunda
XP_007753077_Cladophialophora_yegresii
XP_008722525_Cladophialophora_carrionii
KIW73024_Capronia_semiimmersa
XP_013310106_Exophiala_xenobiotica
XP_013285500_Fonsecaea_pedrosoi
XP_007747150_Cladophialophora_psammophila
KIW94350_Cladophialophora_bantiana
ETR97377_Trichoderma_reesei
KKP03947_Trichoderma_harzianum
XP_013278448_Fonsecaea_pedrosoi
KIW88930_Cladophialophora_bantiana
KPI42934_Phialophora_attae
CDK28010_Kuraishia_capsulata
CDM27871_Penicillium_roqueforti
XP_002565743_Penicillium_rubens
KGO40120_Penicillium_expansum
KGO68501_Penicillium_italicum
CRL18751_Penicillium_camemberti
KOS44646_Penicillium_nordicum
KJJ10128_Penicillium_solitum
CEJ59416_Penicillium_brasilianum
XP_013330523_Rasamsonia_emersonii
CRG85410_Talaromyces_islandicus
KIX98733_Fonsecaea_multimorphosa
KIW16780_Exophiala_spinifera
XP_007726655_Capronia_coronata
KIV82153_Exophiala_sideris
KPI35356_Phialophora_attae
XP_013267670_Rhinocladiella_mackenziei
KKY18201_Phaeomoniella_chlamydospora
GAM37366_Talaromyces_cellulolyticus
KFX42121_Talaromyces_marneffei
XP_002145663_Talaromyces_marneffei
KKY27433_Diplodia_seriata
KIW00318_Verruconis_gallopava
XP_007827705_Pestalotiopsis_fici
KFA45846_Stachybotrys_chartarum
KEY74755_Stachybotrys_chartarum
XP_007788870_Eutypa_lata
XP_007913054_Togninia_minima
XP_007794672_Eutypa_lata
VM1G_02549_Valsa_mali
EPE09330_Ophiostoma_piceae
XP_014171799_Grosmannia_clavigera
ETW99989_Candidatus_Entotheonella
Bordetella_spp.
WP_029106224_Mycobacterium_sp
WP_019427581_Limnohabitans_sp
WP_022613653_Vibrio_nigripulchritudo
WP_011580156_Chelativorans_sp
WP_028671217_Saccharospirillum_impatiens
WP_004923045_Streptomyces_griseoflavus
WP_037926253_Streptomyces_toyocaensis
WP_045559915_Streptomyces_sp
WP_051839549_Streptomyces_sp
KQY06207_Mycobacterium_sp
WP_048422864_Mycobacterium_obuense
KMO77732_Mycobacterium_obuense
WP_046364536_Mycobacterium_obuense
KQR99757_Williamsia_sp
WP_036449318_Mycobacterium_vulneris
Bacteria
Fungi
0.2

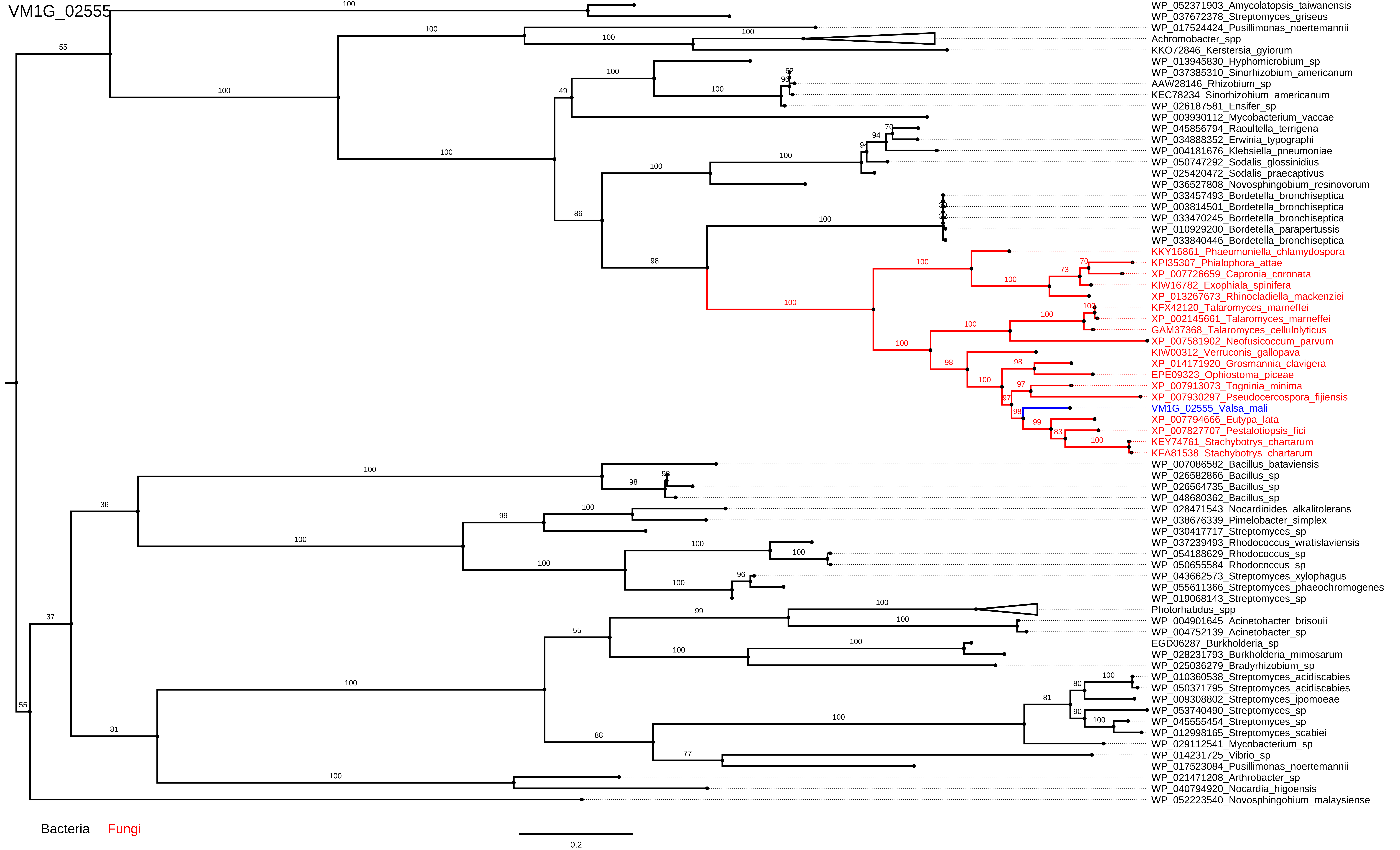
VM1G_02555
WP_052371903_Amycolatopsis_taiwanensis
WP_037672378_Streptomyces_griseus
WP_017524424_Pusillimonas_noertemannii
Achromobacter_spp
KKO72846_Kerstersia_gyiorum
WP_013945830_Hyphomicrobium_sp
WP_037385310_Sinorhizobium_americanum
AAW28146_Rhizobium_sp
KEC78234_Sinorhizobium_americanum
WP_026187581_Ensifer_sp
WP_003930112_Mycobacterium_vaccae
WP_045856794_Raoultella_terrigena
WP_034888352_Erwinia_typographi
WP_004181676_Klebsiella_pneumoniae
WP_050747292_Sodalis_glossinidius
WP_025420472_Sodalis_praecaptivus
WP_036527808_Novosphingobium_resinovorum
WP_033457493_Bordetella_bronchiseptica
WP_003814501_Bordetella_bronchiseptica
WP_033470245_Bordetella_bronchiseptica
WP_010929200_Bordetella_parapertussis
WP_033840446_Bordetella_bronchiseptica
KKY16861_Phaeomoniella_chlamydospora
KPI35307_Phialophora_attae
XP_007726659_Capronia_coronata
KIW16782_Exophiala_spinifera
XP_013267673_Rhinocladiella_mackenziei
KFX42120_Talaromyces_marneffei
XP_002145661_Talaromyces_marneffei
GAM37368_Talaromyces_cellulolyticus
XP_007581902_Neofusicoccum_parvum
KIW00312_Verruconis_gallopava
XP_014171920_Grosmannia_clavigera
EPE09323_Ophiostoma_piceae
XP_007913073_Togninia_minima
XP_007930297_Pseudocercospora_fijiensis
VM1G_02555_Valsa_mali
XP_007794666_Eutypa_lata
XP_007827707_Pestalotiopsis_fici
KEY74761_Stachybotrys_chartarum
KFA81538_Stachybotrys_chartarum
WP_007086582_Bacillus_bataviensis
WP_026582866_Bacillus_sp
WP_026564735_Bacillus_sp
WP_048680362_Bacillus_sp
WP_028471543_Nocardioides_alkalitolerans
WP_038676339_Pimelobacter_simplex
WP_030417717_Streptomyces_sp
WP_037239493_Rhodococcus_wratislaviensis
WP_054188629_Rhodococcus_sp
WP_050655584_Rhodococcus_sp
WP_043662573_Streptomyces_xylophagus
WP_055611366_Streptomyces_phaeochromogenes
WP_019068143_Streptomyces_sp
Photorhabdus_spp
WP_004901645_Acinetobacter_brisouii
WP_004752139_Acinetobacter_sp
EGD06287_Burkholderia_sp
WP_028231793_Burkholderia_mimosarum
WP_025036279_Bradyrhizobium_sp
WP_010360538_Streptomyces_acidiscabies
WP_050371795_Streptomyces_acidiscabies
WP_009308802_Streptomyces_ipomoeae
WP_053740490_Streptomyces_sp
WP_045555454_Streptomyces_sp
WP_012998165_Streptomyces_scabiei
WP_029112541_Mycobacterium_sp
WP_014231725_Vibrio_sp
WP_017523084_Pusillimonas_noertemannii
WP_021471208_Arthrobacter_sp
WP_040794920_Nocardia_higoensis
WP_052223540_Novosphingobium_malaysiense
Bacteria
Fungi
0.2

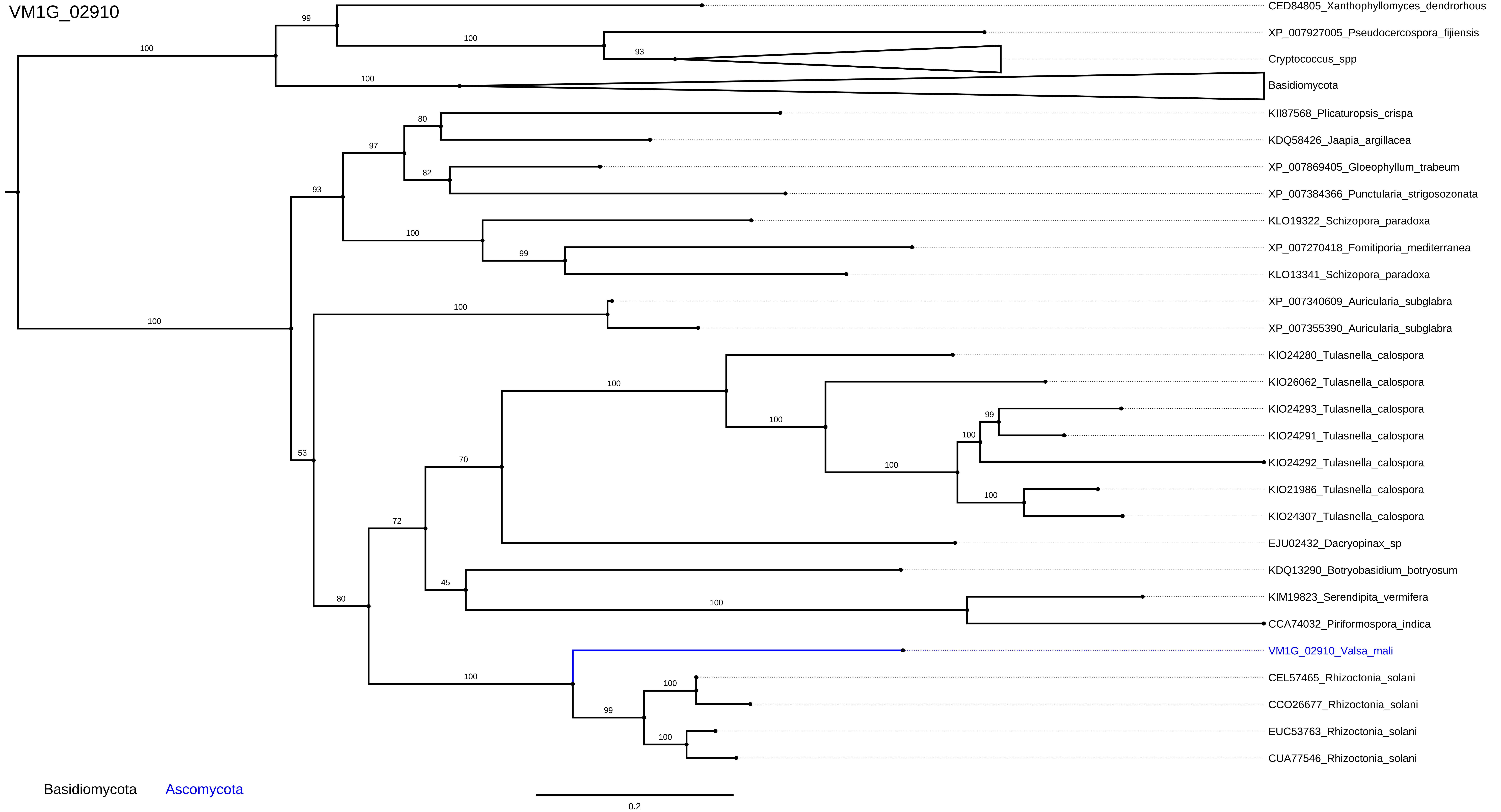
VM1G_02910
CED84805_Xanthophyllomyces_dendrorhous
XP_007927005_Pseudocercospora_fijiensis
Cryptococcus_spp
Basidiomycota
KII87568_Plicaturopsis_crispa
KDQ58426_Jaapia_argillacea
XP_007869405_Gloeophyllum_trabeum
XP_007384366_Punctularia_strigosozonata
KLO19322_Schizopora_paradoxa
XP_007270418_Fomitiporia_mediterranea
KLO13341_Schizopora_paradoxa
XP_007340609_Auricularia_subglabra
XP_007355390_Auricularia_subglabra
KIO24280_Tulasnella_calospora
KIO26062_Tulasnella_calospora
KIO24293_Tulasnella_calospora
KIO24291_Tulasnella_calospora
KIO24292_Tulasnella_calospora
KIO21986_Tulasnella_calospora
KIO24307_Tulasnella_calospora
EJU02432_Dacryopinax_sp
KDQ13290_Botryobasidium_botryosum
KIM19823_Serendipita_vermifera
CCA74032_Piriformospora_indica
VM1G_02910_Valsa_mali
CEL57465_Rhizoctonia_solani
CCO26677_Rhizoctonia_solani
EUC53763_Rhizoctonia_solani
CUA77546_Rhizoctonia_solani
Basidiomycota
Ascomycota
0.2

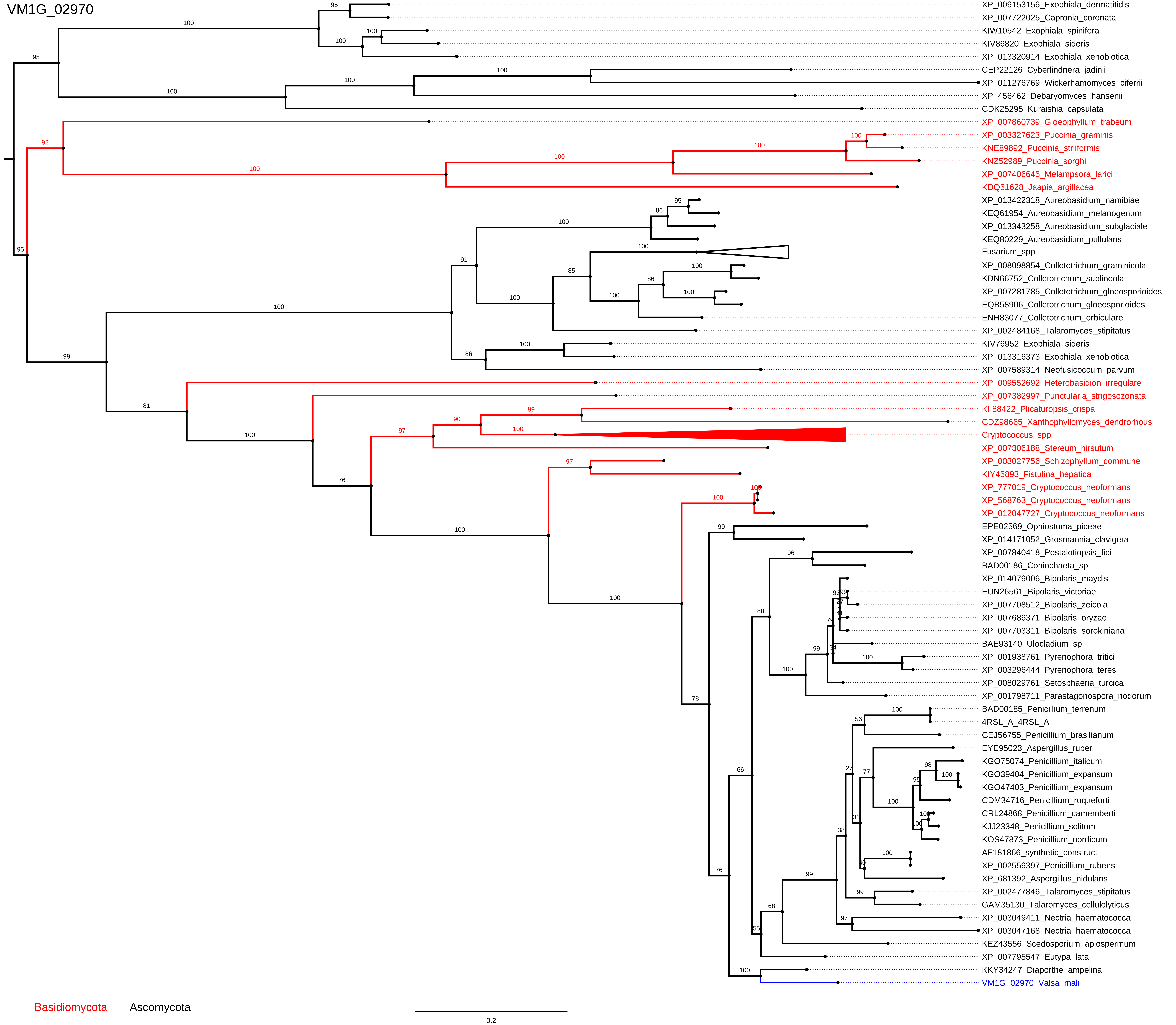
VM1G_02970
XP_009153156_Exophiala_dermatitidis
XP_007722025_Capronia_coronata
KIW10542_Exophiala_spinifera
KIV86820_Exophiala_sideris
XP_013320914_Exophiala_xenobiotica
CEP22126_Cyberlindnera_jadinii
XP_011276769_Wickerhamomyces_ciferrii
XP_456462_Debaryomyces_hansenii
CDK25295_Kuraishia_capsulata
XP_007860739_Gloeophyllum_trabeum
XP_003327623_Puccinia_graminis
KNE89892_Puccinia_striiformis
KNZ52989_Puccinia_sorghi
XP_007406645_Melampsora_larici
KDQ51628_Jaapia_argillacea
XP_013422318_Aureobasidium_namibiae
KEQ61954_Aureobasidium_melanogenum
XP_013343258_Aureobasidium_subglaciale
KEQ80229_Aureobasidium_pullulans
Fusarium_spp
XP_008098854_Colletotrichum_graminicola
KDN66752_Colletotrichum_sublineola
XP_007281785_Colletotrichum_gloeosporioides
EQB58906_Colletotrichum_gloeosporioides
ENH83077_Colletotrichum_orbiculare
XP_002484168_Talaromyces_stipitatus
KIV76952_Exophiala_sideris
XP_013316373_Exophiala_xenobiotica
XP_007589314_Neofusicoccum_parvum
XP_009552692_Heterobasidion_irregulare
XP_007382997_Punctularia_strigosozonata
KII88422_Plicaturopsis_crispa
CDZ98665_Xanthophyllomyces_dendrorhous
Cryptococcus_spp
XP_007306188_Stereum_hirsutum
XP_003027756_Schizophyllum_commune
KIY45893_Fistulina_hepatica
XP_777019_Cryptococcus_neoformans
XP_568763_Cryptococcus_neoformans
XP_012047727_Cryptococcus_neoformans
EPE02569_Ophiostoma_piceae
XP_014171052_Grosmannia_clavigera
XP_007840418_Pestalotiopsis_fici
BAD00186_Coniochaeta_sp
XP_014079006_Bipolaris_maydis
EUN26561_Bipolaris_victoriae
XP_007708512_Bipolaris_zeicola
XP_007686371_Bipolaris_oryzae
XP_007703311_Bipolaris_sorokiniana
BAE93140_Ulocladium_sp
XP_001938761_Pyrenophora_tritici
XP_003296444_Pyrenophora_teres
XP_008029761_Setosphaeria_turcica
XP_001798711_Parastagonospora_nodorum
BAD00185_Penicillium_terrenum
4RSL_A_4RSL_A
CEJ56755_Penicillium_brasilianum
EYE95023_Aspergillus_ruber
KGO75074_Penicillium_italicum
KGO39404_Penicillium_expansum
KGO47403_Penicillium_expansum
CDM34716_Penicillium_roqueforti
CRL24868_Penicillium_camemberti
KJJ23348_Penicillium_solitum
KOS47873_Penicillium_nordicum
AF181866_synthetic_construct
XP_002559397_Penicillium_rubens
XP_681392_Aspergillus_nidulans
XP_002477846_Talaromyces_stipitatus
GAM35130_Talaromyces_cellulolyticus
XP_003049411_Nectria_haematococca
XP_003047168_Nectria_haematococca
KEZ43556_Scedosporium_apiospermum
XP_007795547_Eutypa_lata
KKY34247_Diaporthe_ampelina
VM1G_02970_Valsa_mali
Basidiomycota
Ascomycota
0.2

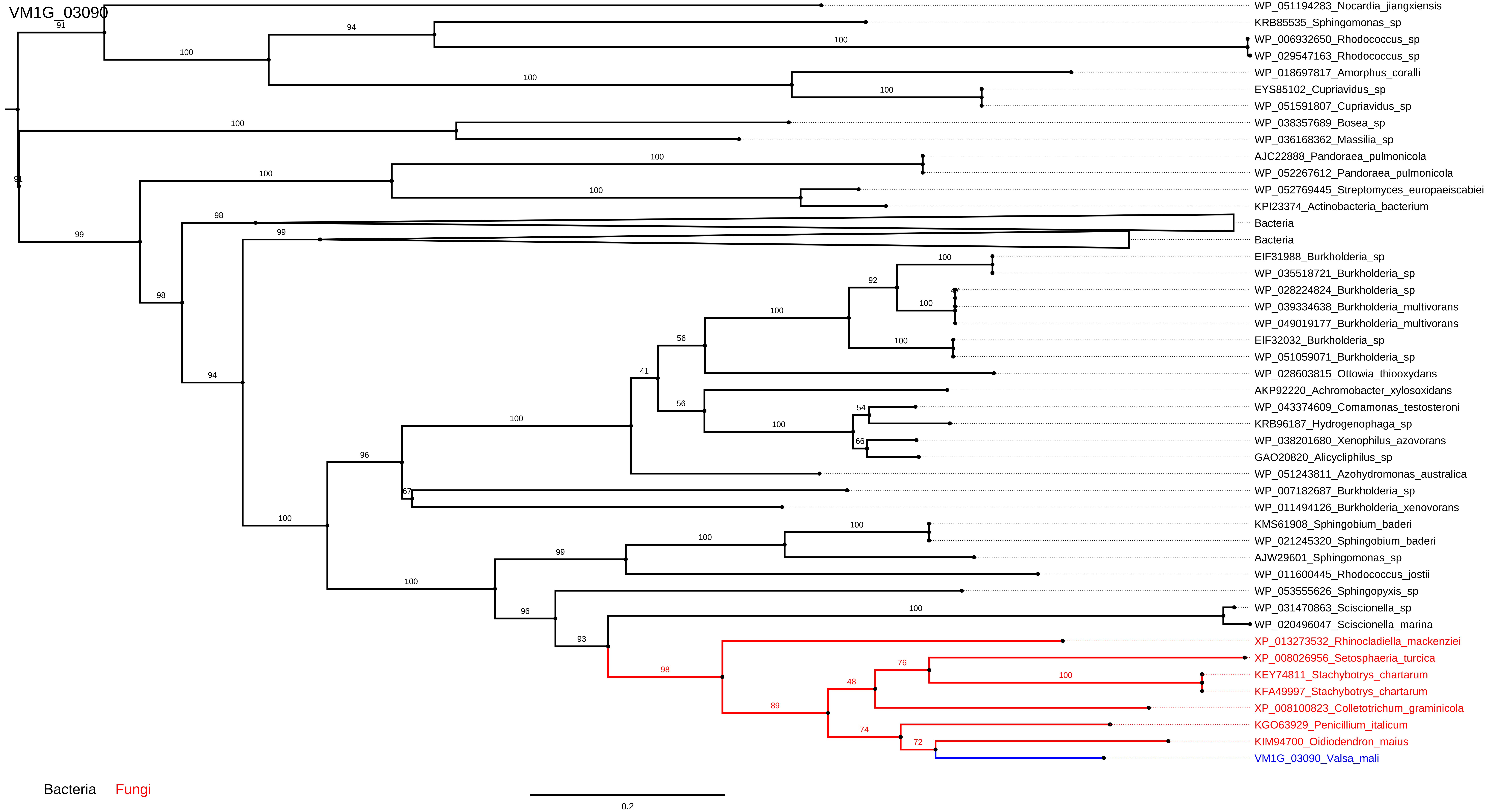
VM1G_03090
WP_051194283_Nocardia_jiangxiensis
KRB85535_Sphingomonas_sp
WP_006932650_Rhodococcus_sp
WP_029547163_Rhodococcus_sp
WP_018697817_Amorphus_coralli
EYS85102_Cupriavidus_sp
WP_051591807_Cupriavidus_sp
WP_038357689_Bosea_sp
WP_036168362_Massilia_sp
AJC22888_Pandoraea_pulmonicola
WP_052267612_Pandoraea_pulmonicola
WP_052769445_Streptomyces_europaeiscabiei
KPI23374_Actinobacteria_bacterium
Bacteria
Bacteria
EIF31988_Burkholderia_sp
WP_035518721_Burkholderia_sp
WP_028224824_Burkholderia_sp
WP_039334638_Burkholderia_multivorans
WP_049019177_Burkholderia_multivorans
EIF32032_Burkholderia_sp
WP_051059071_Burkholderia_sp
WP_028603815_Ottowia_thiooxydans
AKP92220_Achromobacter_xylosoxidans
WP_043374609_Comamonas_testosteroni
KRB96187_Hydrogenophaga_sp
WP_038201680_Xenophilus_azovorans
GAO20820_Alicycliphilus_sp
WP_051243811_Azohydromonas_australica
WP_007182687_Burkholderia_sp
WP_011494126_Burkholderia_xenovorans
KMS61908_Sphingobium_baderi
WP_021245320_Sphingobium_baderi
AJW29601_Sphingomonas_sp
WP_011600445_Rhodococcus_jostii
WP_053555626_Sphingopyxis_sp
WP_031470863_Sciscionella_sp
WP_020496047_Sciscionella_marina
XP_013273532_Rhinocladiella_mackenziei
XP_008026956_Setosphaeria_turcica
KEY74811_Stachybotrys_chartarum
KFA49997_Stachybotrys_chartarum
XP_008100823_Colletotrichum_graminicola
KGO63929_Penicillium_italicum
KIM94700_Oidiodendron_maius
VM1G_03090_Valsa_mali
Bacteria
Fungi
0.2

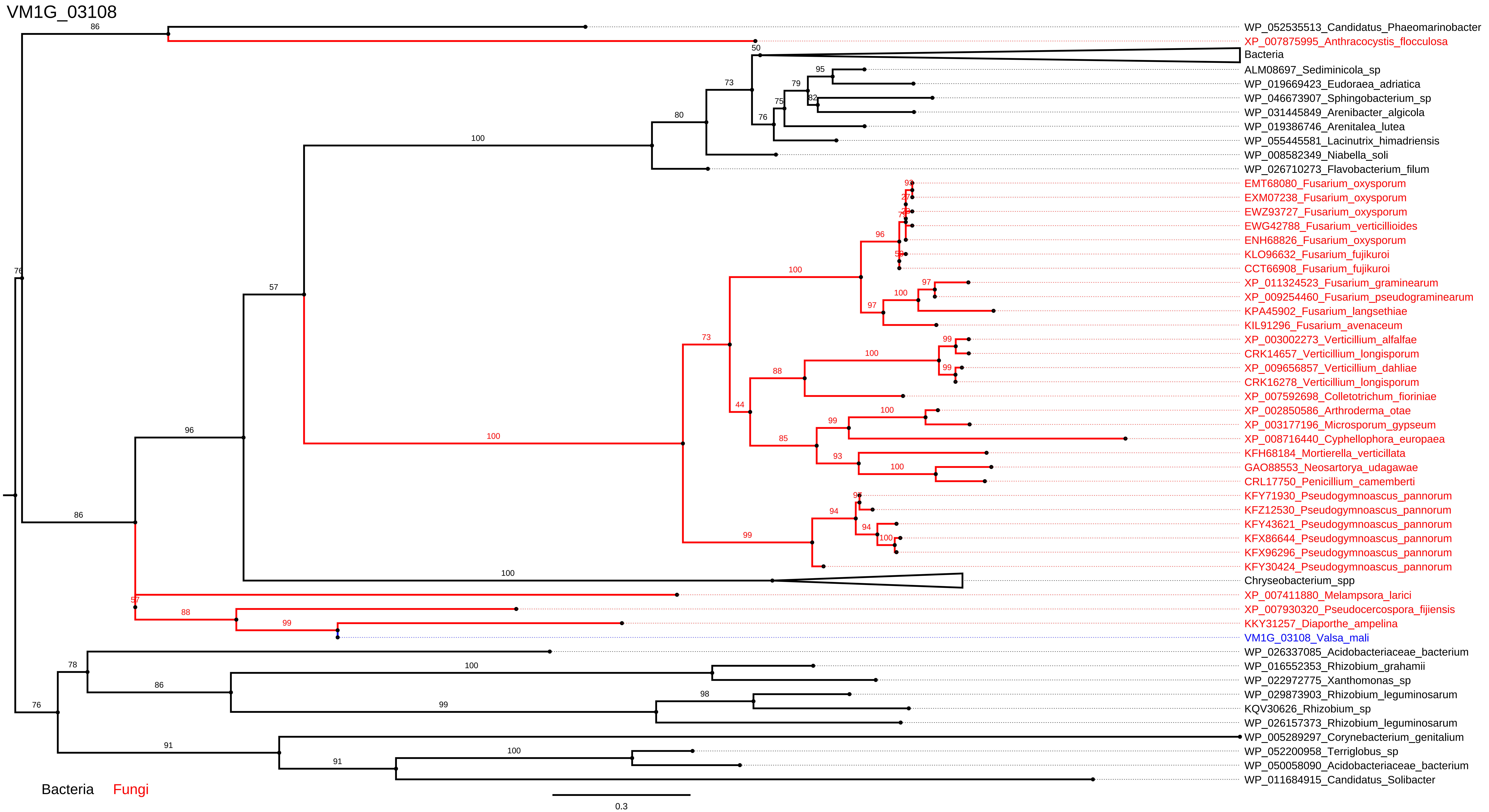

VM1G_03108
WP_052535513_Candidatus_Phaeomarinobacter
XP_007875995_Anthracocystis_flocculosa
Bacteria
ALM08697_Sediminicola_sp
WP_019669423_Eudoraea_adriatica
WP_046673907_Sphingobacterium_sp
WP_031445849_Arenibacter_algicola
WP_019386746_Arenitalea_lutea
WP_055445581_Lacinutrix_himadriensis
WP_008582349_Niabella_soli
WP_026710273_Flavobacterium_filum
EMT68080_Fusarium_oxysporum
EXM07238_Fusarium_oxysporum
EWZ93727_Fusarium_oxysporum
EWG42788_Fusarium_verticillioides
ENH68826_Fusarium_oxysporum
KLO96632_Fusarium_fujikuroi
CCT66908_Fusarium_fujikuroi
XP_011324523_Fusarium_graminearum
XP_009254460_Fusarium_pseudograminearum
KPA45902_Fusarium_langsethiae
KIL91296_Fusarium_avenaceum
XP_003002273_Verticillium_alfalfae
CRK14657_Verticillium_longisporum
XP_009656857_Verticillium_dahliae
CRK16278_Verticillium_longisporum
XP_007592698_Colletotrichum_fioriniae
XP_002850586_Arthroderma_otae
XP_003177196_Microsporum_gypseum
XP_008716440_Cyphellophora_europaea
KFH68184_Mortierella_verticillata
GAO88553_Neosartorya_udagawae
CRL17750_Penicillium_camemberti
KFY71930_Pseudogymnoascus_pannorum
KFZ12530_Pseudogymnoascus_pannorum
KFY43621_Pseudogymnoascus_pannorum
KFX86644_Pseudogymnoascus_pannorum
KFX96296_Pseudogymnoascus_pannorum
KFY30424_Pseudogymnoascus_pannorum
Chryseobacterium_spp
XP_007411880_Melampsora_larici
XP_007930320_Pseudocercospora_fijiensis
KKY31257_Diaporthe_ampelina
VM1G_03108_Valsa_mali
WP_026337085_Acidobacteriaceae_bacterium
WP_016552353_Rhizobium_grahamii
WP_022972775_Xanthomonas_sp
WP_029873903_Rhizobium_leguminosarum
KQV30626_Rhizobium_sp
WP_026157373_Rhizobium_leguminosarum
WP_005289297_Corynebacterium_genitalium
WP_052200958_Terriglobus_sp
WP_050058090_Acidobacteriaceae_bacterium
WP_011684915_Candidatus_Solibacter
Bacteria
Fungi
0.3

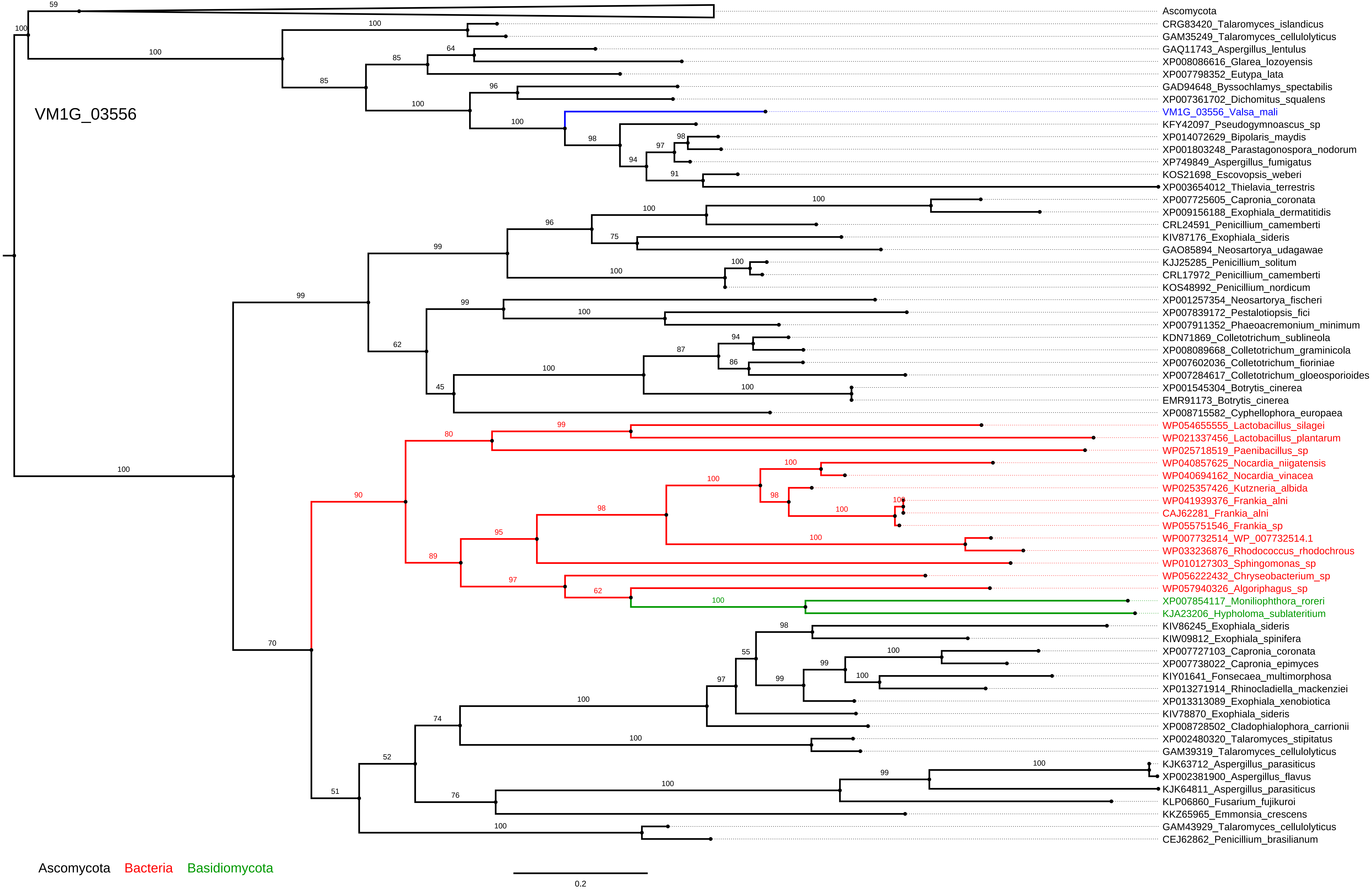
VM1G_03556
Ascomycota
CRG83420_Talaromyces_islandicus
GAM35249_Talaromyces_cellulolyticus
GAQ11743_Aspergillus_lentulus
XP008086616_Glarea_lozoyensis
XP007798352_Eutypa_lata
GAD94648_Byssochlamys_spectabilis
XP007361702_Dichomitus_squalens
VM1G_03556_Valsa_mali
KFY42097_Pseudogymnoascus_sp
XP014072629_Bipolaris_maydis
XP001803248_Parastagonospora_nodorum
XP749849_Aspergillus_fumigatus
KOS21698_Escovopsis_weberi
XP003654012_Thielavia_terrestris
XP007725605_Capronia_coronata
XP009156188_Exophiala_dermatitidis
CRL24591_Penicillium_camemberti
KIV87176_Exophiala_sideris
GAO85894_Neosartorya_udagawae
KJJ25285_Penicillium_solitum
CRL17972_Penicillium_camemberti
KOS48992_Penicillium_nordicum
XP001257354_Neosartorya_fischeri
XP007839172_Pestalotiopsis_fici
XP007911352_Phaeoacremonium_minimum
KDN71869_Colletotrichum_sublineola
XP008089668_Colletotrichum_graminicola
XP007602036_Colletotrichum_fioriniae
XP007284617_Colletotrichum_gloeosporioides
XP001545304_Botrytis_cinerea
EMR91173_Botrytis_cinerea
XP008715582_Cyphellophora_europaea
WP054655555_Lactobacillus_silagei
WP021337456_Lactobacillus_plantarum
WP025718519_Paenibacillus_sp
WP040857625_Nocardia_niigatensis
WP040694162_Nocardia_vinacea
WP025357426_Kutzneria_albida
WP041939376_Frankia_alni
CAJ62281_Frankia_alni
WP055751546_Frankia_sp
WP007732514_WP_007732514.1
WP033236876_Rhodococcus_rhodochrous
WP010127303_Sphingomonas_sp
WP056222432_Chryseobacterium_sp
WP057940326_Algoriphagus_sp
XP007854117_Moniliophthora_roreri
KJA23206_Hypholoma_sublateritium
KIV86245_Exophiala_sideris
KIW09812_Exophiala_spinifera
XP007727103_Capronia_coronata
XP007738022_Capronia_epimyces
KIY01641_Fonsecaea_multimorphosa
XP013271914_Rhinocladiella_mackenziei
XP013313089_Exophiala_xenobiotica
KIV78870_Exophiala_sideris
XP008728502_Cladophialophora_carrionii
XP002480320_Talaromyces_stipitatus
GAM39319_Talaromyces_cellulolyticus
KJK63712_Aspergillus_parasiticus
XP002381900_Aspergillus_flavus
KJK64811_Aspergillus_parasiticus
KLP06860_Fusarium_fujikuroi
KKZ65965_Emmonsia_crescens
GAM43929_Talaromyces_cellulolyticus
CEJ62862_Penicillium_brasilianum
Ascomycota
Bacteria
Basidiomycota
0.2

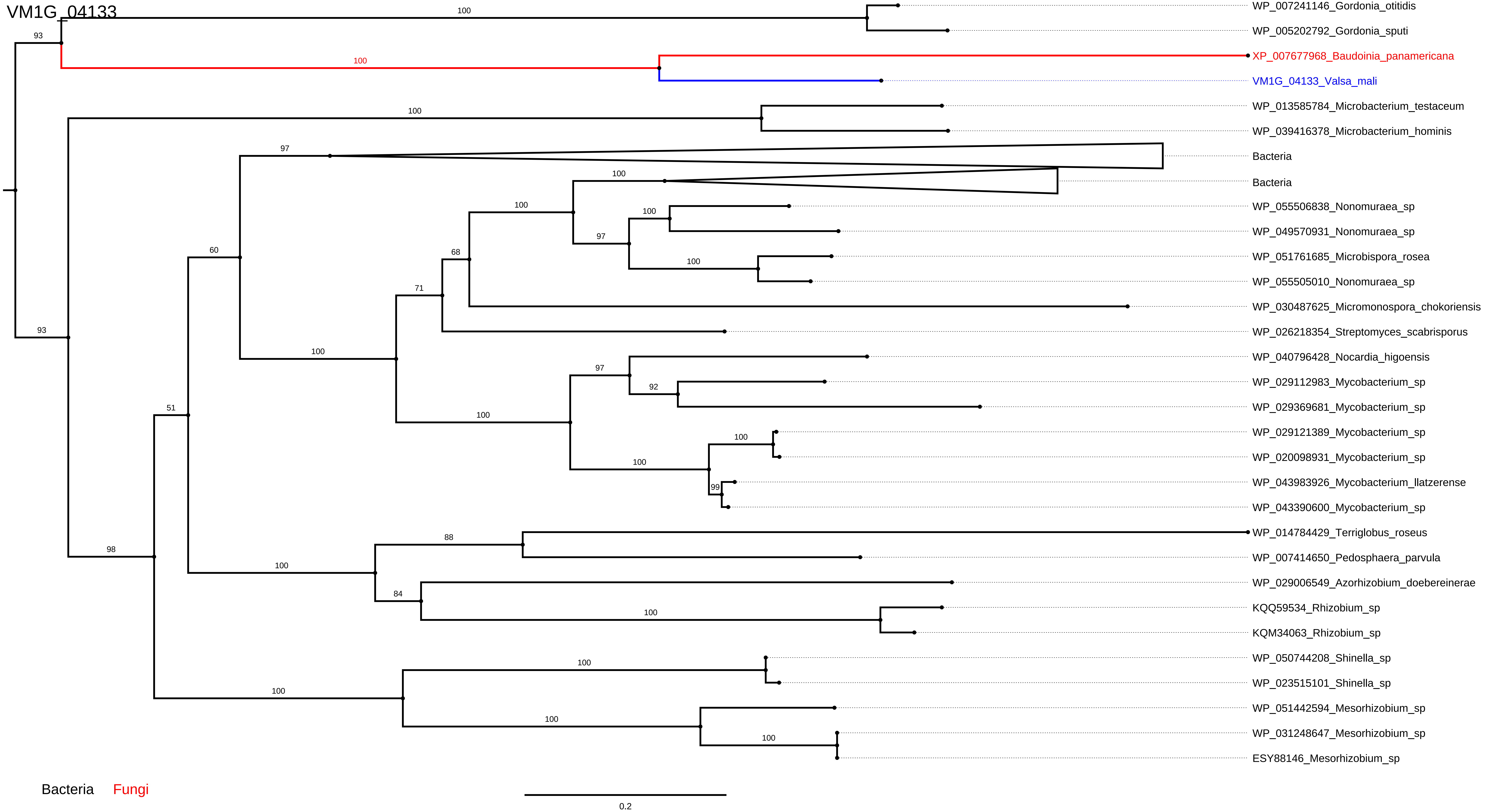
VM1G_04133
WP_007241146_Gordonia_otitidis
WP_005202792_Gordonia_sputi
XP_007677968_Baudoinia_panamericana
VM1G_04133_Valsa_mali
WP_013585784_Microbacterium_testaceum
WP_039416378_Microbacterium_hominis
Bacteria
Bacteria
WP_055506838_Nonomuraea_sp
WP_049570931_Nonomuraea_sp
WP_051761685_Microbispora_rosea
WP_055505010_Nonomuraea_sp
WP_030487625_Micromonospora_chokoriensis
WP_026218354_Streptomyces_scabrisporus
WP_040796428_Nocardia_higoensis
WP_029112983_Mycobacterium_sp
WP_029369681_Mycobacterium_sp
WP_029121389_Mycobacterium_sp
WP_020098931_Mycobacterium_sp
WP_043983926_Mycobacterium_llatzerense
WP_043390600_Mycobacterium_sp
WP_014784429_Terriglobus_roseus
WP_007414650_Pedosphaera_parvula
WP_029006549_Azorhizobium_doebereinerae
KQQ59534_Rhizobium_sp
KQM34063_Rhizobium_sp
WP_050744208_Shinella_sp
WP_023515101_Shinella_sp
WP_051442594_Mesorhizobium_sp
WP_031248647_Mesorhizobium_sp
ESY88146_Mesorhizobium_sp
Bacteria
Fungi
0.2

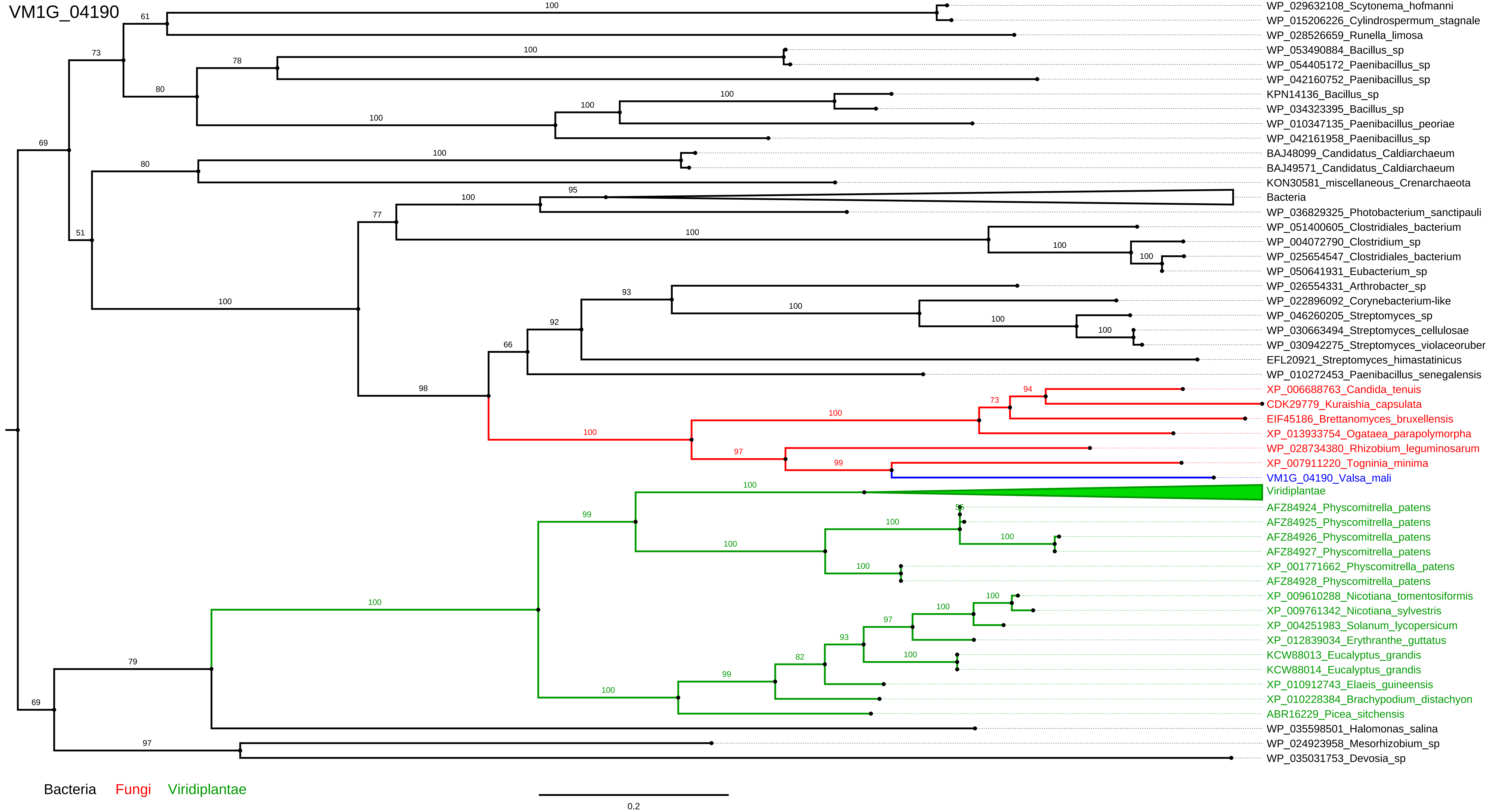
VM1G_04190
WP_029632108_Scytonema_hofmanni
WP_015206226_Cylindrospermum_stagnale
WP_028526659_Runella_limosa
WP_053490884_Bacillus_sp
WP_054405172_Paenibacillus_sp
WP_042160752_Paenibacillus_sp
KPN14136_Bacillus_sp
WP_034323395_Bacillus_sp
WP_010347135_Paenibacillus_peoriae
WP_042161958_Paenibacillus_sp
BAJ48099_Candidatus_Caldiarchaeum
BAJ49571_Candidatus_Caldiarchaeum
KON30581_miscellaneous_Crenarchaeota
Bacteria
WP_036829325_Photobacterium_sanctipauli
WP_051400605_Clostridiales_bacterium
WP_004072790_Clostridium_sp
WP_025654547_Clostridiales_bacterium
WP_050641931_Eubacterium_sp
WP_026554331_Arthrobacter_sp
WP_022896092_Corynebacterium-like
WP_046260205_Streptomyces_sp
WP_030663494_Streptomyces_cellulosae
WP_030942275_Streptomyces_violaceoruber
EFL20921_Streptomyces_himastatinicus
WP_010272453_Paenibacillus_senegalensis
XP_006688763_Candida_tenuis
CDK29779_Kuraishia_capsulata
EIF45186_Brettanomyces_bruxellensis
XP_013933754_Ogataea_parapolymorpha
WP_028734380_Rhizobium_leguminosarum
XP_007911220_Togninia_minima
VM1G_04190_Valsa_mali
Viridiplantae
AFZ84924_Physcomitrella_patens
AFZ84925_Physcomitrella_patens
AFZ84926_Physcomitrella_patens
AFZ84927_Physcomitrella_patens
XP_001771662_Physcomitrella_patens
AFZ84928_Physcomitrella_patens
XP_009610288_Nicotiana_tomentosiformis
XP_009761342_Nicotiana_sylvestris
XP_004251983_Solanum_lycopersicum
XP_012839034_Erythranthe_guttatus
KCW88013_Eucalyptus_grandis
KCW88014_Eucalyptus_grandis
XP_010912743_Elaeis_guineensis
XP_010228384_Brachypodium_distachyon
ABR16229_Picea_sitchensis
WP_035598501_Halomonas_salina
WP_024923958_Mesorhizobium_sp
WP_035031753_Devosia_sp
Bacteria
Fungi
Viridiplantae
0.2

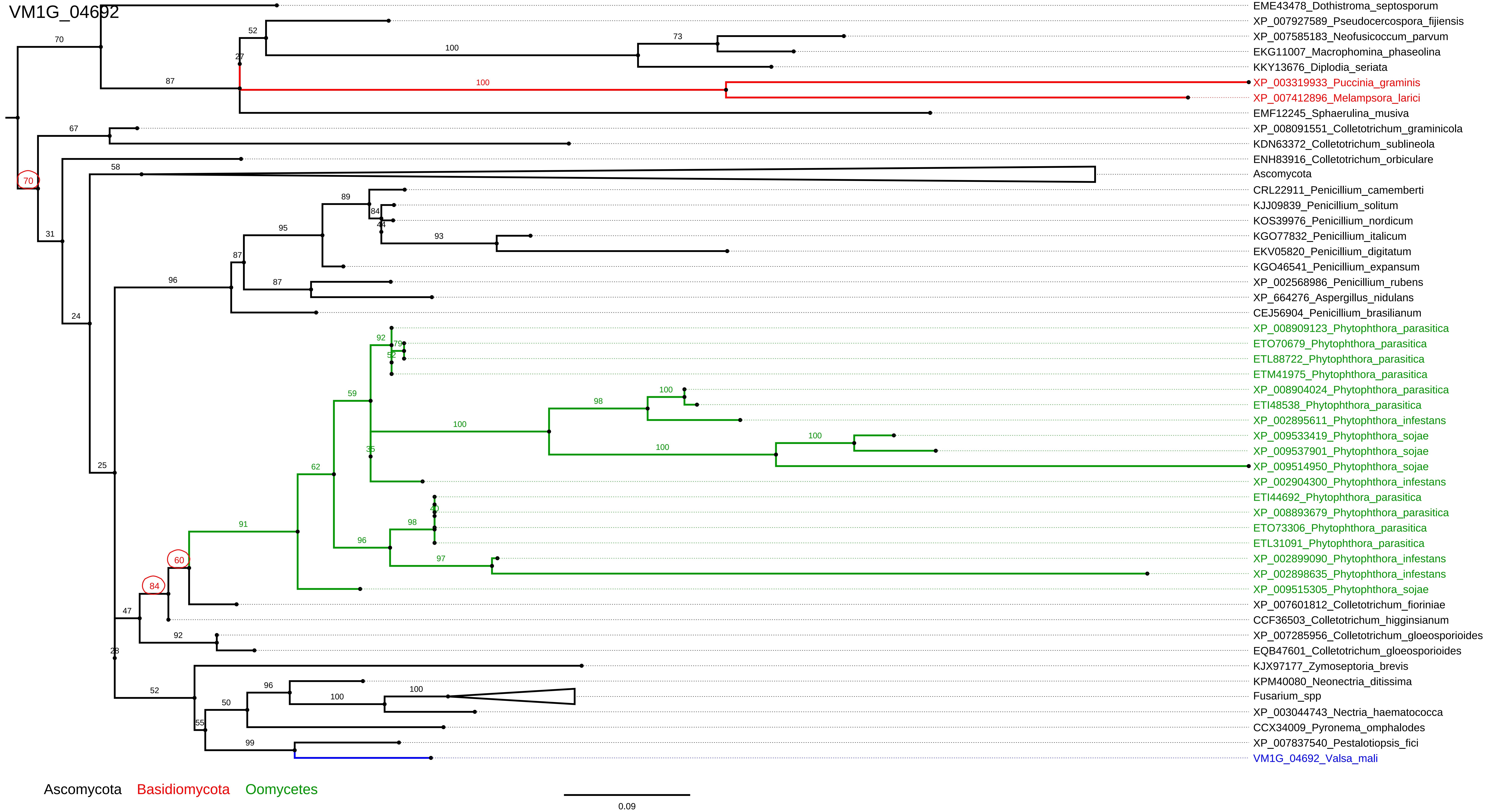
VM1G_04692
EME43478_Dothistroma_septosporum
XP_007927589_Pseudocercospora_fijiensis
XP_007585183_Neofusicoccum_parvum
EKG11007_Macrophomina_phaseolina
KKY13676_Diplodia_seriata
XP_003319933_Puccinia_graminis
XP_007412896_Melampsora_larici
EMF12245_Sphaerulina_musiva
XP_008091551_Colletotrichum_graminicola
KDN63372_Colletotrichum_sublineola
ENH83916_Colletotrichum_orbiculare
Ascomycota
CRL22911_Penicillium_camemberti
KJJ09839_Penicillium_solitum
KOS39976_Penicillium_nordicum
KGO77832_Penicillium_italicum
EKV05820_Penicillium_digitatum
KGO46541_Penicillium_expansum
XP_002568986_Penicillium_rubens
XP_664276_Aspergillus_nidulans
CEJ56904_Penicillium_brasilianum
XP_008909123_Phytophthora_parasitica
ETO70679_Phytophthora_parasitica
ETL88722_Phytophthora_parasitica
ETM41975_Phytophthora_parasitica
XP_008904024_Phytophthora_parasitica
ETI48538_Phytophthora_parasitica
XP_002895611_Phytophthora_infestans
XP_009533419_Phytophthora_sojae
XP_009537901_Phytophthora_sojae
XP_009514950_Phytophthora_sojae
XP_002904300_Phytophthora_infestans
ETI44692_Phytophthora_parasitica
XP_008893679_Phytophthora_parasitica
ETO73306_Phytophthora_parasitica
ETL31091_Phytophthora_parasitica
XP_002899090_Phytophthora_infestans
XP_002898635_Phytophthora_infestans
XP_009515305_Phytophthora_sojae
XP_007601812_Colletotrichum_fioriniae
CCF36503_Colletotrichum_higginsianum
XP_007285956_Colletotrichum_gloeosporioides
EQB47601_Colletotrichum_gloeosporioides
KJX97177_Zymoseptoria_brevis
KPM40080_Neonectria_ditissima
Fusarium_spp
XP_003044743_Nectria_haematococca
CCX34009_Pyronema_omphalodes
XP_007837540_Pestalotiopsis_fici
VM1G_04692_Valsa_mali
Ascomycota
Basidiomycota
Oomycetes
0.09

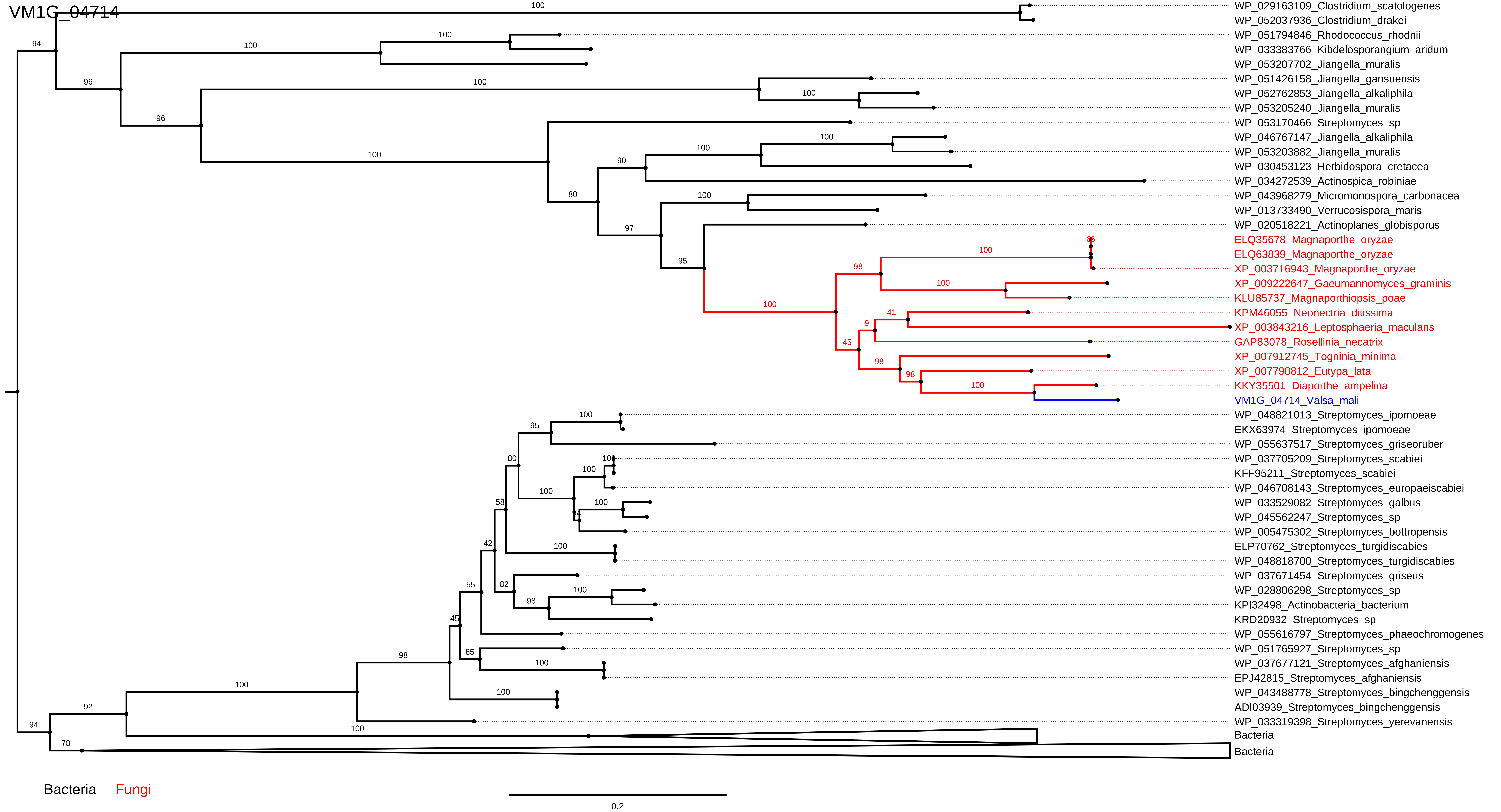
VM1G_04714
WP_029163109_Clostridium_scatologenes
WP_052037936_Clostridium_drakei
WP_051794846_Rhodococcus_rhodnii
WP_033383766_Kibdelosporangium_aridum
WP_053207702_Jiangella_muralis
WP_051426158_Jiangella_gansuensis
WP_052762853_Jiangella_alkaliphila
WP_053205240_Jiangella_muralis
WP_053170466_Streptomyces_sp
WP_046767147_Jiangella_alkaliphila
WP_053203882_Jiangella_muralis
WP_030453123_Herbidospora_cretacea
WP_034272539_Actinospica_robiniae
WP_043968279_Micromonospora_carbonacea
WP_013733490_Verrucosispora_maris
WP_020518221_Actinoplanes_globisporus
ELQ35678_Magnaporthe_oryzae
ELQ63839_Magnaporthe_oryzae
XP_003716943_Magnaporthe_oryzae
XP_009222647_Gaeumannomyces_graminis
KLU85737_Magnaporthiopsis_poae
KPM46055_Neonectria_ditissima
XP_003843216_Leptosphaeria_maculans
GAP83078_Rosellinia_necatrix
XP_007912745_Togninia_minima
XP_007790812_Eutypa_lata
KKY35501_Diaporthe_ampelina
VM1G_04714_Valsa_mali
WP_048821013_Streptomyces_ipomoeae
EKX63974_Streptomyces_ipomoeae
WP_055637517_Streptomyces_griseoruber
WP_037705209_Streptomyces_scabiei
KFF95211_Streptomyces_scabiei
WP_046708143_Streptomyces_europaeiscabiei
WP_033529082_Streptomyces_galbus
WP_045562247_Streptomyces_sp
WP_005475302_Streptomyces_bottropensis
ELP70762_Streptomyces_turgidiscabies
WP_048818700_Streptomyces_turgidiscabies
WP_037671454_Streptomyces_griseus
WP_028806298_Streptomyces_sp
KPI32498_Actinobacteria_bacterium
KRD20932_Streptomyces_sp
WP_055616797_Streptomyces_phaeochromogenes
WP_051765927_Streptomyces_sp
WP_037677121_Streptomyces_afghaniensis
EPJ42815_Streptomyces_afghaniensis
WP_043488778_Streptomyces_bingchenggensis
ADI03939_Streptomyces_bingchenggensis
WP_033319398_Streptomyces_yerevanensis
Bacteria
Bacteria
Bacteria
Fungi
0.2

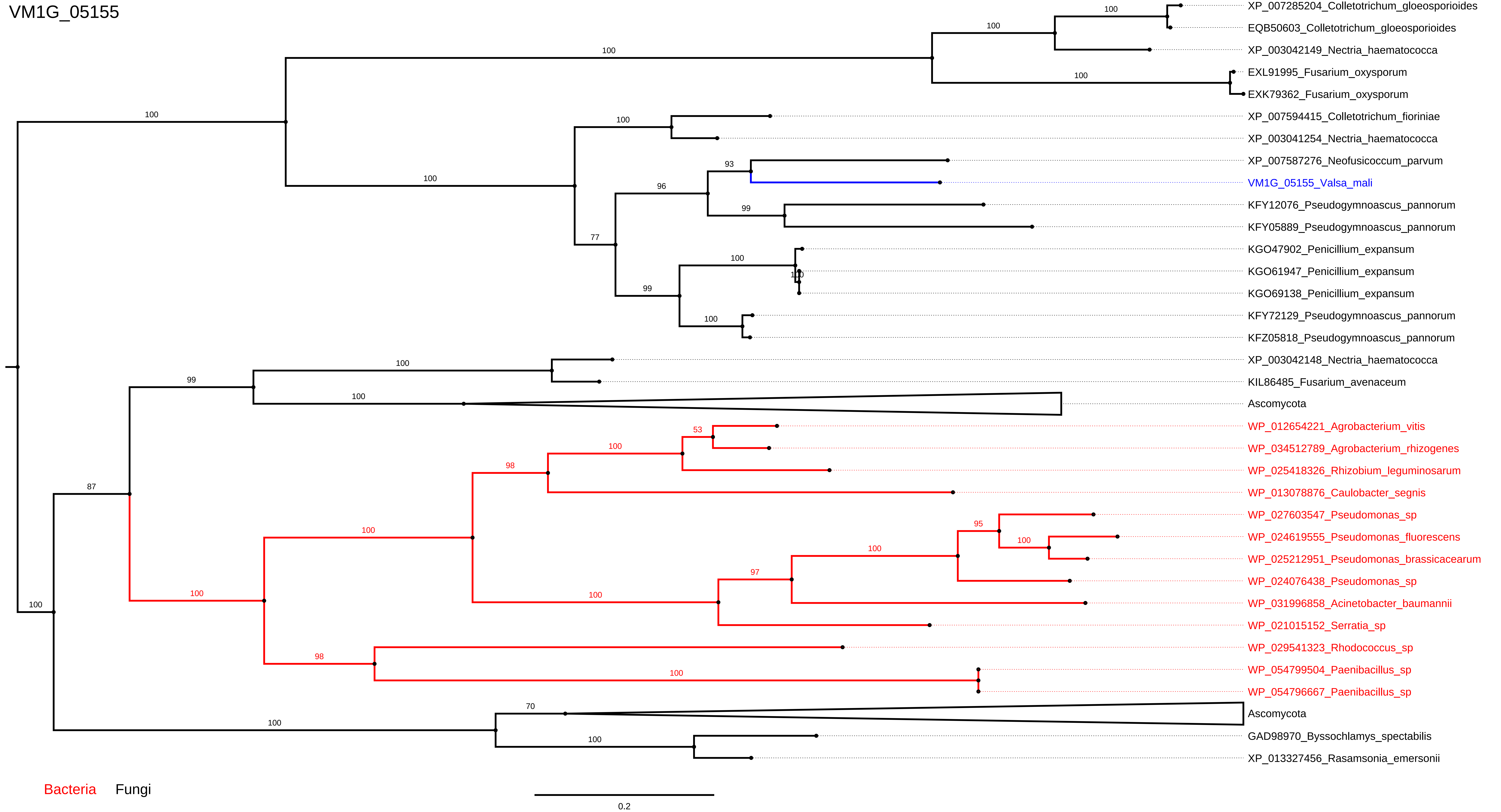
VM1G_05155
XP_007285204_Colletotrichum_gloeosporioides
EQB50603_Colletotrichum_gloeosporioides
XP_003042149_Nectria_haematococca
EXL91995_Fusarium_oxysporum
EXK79362_Fusarium_oxysporum
XP_007594415_Colletotrichum_fioriniae
XP_003041254_Nectria_haematococca
XP_007587276_Neofusicoccum_parvum
VM1G_05155_Valsa_mali
KFY12076_Pseudogymnoascus_pannorum
KFY05889_Pseudogymnoascus_pannorum
KGO47902_Penicillium_expansum
KGO61947_Penicillium_expansum
KGO69138_Penicillium_expansum
KFY72129_Pseudogymnoascus_pannorum
KFZ05818_Pseudogymnoascus_pannorum
XP_003042148_Nectria_haematococca
KIL86485_Fusarium_avenaceum
Ascomycota
WP_012654221_Agrobacterium_vitis
WP_034512789_Agrobacterium_rhizogenes
WP_025418326_Rhizobium_leguminosarum
WP_013078876_Caulobacter_segnis
WP_027603547_Pseudomonas_sp
WP_024619555_Pseudomonas_fluorescens
WP_025212951_Pseudomonas_brassicacearum
WP_024076438_Pseudomonas_sp
WP_031996858_Acinetobacter_baumannii
WP_021015152_Serratia_sp
WP_029541323_Rhodococcus_sp
WP_054799504_Paenibacillus_sp
WP_054796667_Paenibacillus_sp
Ascomycota
GAD98970_Byssochlamys_spectabilis
XP_013327456_Rasamsonia_emersonii
Bacteria
Fungi
0.2

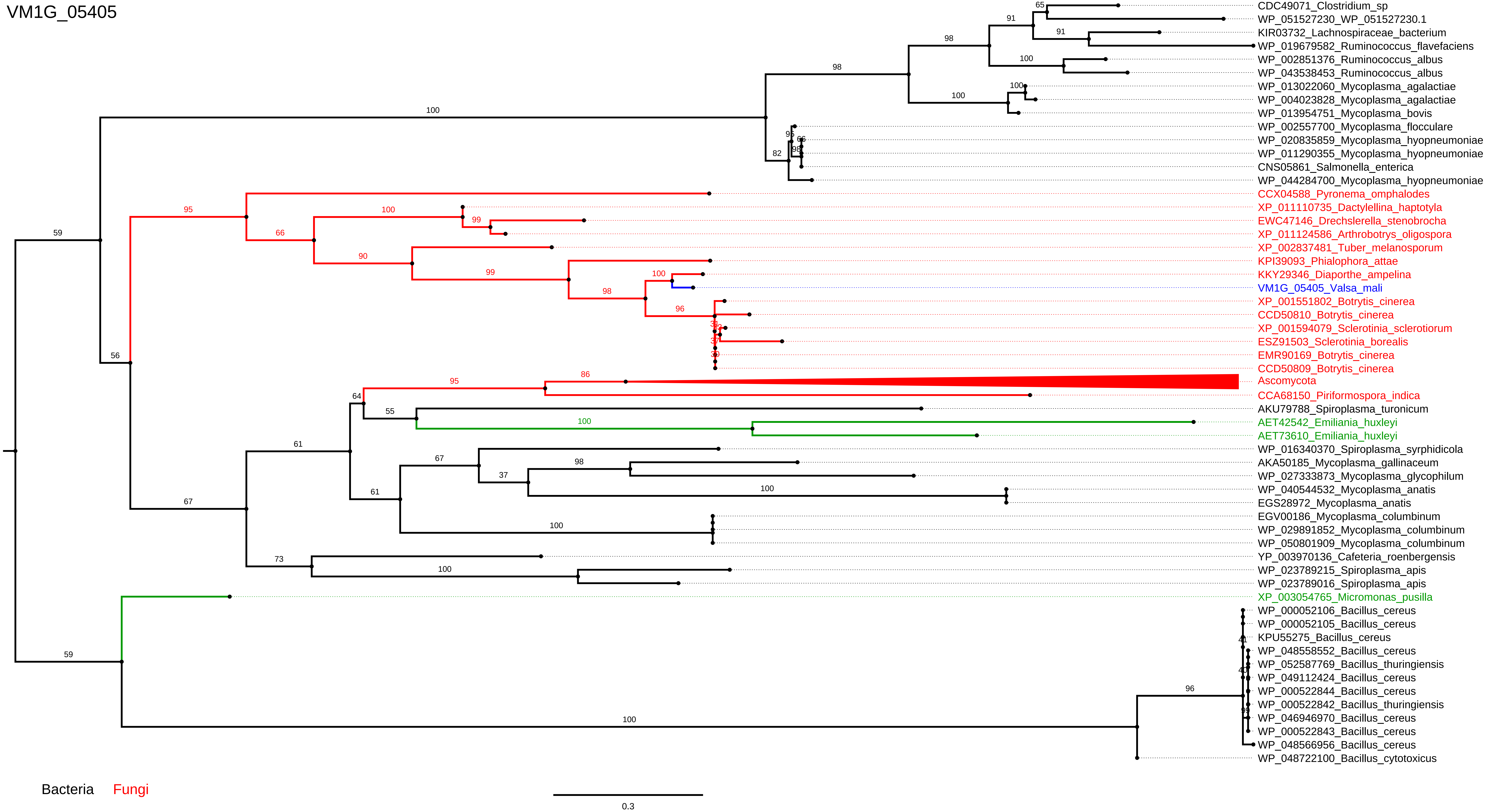
VM1G_05405
CDC49071_Clostridium_sp
WP_051527230_WP_051527230.1
KIR03732_Lachnospiraceae_bacterium
WP_019679582_Ruminococcus_flavefaciens
WP_002851376_Ruminococcus_albus
WP_043538453_Ruminococcus_albus
WP_013022060_Mycoplasma_agalactiae
WP_004023828_Mycoplasma_agalactiae
WP_013954751_Mycoplasma_bovis
WP_002557700_Mycoplasma_flocculare
WP_020835859_Mycoplasma_hyopneumoniae
WP_011290355_Mycoplasma_hyopneumoniae
CNS05861_Salmonella_enterica
WP_044284700_Mycoplasma_hyopneumoniae
CCX04588_Pyronema_omphalodes
XP_011110735_Dactylellina_haptotyla
EWC47146_Drechslerella_stenobrocha
XP_011124586_Arthrobotrys_oligospora
XP_002837481_Tuber_melanosporum
KPI39093_Phialophora_attae
KKY29346_Diaporthe_ampelina
VM1G_05405_Valsa_mali
XP_001551802_Botrytis_cinerea
CCD50810_Botrytis_cinerea
XP_001594079_Sclerotinia_sclerotiorum
ESZ91503_Sclerotinia_borealis
EMR90169_Botrytis_cinerea
CCD50809_Botrytis_cinerea
Ascomycota
CCA68150_Piriformospora_indica
AKU79788_Spiroplasma_turonicum
AET42542_Emiliania_huxleyi
AET73610_Emiliania_huxleyi
WP_016340370_Spiroplasma_syrphidicola
AKA50185_Mycoplasma_gallinaceum
WP_027333873_Mycoplasma_glycophilum
WP_040544532_Mycoplasma_anatis
EGS28972_Mycoplasma_anatis
EGV00186_Mycoplasma_columbinum
WP_029891852_Mycoplasma_columbinum
WP_050801909_Mycoplasma_columbinum
YP_003970136_Cafeteria_roenbergensis
WP_023789215_Spiroplasma_apis
WP_023789016_Spiroplasma_apis
XP_003054765_Micromonas_pusilla
WP_000052106_Bacillus_cereus
WP_000052105_Bacillus_cereus
KPU55275_Bacillus_cereus
WP_048558552_Bacillus_cereus
WP_052587769_Bacillus_thuringiensis
WP_049112424_Bacillus_cereus
WP_000522844_Bacillus_cereus
WP_000522842_Bacillus_thuringiensis
WP_046946970_Bacillus_cereus
WP_000522843_Bacillus_cereus
WP_048566956_Bacillus_cereus
WP_048722100_Bacillus_cytotoxicus
Bacteria
Fungi
0.3

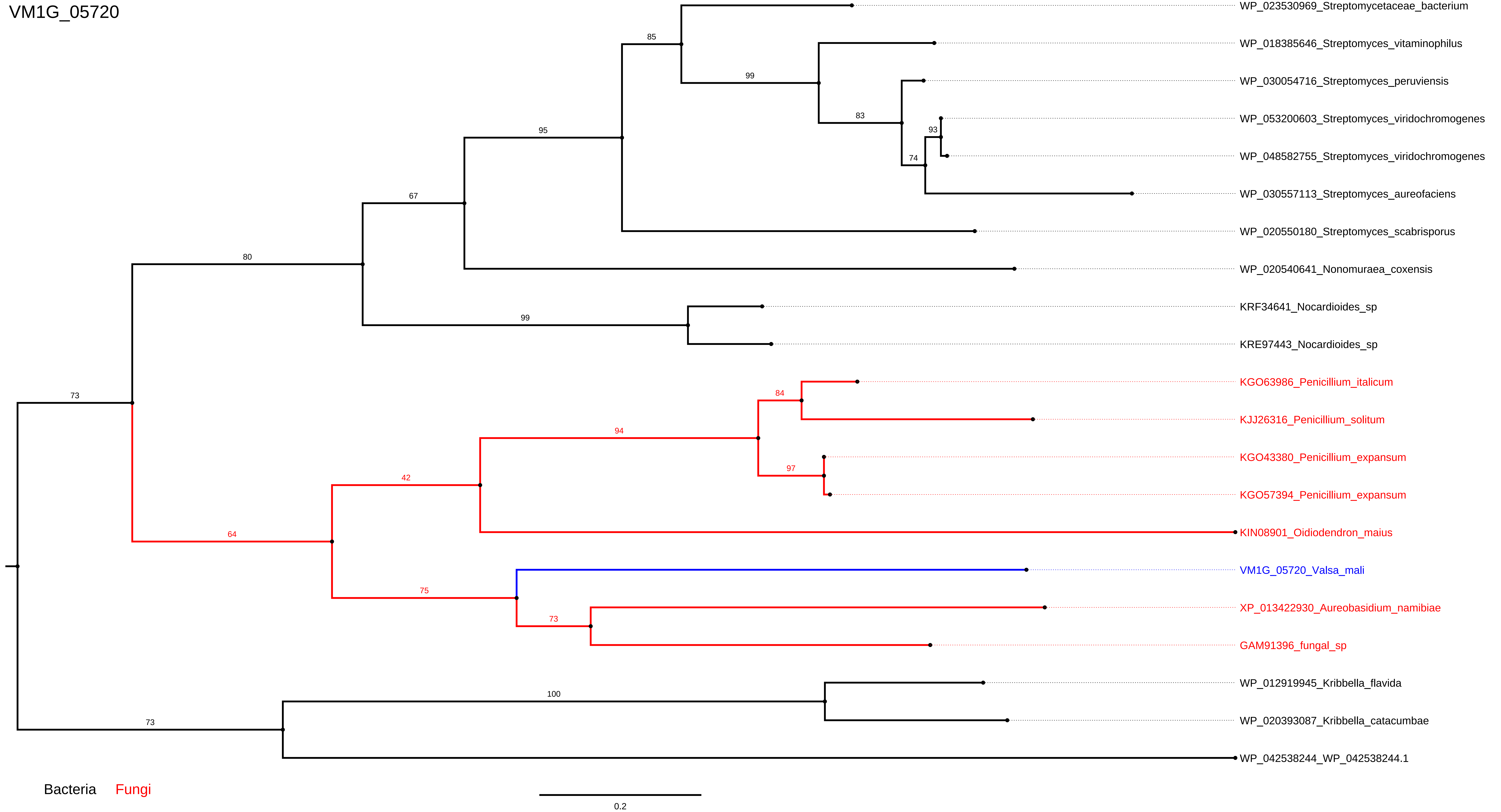
VM1G_05720
WP_023530969_Streptomycetaceae_bacterium
WP_018385646_Streptomyces_vitaminophilus
WP_030054716_Streptomyces_peruviensis
WP_053200603_Streptomyces_viridochromogenes
WP_048582755_Streptomyces_viridochromogenes
WP_030557113_Streptomyces_aureofaciens
WP_020550180_Streptomyces_scabrisporus
WP_020540641_Nonomuraea_coxensis
KRF34641_Nocardioides_sp
KRE97443_Nocardioides_sp
KGO63986_Penicillium_italicum
KJJ26316_Penicillium_solitum
KGO43380_Penicillium_expansum
KGO57394_Penicillium_expansum
KIN08901_Oidiodendron_maius
VM1G_05720_Valsa_mali
XP_013422930_Aureobasidium_namibiae
GAM91396_fungal_sp
WP_012919945_Kribbella_flavida
WP_020393087_Kribbella_catacumbae
WP_042538244_WP_042538244.1
85
99
83
93
74
95
67
80
99
73
84
94
97
42
64
75
73
100
73
Bacteria
Fungi
0.2

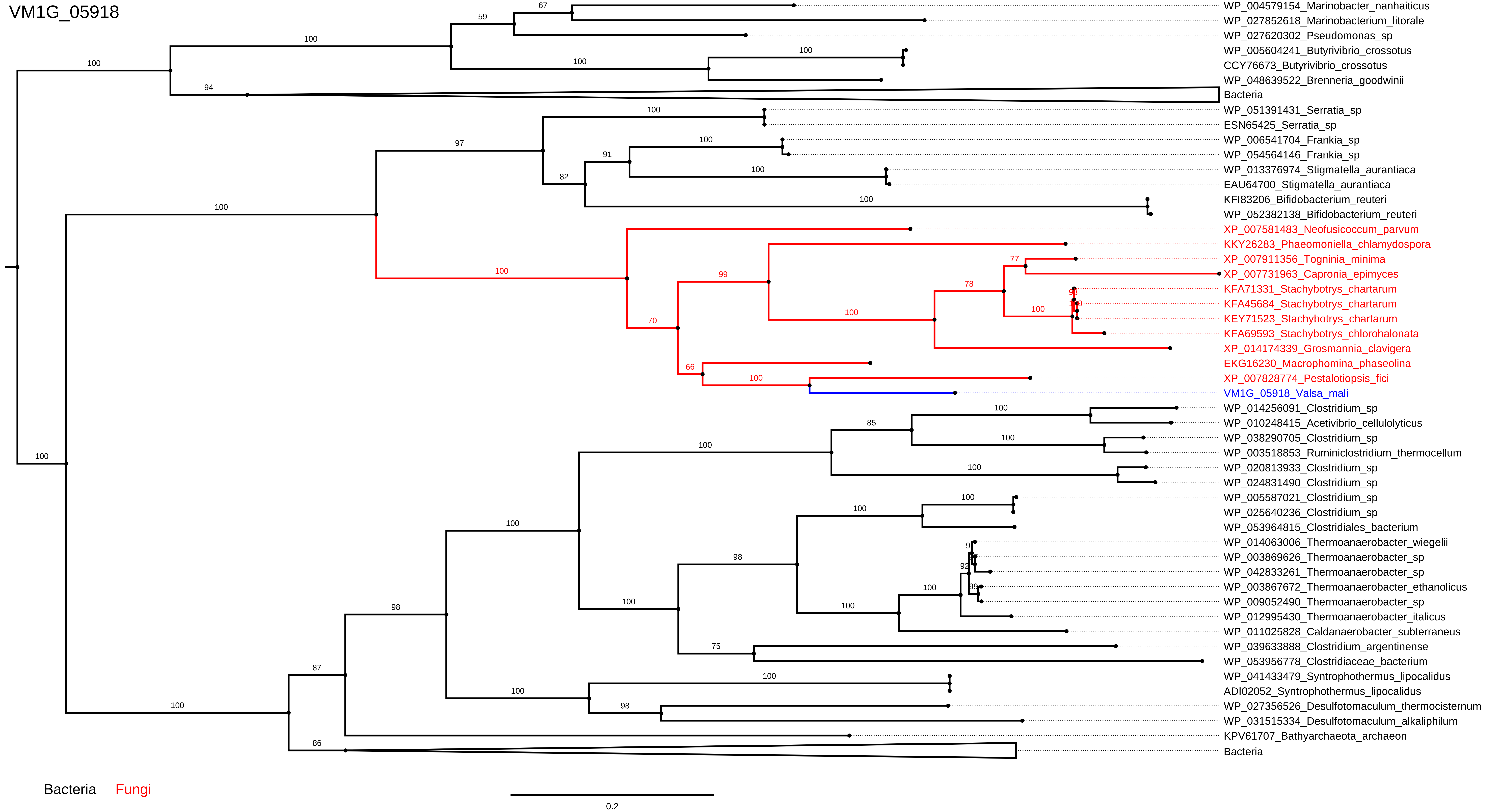

VM1G_05918
WP_004579154_Marinobacter_nanhaiticus
WP_027852618_Marinobacterium_litorale
WP_027620302_Pseudomonas_sp
WP_005604241_Butyrivibrio_crossotus
CCY76673_Butyrivibrio_crossotus
WP_048639522_Brenneria_goodwinii
Bacteria
WP_051391431_Serratia_sp
ESN65425_Serratia_sp
WP_006541704_Frankia_sp
WP_054564146_Frankia_sp
WP_013376974_Stigmatella_aurantiaca
EAU64700_Stigmatella_aurantiaca
KFI83206_Bifidobacterium_reuteri
WP_052382138_Bifidobacterium_reuteri
XP_007581483_Neofusicoccum_parvum
KKY26283_Phaeomoniella_chlamydospora
XP_007911356_Togninia_minima
XP_007731963_Capronia_epimyces
KFA71331_Stachybotrys_chartarum
KFA45684_Stachybotrys_chartarum
KEY71523_Stachybotrys_chartarum
KFA69593_Stachybotrys_chlorohalonata
XP_014174339_Grosmannia_clavigera
EKG16230_Macrophomina_phaseolina
XP_007828774_Pestalotiopsis_fici
VM1G_05918_Valsa_mali
WP_014256091_Clostridium_sp
WP_010248415_Acetivibrio_cellulolyticus
WP_038290705_Clostridium_sp
WP_003518853_Ruminiclostridium_thermocellum
WP_020813933_Clostridium_sp
WP_024831490_Clostridium_sp
WP_005587021_Clostridium_sp
WP_025640236_Clostridium_sp
WP_053964815_Clostridiales_bacterium
WP_014063006_Thermoanaerobacter_wiegelii
WP_003869626_Thermoanaerobacter_sp
WP_042833261_Thermoanaerobacter_sp
WP_003867672_Thermoanaerobacter_ethanolicus
WP_009052490_Thermoanaerobacter_sp
WP_012995430_Thermoanaerobacter_italicus
WP_011025828_Caldanaerobacter_subterraneus
WP_039633888_Clostridium_argentinense
WP_053956778_Clostridiaceae_bacterium
WP_041433479_Syntrophothermus_lipocalidus
ADI02052_Syntrophothermus_lipocalidus
WP_027356526_Desulfotomaculum_thermocisternum
WP_031515334_Desulfotomaculum_alkaliphilum
KPV61707_Bathyarchaeota_archaeon
Bacteria
Bacteria
Fungi
0.2

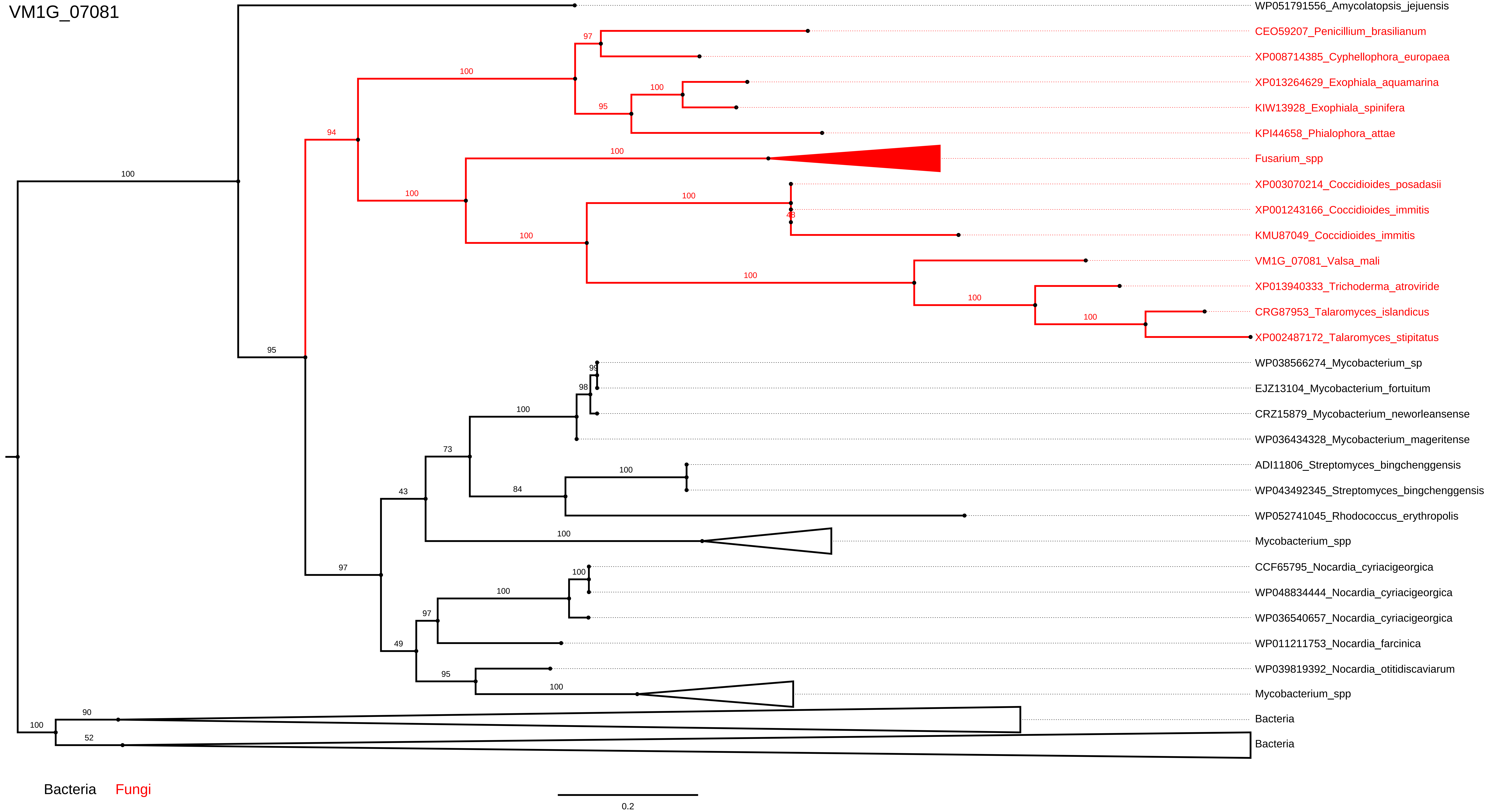
VM1G_07081
WP051791556_Amycolatopsis_jejuensis
CEO59207_Penicillium_brasilianum
XP008714385_Cyphellophora_europaea
XP013264629_Exophiala_aquamarina
KIW13928_Exophiala_spinifera
KPI44658_Phialophora_attae
Fusarium_spp
XP003070214_Coccidioides_posadasii
XP001243166_Coccidioides_immitis
KMU87049_Coccidioides_immitis
VM1G_07081_Valsa_mali
XP013940333_Trichoderma_atroviride
CRG87953_Talaromyces_islandicus
XP002487172_Talaromyces_stipitatus
WP038566274_Mycobacterium_sp
EJZ13104_Mycobacterium_fortuitum
CRZ15879_Mycobacterium_neworleansense
WP036434328_Mycobacterium_mageritense
ADI11806_Streptomyces_bingchenggensis
WP043492345_Streptomyces_bingchenggensis
WP052741045_Rhodococcus_erythropolis
Mycobacterium_spp
CCF65795_Nocardia_cyriacigeorgica
WP048834444_Nocardia_cyriacigeorgica
WP036540657_Nocardia_cyriacigeorgica
WP011211753_Nocardia_farcinica
WP039819392_Nocardia_otitidiscaviarum
Mycobacterium_spp
Bacteria
Bacteria
Bacteria Fungi
0.2

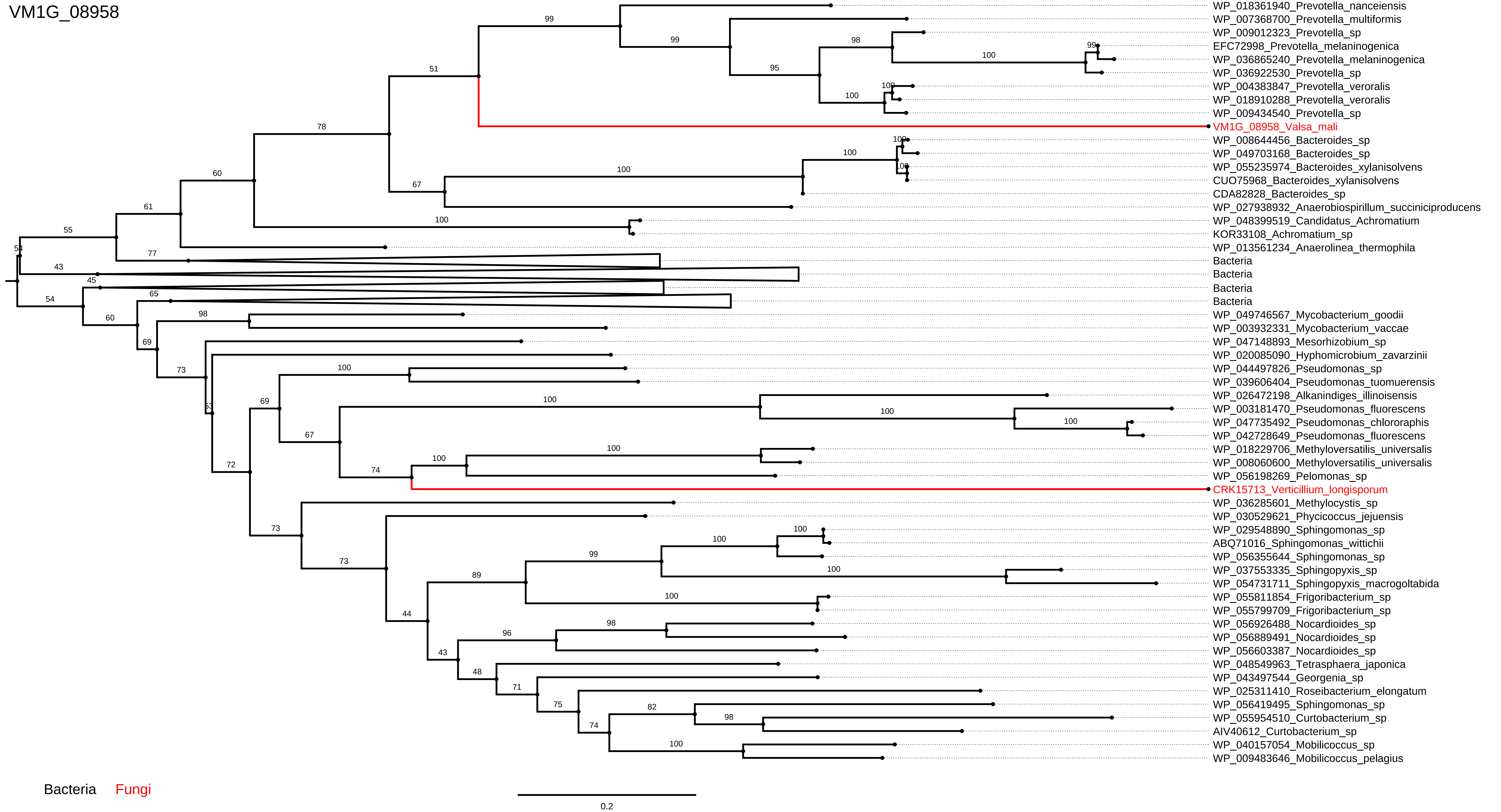
VM1G_08958
WP_018361940_Prevotella_nanceiensis
WP_007368700_Prevotella_multiformis
WP_009012323_Prevotella_sp
EFC72998_Prevotella_melaninogenica
WP_036865240_Prevotella_melaninogenica
WP_036922530_Prevotella_sp
WP_004383847_Prevotella_veroralis
WP_018910288_Prevotella_veroralis
WP_009434540_Prevotella_sp
VM1G_08958_Valsa_mali
WP_008644456_Bacteroides_sp
WP_049703168_Bacteroides_sp
WP_055235974_Bacteroides_xylanisolvens
CUO75968_Bacteroides_xylanisolvens
CDA82828_Bacteroides_sp
WP_027938932_Anaerobiospirillum_succiniciproducens
WP_048399519_Candidatus_Achromatium
KOR33108_Achromatium_sp
WP_013561234_Anaerolinea_thermophila
Bacteria
Bacteria
Bacteria
Bacteria
WP_049746567_Mycobacterium_goodii
WP_003932331_Mycobacterium_vaccae
WP_047148893_Mesorhizobium_sp
WP_020085090_Hyphomicrobium_zavarzinii
WP_044497826_Pseudomonas_sp
WP_039606404_Pseudomonas_tuomuerensis
WP_026472198_Alkanindiges_illinoisensis
WP_003181470_Pseudomonas_fluorescens
WP_047735492_Pseudomonas_chlororaphis
WP_042728649_Pseudomonas_fluorescens
WP_018229706_Methyloversatilis_universalis
WP_008060600_Methyloversatilis_universalis
WP_056198269_Pelomonas_sp
CRK15713_Verticillium_longisporum
WP_036285601_Methylocystis_sp
WP_030529621_Phycicoccus_jejuensis
WP_029548890_Sphingomonas_sp
ABQ71016_Sphingomonas_wittichii
WP_056355644_Sphingomonas_sp
WP_037553335_Sphingopyxis_sp
WP_054731711_Sphingopyxis_macrogoltabida
WP_055811854_Frigoribacterium_sp
WP_055799709_Frigoribacterium_sp
WP_056926488_Nocardioides_sp
WP_056889491_Nocardioides_sp
WP_056603387_Nocardioides_sp
WP_048549963_Tetrasphaera_japonica
WP_043497544_Georgenia_sp
WP_025311410_Roseibacterium_elongatum
WP_056419495_Sphingomonas_sp
WP_055954510_Curtobacterium_sp
AIV40612_Curtobacterium_sp
WP_040157054_Mobilicoccus_sp
WP_009483646_Mobilicoccus_pelagius
Bacteria
Fungi
0.2

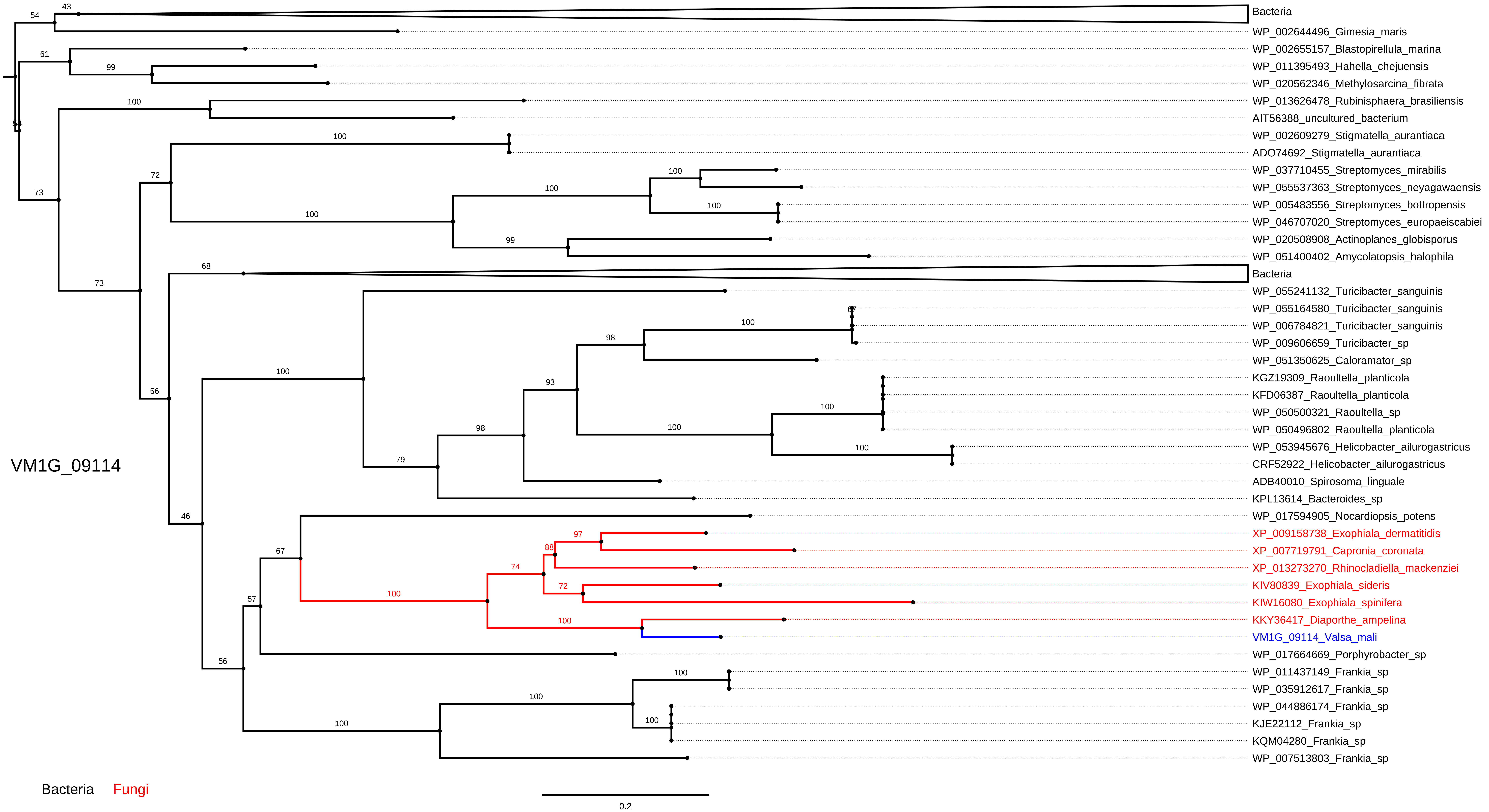
VM1G_09114
Bacteria
WP_002644496_Gimesia_maris
WP_002655157_Blastopirellula_marina
WP_011395493_Hahella_chejuensis
WP_020562346_Methylosarcina_fibrata
WP_013626478_Rubinisphaera_brasiliensis
AIT56388_uncultured_bacterium
WP_002609279_Stigmatella_aurantiaca
ADO74692_Stigmatella_aurantiaca
WP_037710455_Streptomyces_mirabilis
WP_055537363_Streptomyces_neyagawaensis
WP_005483556_Streptomyces_bottropensis
WP_046707020_Streptomyces_europaeiscabiei
WP_020508908_Actinoplanes_globisporus
WP_051400402_Amycolatopsis_halophila
Bacteria
WP_055241132_Turicibacter_sanguinis
WP_055164580_Turicibacter_sanguinis
WP_006784821_Turicibacter_sanguinis
WP_009606659_Turicibacter_sp
WP_051350625_Caloramator_sp
KGZ19309_Raoultella_planticola
KFD06387_Raoultella_planticola
WP_050500321_Raoultella_sp
WP_050496802_Raoultella_planticola
WP_053945676_Helicobacter_ailurogastricus
CRF52922_Helicobacter_ailurogastricus
ADB40010_Spirosoma_linguale
KPL13614_Bacteroides_sp
WP_017594905_Nocardiopsis_potens
XP_009158738_Exophiala_dermatitidis
XP_007719791_Capronia_coronata
XP_013273270_Rhinocladiella_mackenziei
KIV80839_Exophiala_sideris
KIW16080_Exophiala_spinifera
KKY36417_Diaporthe_ampelina
VM1G_09114_Valsa_mali
WP_017664669_Porphyrobacter_sp
WP_011437149_Frankia_sp
WP_035912617_Frankia_sp
WP_044886174_Frankia_sp
KJE22112_Frankia_sp
KQM04280_Frankia_sp
WP_007513803_Frankia_sp
Bacteria Fungi
0.2

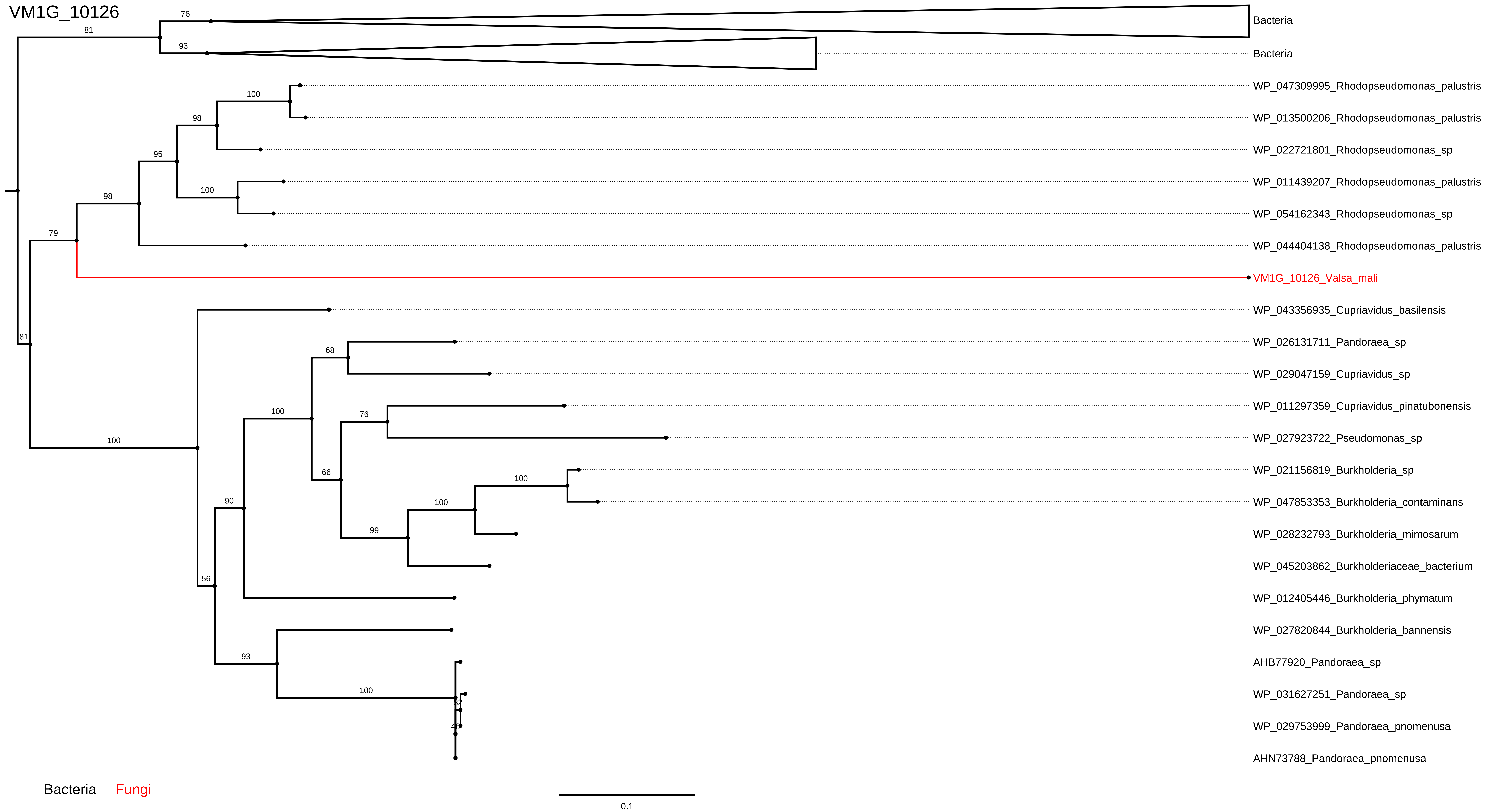
VM1G_10126
Bacteria
Bacteria
WP_047309995_Rhodopseudomonas_palustris
WP_013500206_Rhodopseudomonas_palustris
WP_022721801_Rhodopseudomonas_sp
WP_011439207_Rhodopseudomonas_palustris
WP_054162343_Rhodopseudomonas_sp
WP_044404138_Rhodopseudomonas_palustris
VM1G_10126_Valsa_mali
WP_043356935_Cupriavidus_basilensis
WP_026131711_Pandoraea_sp
WP_029047159_Cupriavidus_sp
WP_011297359_Cupriavidus_pinatubonensis
WP_027923722_Pseudomonas_sp
WP_021156819_Burkholderia_sp
WP_047853353_Burkholderia_contaminans
WP_028232793_Burkholderia_mimosarum
WP_045203862_Burkholderiaceae_bacterium
WP_012405446_Burkholderia_phymatum
WP_027820844_Burkholderia_bannensis
AHB77920_Pandoraea_sp
WP_031627251_Pandoraea_sp
WP_029753999_Pandoraea_pnomenusa
AHN73788_Pandoraea_pnomenusa
Bacteria
Fungi
0.1

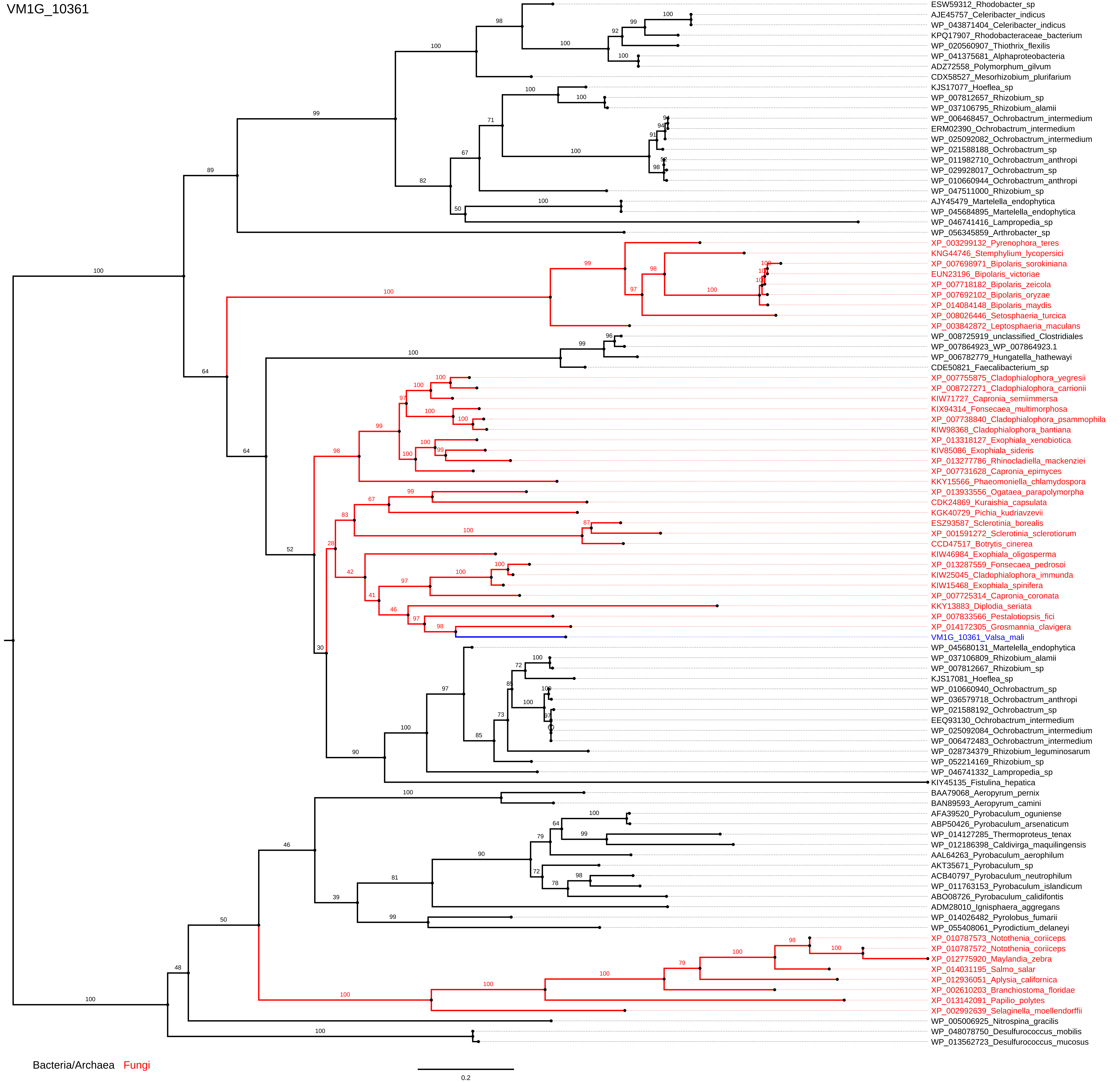
VM1G_10361
ESW59312_Rhodobacter_sp
AJE45757_Celeribacter_indicus
WP_043871404_Celeribacter_indicus
KPQ17907_Rhodobacteraceae_bacterium
WP_020560907_Thiothrix_flexilis
WP_041375681_Alphaproteobacteria
ADZ72558_Polymorphum_gilvum
CDX58527_Mesorhizobium_plurifarium
KJS17077_Hoeflea_sp
WP_007812657_Rhizobium_sp
WP_037106795_Rhizobium_alamii
WP_006468457_Ochrobactrum_intermedium
ERM02390_Ochrobactrum_intermedium
WP_025092082_Ochrobactrum_intermedium
WP_021588188_Ochrobactrum_sp
WP_011982710_Ochrobactrum_anthropi
WP_029928017_Ochrobactrum_sp
WP_010660944_Ochrobactrum_anthropi
WP_047511000_Rhizobium_sp
AJY45479_Martelella_endophytica
WP_045684895_Martelella_endophytica
WP_046741416_Lampropedia_sp
WP_056345859_Arthrobacter_sp
XP_003299132_Pyrenophora_teres
KNG44746_Stemphylium_lycopersici
XP_007698971_Bipolaris_sorokiniana
EUN23196_Bipolaris_victoriae
XP_007718182_Bipolaris_zeicola
XP_007692102_Bipolaris_oryzae
XP_014084148_Bipolaris_maydis
XP_008026446_Setosphaeria_turcica
XP_003842872_Leptosphaeria_maculans
WP_008725919_unclassified_Clostridiales
WP_007864923_WP_007864923.1
WP_006782779_Hungatella_hathewayi
CDE50821_Faecalibacterium_sp
XP_007755875_Cladophialophora_yegresii
XP_008727271_Cladophialophora_carrionii
KIW71727_Capronia_semiimmersa
KIX94314_Fonsecaea_multimorphosa
XP_007738840_Cladophialophora_psammophila
KIW98368_Cladophialophora_bantiana
XP_013318127_Exophiala_xenobiotica
KIV85086_Exophiala_sideris
XP_013277786_Rhinocladiella_mackenziei
XP_007731628_Capronia_epimyces
KKY15566_Phaeomoniella_chlamydospora
XP_013933556_Ogataea_parapolymorpha
CDK24869_Kuraishia_capsulata
KGK40729_Pichia_kudriavzevii
ESZ93587_Sclerotinia_borealis
XP_001591272_Sclerotinia_sclerotiorum
CCD47517_Botrytis_cinerea
KIW46984_Exophiala_oligosperma
XP_013287559_Fonsecaea_pedrosoi
KIW25045_Cladophialophora_immunda
KIW15468_Exophiala_spinifera
XP_007725314_Capronia_coronata
KKY13883_Diplodia_seriata
XP_007833566_Pestalotiopsis_fici
XP_014172305_Grosmannia_clavigera
VM1G_10361_Valsa_mali
WP_045680131_Martelella_endophytica
WP_037106809_Rhizobium_alamii
WP_007812667_Rhizobium_sp
KJS17081_Hoeflea_sp
WP_010660940_Ochrobactrum_sp
WP_036579718_Ochrobactrum_anthropi
WP_021588192_Ochrobactrum_sp
EEQ93130_Ochrobactrum_intermedium
WP_025092084_Ochrobactrum_intermedium
WP_006472483_Ochrobactrum_intermedium
WP_028734379_Rhizobium_leguminosarum
WP_052214169_Rhizobium_sp
WP_046741332_Lampropedia_sp
KIY45135_Fistulina_hepatica
BAA79068_Aeropyrum_pernix
BAN89593_Aeropyrum_camini
AFA39520_Pyrobaculum_oguniense
ABP50426_Pyrobaculum_arsenaticum
WP_014127285_Thermoproteus_tenax
WP_012186398_Caldivirga_maquilingensis
AAL64263_Pyrobaculum_aerophilum
AKT35671_Pyrobaculum_sp
ACB40797_Pyrobaculum_neutrophilum
WP_011763153_Pyrobaculum_islandicum
ABO08726_Pyrobaculum_calidifontis
ADM28010_Ignisphaera_aggregans
WP_014026482_Pyrolobus_fumarii
WP_055408061_Pyrodictium_delaneyi
XP_010787573_Notothenia_coriiceps
XP_010787572_Notothenia_coriiceps
XP_012775920_Maylandia_zebra
XP_014031195_Salmo_salar
XP_012936051_Aplysia_californica
XP_002610203_Branchiostoma_floridae
XP_013142091_Papilio_polytes
XP_002992639_Selaginella_moellendorffii
WP_005006925_Nitrospina_gracilis
WP_048078750_Desulfurococcus_mobilis
WP_013562723_Desulfurococcus_mucosus
Bacteria/Archaea Fungi
0.2

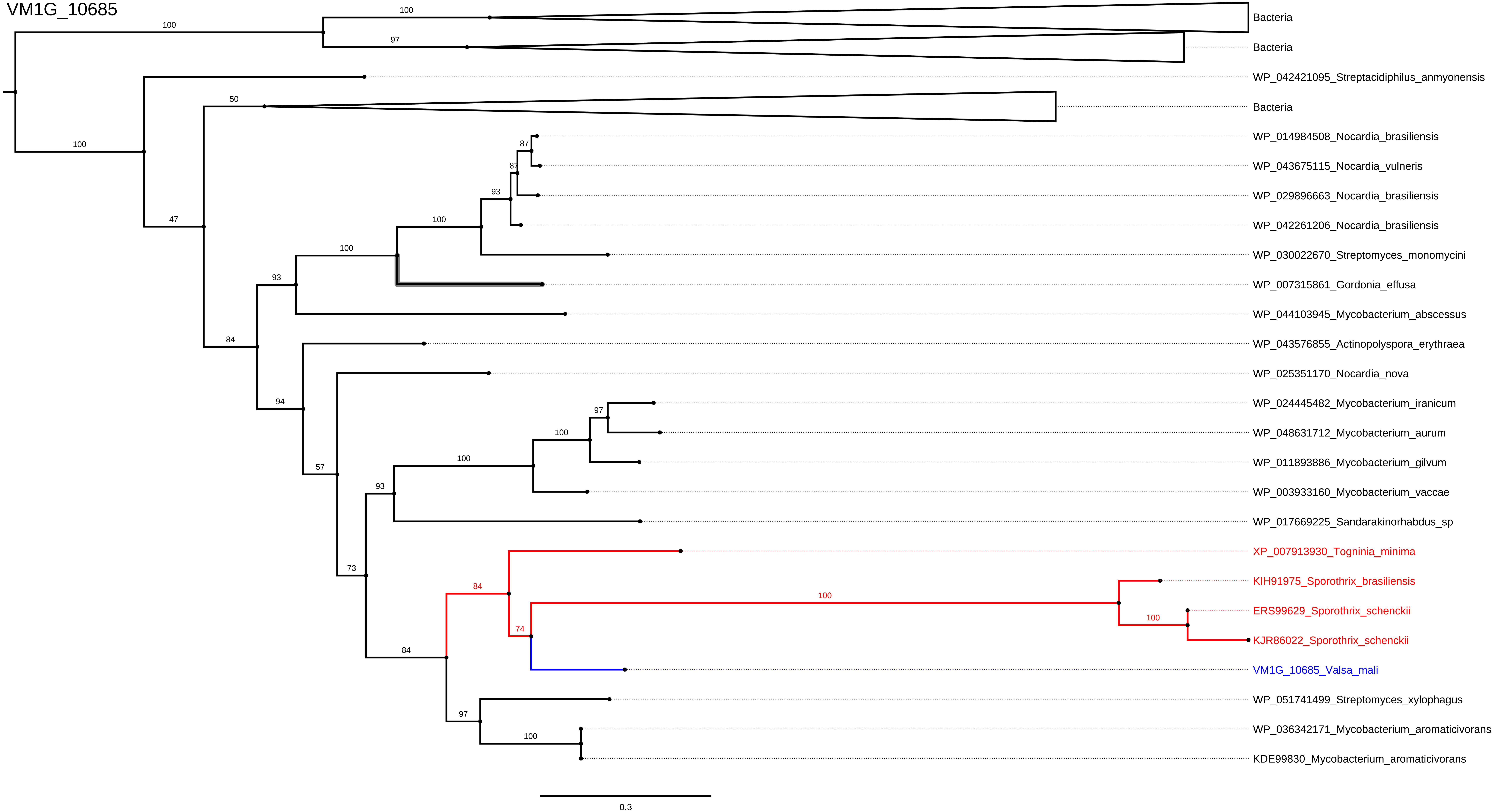
VM1G_10685
Bacteria
Bacteria
WP_042421095_Streptacidiphilus_anmyonensis
Bacteria
WP_014984508_Nocardia_brasiliensis
WP_043675115_Nocardia_vulneris
WP_029896663_Nocardia_brasiliensis
WP_042261206_Nocardia_brasiliensis
WP_030022670_Streptomyces_monomycini
WP_007315861_Gordonia_effusa
WP_044103945_Mycobacterium_abscessus
WP_043576855_Actinopolyspora_erythraea
WP_025351170_Nocardia_nova
WP_024445482_Mycobacterium_iranicum
WP_048631712_Mycobacterium_aurum
WP_011893886_Mycobacterium_gilvum
WP_003933160_Mycobacterium_vaccae
WP_017669225_Sandarakinorhabdus_sp
XP_007913930_Togninia_minima
KIH91975_Sporothrix_brasiliensis
ERS99629_Sporothrix_schenckii
KJR86022_Sporothrix_schenckii
VM1G_10685_Valsa_mali
WP_051741499_Streptomyces_xylophagus
WP_036342171_Mycobacterium_aromaticivorans
KDE99830_Mycobacterium_aromaticivorans
0.3

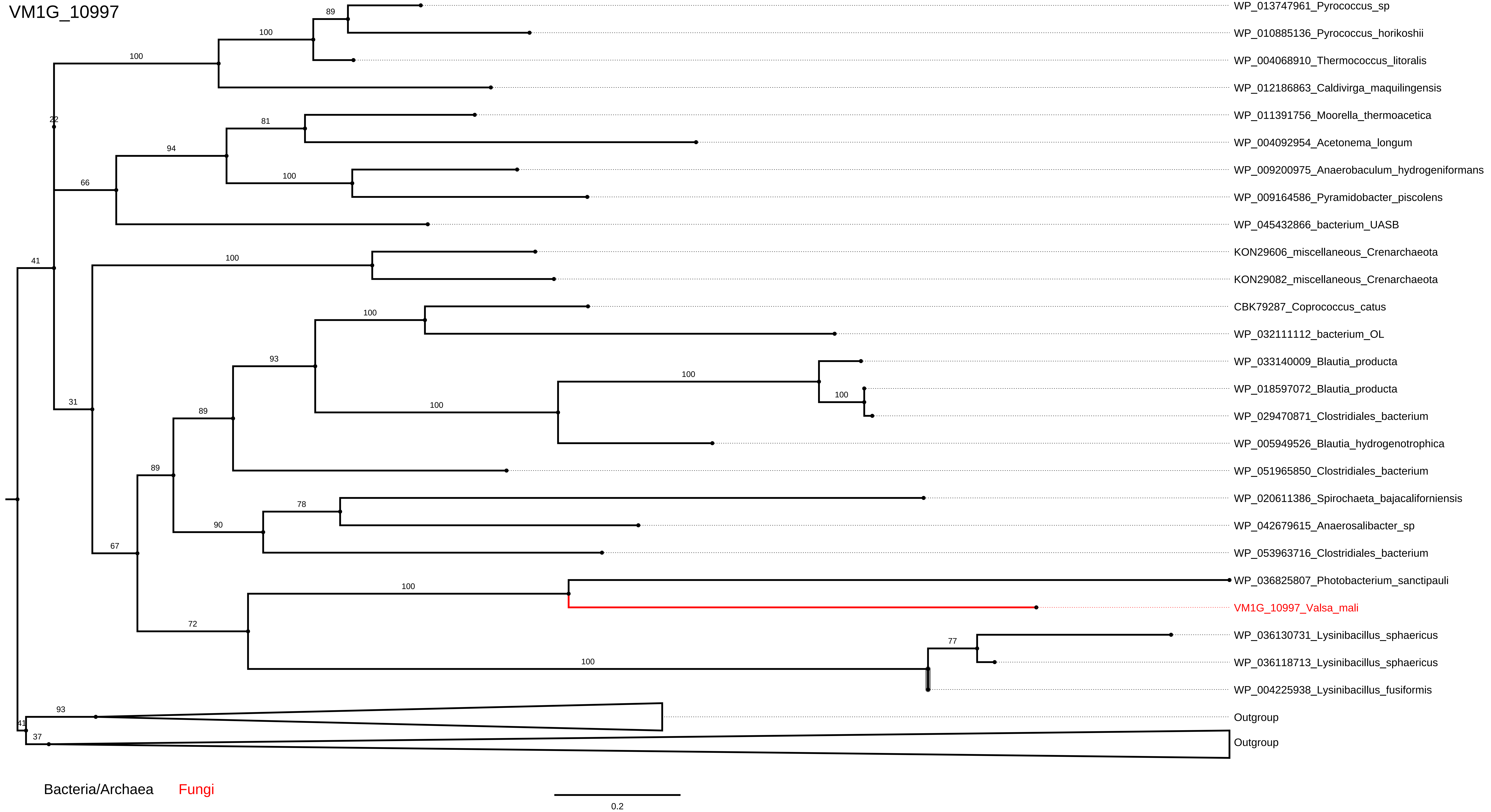
VM1G_10997
WP_013747961_Pyrococcus_sp
WP_010885136_Pyrococcus_horikoshii
WP_004068910_Thermococcus_litoralis
WP_012186863_Caldivirga_maquilingensis
WP_011391756_Moorella_thermoacetica
WP_004092954_Acetonema_longum
WP_009200975_Anaerobaculum_hydrogeniformans
WP_009164586_Pyramidobacter_piscolens
WP_045432866_bacterium_UASB
KON29606_miscellaneous_Crenarchaeota
KON29082_miscellaneous_Crenarchaeota
CBK79287_Coprococcus_catus
WP_032111112_bacterium_OL
WP_033140009_Blautia_producta
WP_018597072_Blautia_producta
WP_029470871_Clostridiales_bacterium
WP_005949526_Blautia_hydrogenotrophica
WP_051965850_Clostridiales_bacterium
WP_020611386_Spirochaeta_bajacaliforniensis
WP_042679615_Anaerosalibacter_sp
WP_053963716_Clostridiales_bacterium
WP_036825807_Photobacterium_sanctipauli
VM1G_10997_Valsa_mali
WP_036130731_Lysinibacillus_sphaericus
WP_036118713_Lysinibacillus_sphaericus
WP_004225938_Lysinibacillus_fusiformis
Outgroup
Outgroup
Bacteria/Archaea
Fungi
0.2

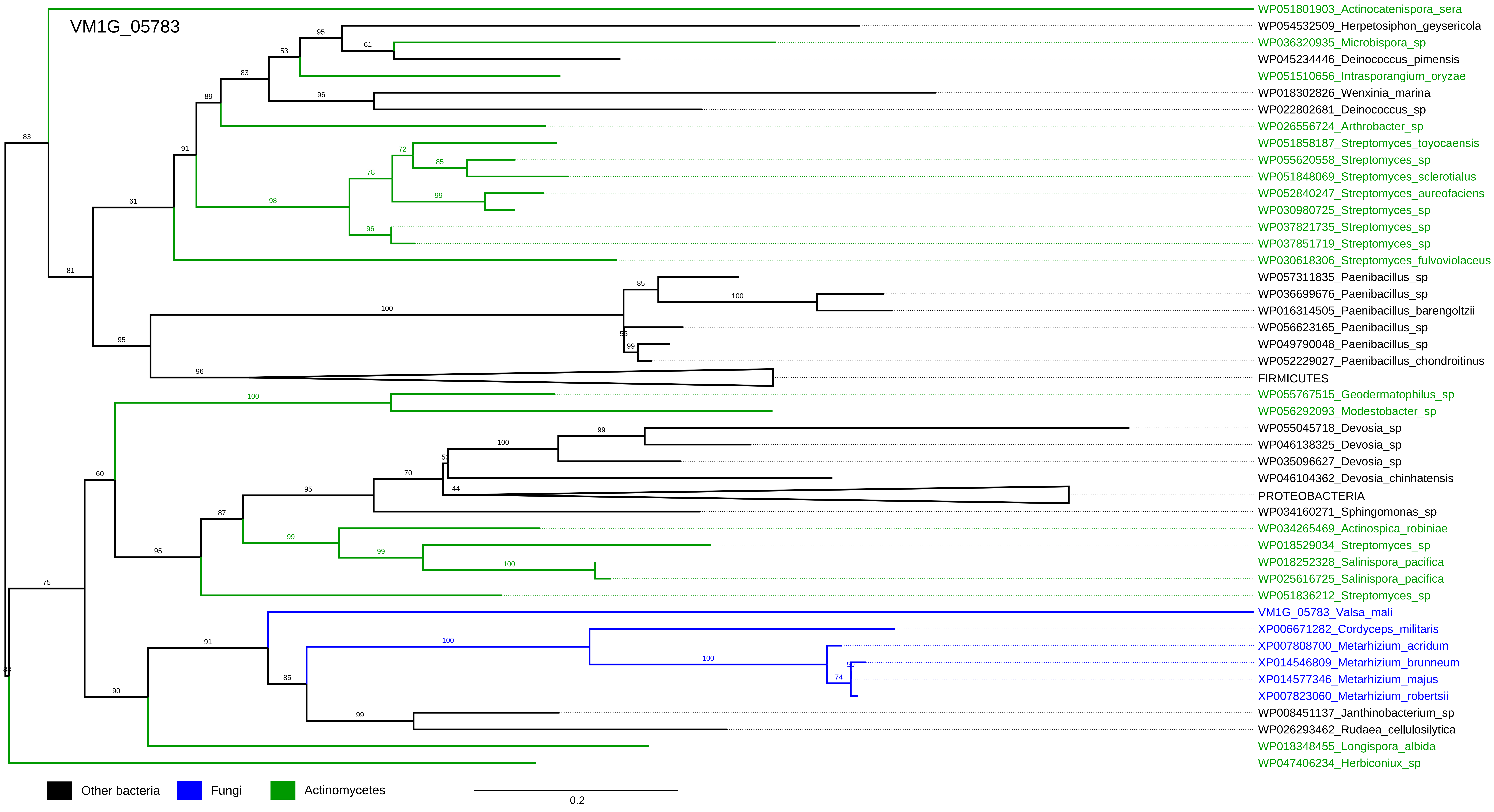
VM1G_05783
WP051801903_Actinocatenispora_sera
WP054532509_Herpetosiphon_geysericola
WP036320935_Microbispora_sp
WP045234446_Deinococcus_pimensis
WP051510656_Intrasporangium_oryzae
WP018302826_Wenxinia_marina
WP022802681_Deinococcus_sp
WP026556724_Arthrobacter_sp
WP051858187_Streptomyces_toyocaensis
WP055620558_Streptomyces_sp
WP051848069_Streptomyces_sclerotialus
WP052840247_Streptomyces_aureofaciens
WP030980725_Streptomyces_sp
WP037821735_Streptomyces_sp
WP037851719_Streptomyces_sp
WP030618306_Streptomyces_fulvoviolaceus
WP057311835_Paenibacillus_sp
WP036699676_Paenibacillus_sp
WP016314505_Paenibacillus_barengoltzii
WP056623165_Paenibacillus_sp
WP049790048_Paenibacillus_sp
WP052229027_Paenibacillus_chondroitinus
FIRMICUTES
WP055767515_Geodermatophilus_sp
WP056292093_Modestobacter_sp
WP055045718_Devosia_sp
WP046138325_Devosia_sp
WP035096627_Devosia_sp
WP046104362_Devosia_chinhatensis
PROTEOBACTERIA
WP034160271_Sphingomonas_sp
WP034265469_Actinospica_robiniae
WP018529034_Streptomyces_sp
WP018252328_Salinispora_pacifica
WP025616725_Salinispora_pacifica
WP051836212_Streptomyces_sp
VM1G_05783_Valsa_mali
XP006671282_Cordyceps_militaris
XP007808700_Metarhizium_acridum
XP014546809_Metarhizium_brunneum
XP014577346_Metarhizium_majus
XP007823060_Metarhizium_robertsii
WP008451137_Janthinobacterium_sp
WP026293462_Rudaea_cellulosilytica
WP018348455_Longispora_albida
WP047406234_Herbiconiux_sp
Other bacteria
Fungi
Actinomycetes
0.2
